# Inactivation of SmeSyRy Two-Component Regulatory System Inversely Regulates the Expression of SmeYZ and SmeDEF Efflux Pumps in *Stenotrophomonas maltophilia*

**DOI:** 10.1371/journal.pone.0160943

**Published:** 2016-08-11

**Authors:** Chao-Jung Wu, Yi-Wei Huang, Yi-Tsung Lin, Hsiao-Chen Ning, Tsuey-Ching Yang

**Affiliations:** 1 Department of Biotechnology and Laboratory Science in Medicine, National Yang-Ming University, Taipei, 112, Taiwan; 2 Division of Infectious Diseases, Department of Medicine, Taipei Veterans General Hospital, Taipei, 112, Taiwan; 3 School of Medicine, National Yang-Ming University, Taipei, 112, Taiwan; 4 Department of Laboratory Medicine, Chang Gung Memorial Hospital Linkou Branch, Taoyuan, Taiwan; 5 Department of Medical Biotechnology and Laboratory Science, Chang Gung University, Taoyuan, Taiwan; Centre National de la Recherche Scientifique, Aix-Marseille Université, FRANCE

## Abstract

SmeYZ efflux pump is a critical pump responsible for aminoglycosides resistance, virulence-related characteristics (oxidative stress susceptibility, motility, and secreted protease activity), and virulence in *Stenotrophomonas maltophilia*. However, the regulatory circuit involved in SmeYZ expression is little known. A two-component regulatory system (TCS), *smeRySy*, transcribed divergently from the *smeYZ* operon is the first candidate to be considered. To assess the role of SmeRySy in *smeYZ* expression, the *smeRySy* isogenic deletion mutant, KJΔRSy, was constructed by gene replacement strategy. Inactivation of *smeSyRy* correlated with a higher susceptibility to aminoglycosides concomitant with an increased resistance to chloramphenicol, ciprofloxacin, tetracycline, and macrolides. To elucidate the underlying mechanism responsible for the antimicrobials susceptibility profiles, the SmeRySy regulon was firstly revealed by transcriptome analysis and further confirmed by quantitative real-time polymerase chain reaction (qRT-PCR) and promoter transcription fusion constructs assay. The results demonstrate that inactivation of *smeRySy* decreased the expression of SmeYZ pump and increased the expression of SmeDEF pump, which underlies the *ΔsmeSyRy*-mediated antimicrobials susceptibility profile. To elucidate the cognate relationship between SmeSy and SmeRy, a single mutant, KJΔRy, was constructed and the complementation assay of KJΔRSy with *smeRy* were performed. The results support that SmeSy-SmeRy TCS is responsible for the regulation of *smeYZ* operon; whereas SmeSy may be cognate with another unidentified response regulator for the regulation of *smeDEF* operon. The impact of inverse expression of SmeYZ and SmeDEF pumps on physiological functions was evaluated by mutants construction, H_2_O_2_ susceptibility test, swimming, and secreted protease activity assay. The increased expression of SmeDEF pump in KJΔRSy may compensate, to some extents, the SmeYZ downexpression-mediated compromise with respect to its role in secreted protease activity.

## Introduction

To get rid of toxic substances and waste products, bacteria are equipped with efficient efflux systems. The efflux systems contributing to multidrug resistance have been extensively reported in several bacterial pathogens [1]. On the basis of structural characteristics, the multidrug efflux systems are classified into five families: resistance nodulation cell division (RND), major facilitator superfamily (MFS), small multidrug resistance (SMR), multidrug and toxic compound extrusion (MATE), and ATP-binding cassette (ABC) [2]. Among them, RND transporter is an effective mediator of multi-drug resistance in Gram-negative bacteria. The RND efflux pump forms a tripartite transporter system comprised of an inner membrane protein (IMP), an outer membrane protein (OMP), and a membrane fusion protein (MFP) to link IMP and OMP [3].

The known determinants involved in the regulation of RND efflux pumps include local regulator and global regulator [4]. The local regulators genes generally locate next to one of the regulated genes and can be easily recognized; however, the genes encoding the global regulators situate chromosomally elsewhere. In general, there are two kinds of regulatory systems involved in the expression of the multidrug efflux systems; one is the transcriptional regulator and the other is the two-component regulatory system (TCS). TCSs classically consist of an inner membrane-spanning sensor kinase (SK) and a cytoplasmic response regulator (RR) [5]. The SK and RR genes are often encoded adjacent to one another in the genome, forming an operon. Typically, a specific signal triggers the SK, which undergoes autophosphorylation at a specific histidine residue. This phosphoryl group is then transferred to an aspartate residue of the cognate RR, resulting in its activation. The activated RR usually acts as a transcriptional regulator to alter the genes expression.

*Stenotrophomonas maltophilia*, an opportunistic human pathogen, is well known for its intrinsic resistance to a wide range of antibiotics, including β-lactam, quinolone, and aminoglycoside [6]. Multi-drug resistances of *S*. *maltophilia* have been attributed to expression of antibiotic hydrolyzing or modifying enzymes [7–8] and multidrug efflux systems [9–14]. Eight putative RND-type efflux systems, SmeABC, SmeDEF, SmeGH, SmeIJK, SmeMN, SmeOP, SmeVWX, and SmeYZ, have been revealed in the sequenced genome of *S*. *maltophilia* K279a [15]. Of them, six pumps, SmeABC, SmeDEF, SmeIJK, SmeOP, SmeVWX, and SmeYZ, have been characterized [9–14]. The genome organization of the six pump operons and their individually contiguous regulatory genes are summarized in S1 Fig. The SmeABC pump is intrinsically quiescent [9]. The *smeRS* TCS divergently located upstream of *smeABC* is involved in the *smeABC* overexpression. The *smeDEF* and *smeOP* operons are negatively regulated by the products of the *smeT* and *smeRo* genes, which encode the TetR-type transcriptional repressors. *SmeT* and *smeRo* are located upstream of the *smeDEF* and *smeOP* operons and are divergently transcribed [12, 16]. *SmeDEF* and sm*eOP* operons are expressed and can be further overexpressed by *smeT* and *smeRo* inactivation, respectively [12, 16]. The substrates extruded by SmeDEF pump are mainly chloramphenicol, quinolone, tetracycline, and macrolide [10]. SmeOP pump can extrude nalidixic acid, doxycycline, aminoglycosides, and macrolides [12]. SmeVWX pump is intrinsically quiescent and its expression is positively regulated by the divergently transcribed *smeRv*, which encodes a LysR-type transcription regulator [13]. SmIJK- and SmeYZ-overexpression strains contribute the resistance to aminoglycosides/leucomycin and aminoglycosides/trimethoprim-sulfamethoxazole, respectively [11, 17]. There is no recognizable regulatory gene flanking the *smeIJK* operon. However, we noticed that a TCS (designated as *smeSy* and *smeRy*), located upstream of *smeYZ* operon. This genomic organization highly suggests that SmeRySy TCS is responsible for the expression of *smeYZ* operon. In this study, we assessed the regulatory role of the SmeRySy TCS in the expression of SmeYZ. Unexpectedly, we found that the SmeDEF pump was upexpressed in the case of SmeRySy TCS inactivation, and the underlying regulatory circuit was further elucidated.

## Materials and Methods

### Bacterial strains, primers, and growth condition

S1 Table summarizes bacterial strains, plasmids, and primers used in this study. The strains used were derivatives of *S*. *maltophilia* KJ [8]. All primers used in this study were designed based on the *S*. *maltophilia* K279a genome sequence [15]. For the general purpose, strains were grown aerobically in Luria-Bertani (LB) medium except special note.

### Construction of deletion mutants

To construct *ΔsmeT*, *ΔsmeDEF*, *ΔsmeRy*, and *ΔsmeRSy* mutants, the expected 454-, 406-, 820-, 503-, 390-, and 580-bp products were amplified from the genome of *S*. *maltophilia* KJ using primers SmeT3-F/R, SmeD5-F/R, SmeF3-F/R, SmeSy3-F/R, SmeRy3-F/R, and SmeRy5-F/R (S2 Fig). The 454-bp and 406-bp PCR products were subsequently cloned into the vector pEX18Tc, yielding plasmid pΔT, similarly, the amplicons of 406-bp and 820-bp for the construct of pΔDEF, the amplicons of 503-bp and 580-bp for pΔRSy, and the amplicons of 390-bp and 580-bp for pΔRy. The plasmids pΔT, pΔDEF, pΔRSy, and pΔRy were introduced into *E*. *coli* S17-1 by transformation and mobilized into *S*. *maltophilia* KJ via conjugation [18]. Transconjugants carrying deleted *smeT*, *smeDEF*, *smeRSy*, and *smeRy* in the chromosome were obtained by two-step selection on LB agar containing tetracycline (30 μg/ml)/norfloxacin (2.5 μg/ml) and then LB agar containing 10% (wt/vol) sucrose, yielding the deletion mutants KJΔT, KJΔDEF, KJΔRSy, and KJΔRy respectively (S2 Fig). The correctness of mutants was confirmed by colony PCR [19].

### Construction of promoter-*xylE* transcription fusions

The aforementioned 406-bp PCR amplicon (primered by SmeD5-F and SmeD5-R) was treated with SmaI. The resulting 359-bp SmaI-SmaI DNA fragment, encompassing the 224-bp *smeT*-*smeD* intergenic region, was cloned into the pRK415 at both directions respectively. A *xylE* gene retrieved from pTxylE [8] was subsequently cloned behind the 224-bp DNA fragment, to generate *smeT* and *smeDEF* promoter transcription fusions constructs, pSmeT_xylE_ and pSmeD_xylE_ (Figure A in S2 Fig). The orientation of *xylE* gene inserted was opposite to that of the *lacZ*’ promoter of pRK415. A 343-bp DNA fragment spanning nucleotide -310 to +34 relative to the *smeY* start codon was obtained by PCR using primer sets SmeY5-F and SmeY5-R. We cloned this amplicon in front of the *xylE* reporter gene in a pRKXylE vector [11], yielding a *smeYZ* promoter transcription fusion construct, pSmeY_xylE_ (Figure B in S2 Fig).

### Transcriptome assay by RNA sequencing

Overnight-cultured bacteria cultures were inoculated into fresh LB broth with an initial OD_450nm_ of 0.15. All cultures were grown at 37°C for 5 h and DNA-free RNA was extracted as described previously [11]. Ribosomal RNA (rRNA) was depleted from 5 μg of total RNA using the Ribo-Zero rRNA Removal Kit for bacteria (Epicentre, USA). The sequencing library for mRNA-seq was prepared according to the protocol for the "TruSeq RNA sample preparation” (Illumina Inc., USA). Briefly, the rRNA depleted mRNA was fragmented, and first-strand cDNA was synthesized using random hexamers following by second-strand cDNA synthesis, end repair, addition of a single A base and adapter ligation. The adapter-ligated cDNA library was amplified by PCR for 6 cycles using KAPA HiFi DNA polymerase (Kapa Biosystems). The enriched cDNA library was sequenced on a MiSeq (Illumina Inc., USA) using 250 bp paired-end reads. After trimming of low quality of bases (< Q30), the first 12 bases and adapters, the trimmed Reads were mapped to the *Stenotrophomonas maltophilia* K279a genome (GenBank acc. no. NC_010943.1) and run RNA-seq analysis by CLC Genomics Workbench v 6.0 (CLC Bio).

### Antimicrobial susceptibility test

The susceptibilities of *S*. *maltophilia* strains to a number of antibiotics were tested by serial twofold dilutions in Mueller-Hinton agar according to the guidelines of Clinical Laboratory Standards Institute (CLSI) [20]. After overnight incubation at 37°C, cell growth was examined visually. The MIC was defined as the lowest concentration of antimicrobial agent that inhibited visible growth. All antibiotics were purchased from Sigma.

### Catechol 2,3-dioxygenase (C23O) activity assay

The catechol-2,3-dioxygenase activity was measured as described previously [21]. The rate of hydrolysis was calculated by using 44,000 M-1cm-1 as the extinction coefficient. One unit of enzyme activity (Uc) was defined as the amount of enzyme that converts 1 nmole substrate per minute. The specific activity was expressed as Uc/OD_450nm._

### Quantitative Real-Time PCR (qRT-PCR)

DNA-free cellular RNA was prepared [11] and its purity was checked by qPCR without the additive of reverse transcriptase. The RNA was converted into cDNA which was then used directly as a template for qRT-PCR [11]. The primers used for qRT-PCR are listed in S1 Table. Amplification and detection of specific products were performed in the ABI Prism 7000 Sequence Detection System (Applied Biosystems) using the Smart Quant Green Master Mix (Protech Technology Enterprise Co., Ltd.) according to the manufacturer’s protocols. The mRNA of 16S rDNA was chosen as the internal control to normalize the relative expression level. Relative quantities of mRNA from each gene of interest were determined by the comparative cycle threshold method [22]. Each experiment was performed in triplicate.

### H_2_O_2_ susceptibility test, swimming, and secreted protease activity assay

The physiological functions, including H_2_O_2_ susceptibility test, swimming, and secreted protease activity assay, were determined following the established protocols [17]. Each experiment was performed with at least 3 replicates. Results shown are mean with standard deviations. Statistical significance was assessed by Student *t* test.

## Results and Discussion

### Sequence analysis of the *smeRySy-smeYZ* cluster

The genetic organization surrounding the *smeYZ* genes (Smlt2198 and Smlt2197) in the genome of *S*. *maltophilia* K279a was surveyed. Smlt2200 and Smlt2199, transcribed divergently from the *smeYZ*, were found immediately upstream of *smeYZ* (Figure B in S2 Fig). The coding regions of Smlt2200 and Smlt2199 overlapped, signifying that they comprise a co-transcribed operon. Reverse transcriptase-PCR (RT-PCR) was carried out to verify the presence of Smlt2200-Smlt2199 operon (S3 Fig). The regulatory regions and relevant consensus sequences of Smlt2200 and Smlt2199 proteins were analyzed. The relevant domains, linked to the functions of sensor kinase and response regulator of a TCS, were identified in SmeSy and SmeRy, respectively (S4 Fig). In addition, based on the comparisons with other sensor kinases and response regulators, the His^148^ residue of SmeSy and the Asp^81^ residue of SmeRy likely represent the autophosphorylated histidine and the phosphoaceptor aspartate, respectively (S4 Fig). Herein, we designated the Smlt2200 and Smlt2199 as SmeRy and SmeSy, respectively.

### Inactivation of SmeRySy system decreases the expression of *smeYZ* operon

**Fig 1:**
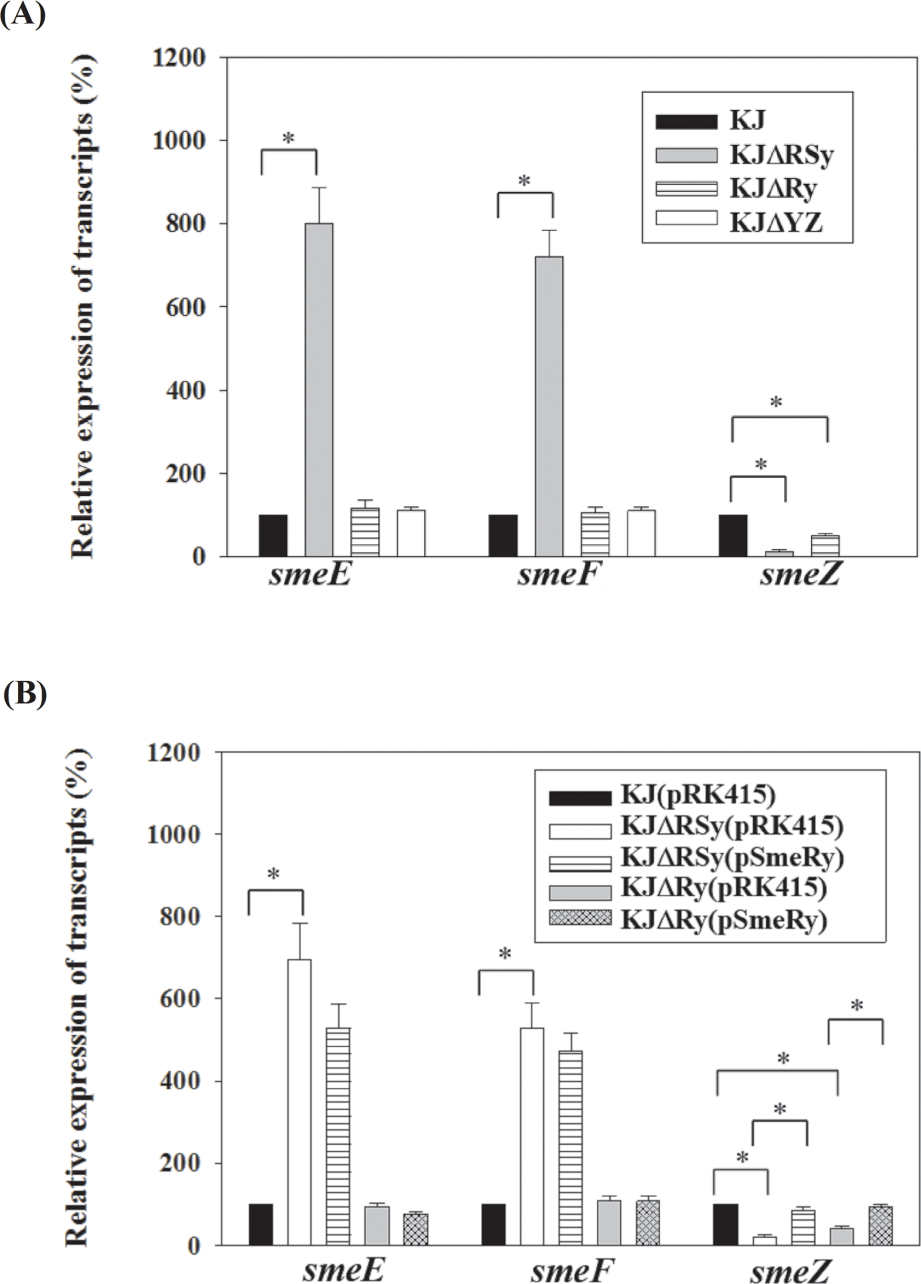
Quantitative RT-PCR (qRT-PCR) analysis of *smeE*, *smeF*, and *smeZ* transcripts. The RNA was prepared from the exponentially growing cells, converted to cDNA by RT-PCR, and used as the template for quantitative reverse-transcriptase PCR (qRT-PCR). Data are the means of three independent experiments. Error bars indicate the standard deviation for three triplicate samples. *, *p*≤0.05 significance calculated by a Student’s *t*-test. (A) qRT-PCR analysis of *smeE*, *smeF*, and *smeZ* transcripts of strains KJ, KJΔRSy, KJΔRy, and KJΔYZ. (B) qRT-PCR analysis of *smeE*, *smeF*, and *smeZ* transcripts in the *smeRy*-complemented strains of KJΔRSy and KJΔRy.

**Fig 2:**
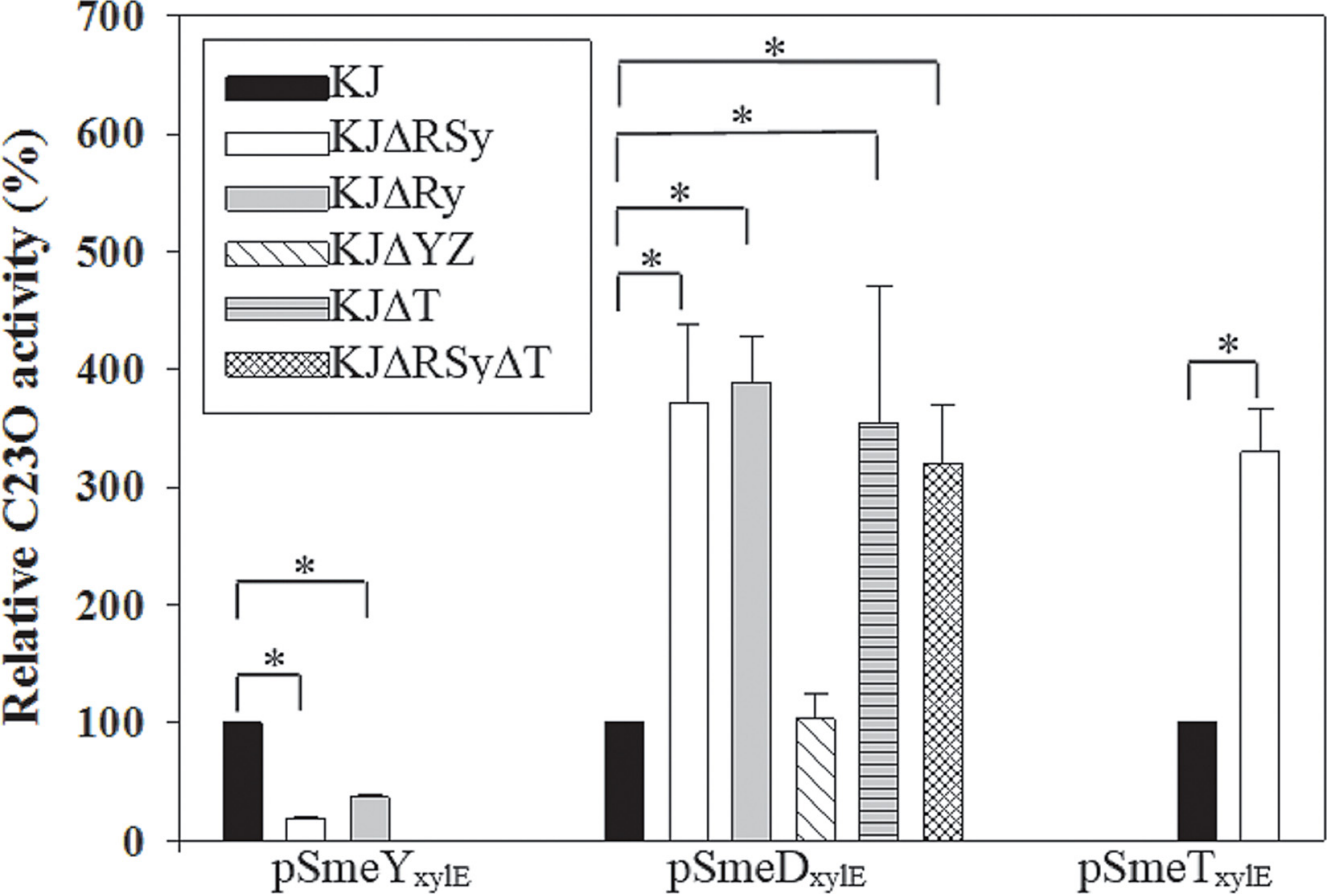
The C23O activities expressed by the promoter transcription fusion constructs of pSmeY_xylE_, pSmeD_xylE_, and pSmeY_xylE_ in wild-type KJ and its derived mutants. The overnight-cultured bacteria assayed were inoculated into fresh LB with an initial OD_450nm_ of 0.15, cultured for 5 h, and the C23O activities were determined. The data represent means of three repetitions. Error bars indicate the standard deviation for three triplicate samples. *, *p*≤0.05 significance calculated by a Student’s *t*-test.

SmeRySy TCS is divergently transcribed from the *smeYZ* operon. To test the regulatory role of SmeRySy TCS in the expression of *smeYZ* operon, *smeRySy* was deleted from the chromosome of wild-type strain KJ and the impacts on antimicrobial susceptibility and *smeYZ* expression were examined. The resulting strain, KJΔRSy, was more susceptible to all of the SmeYZ substrates tested (Table 1) [17], suggesting that SmeRySy inactivation decreases the expression of *smeYZ* operon. The *smeYZ* expression in KJΔRSy was further checked by qRT-PCR. Compared with those in wild-type KJ, the *smeZ* transcript in KJΔRSy was decreased (Fig 1). Decreased transcription of the *smeYZ* operon in KJΔRSy versus KJ was also confirmed by the promoter transcription fusion assay. Fig 2 demonstrates that the promoter activity of *smeYZ* operon was drastically decreased in KJΔRSy (pSmeY_xylE_ in KJ and KJΔRSy), further confirming the positive regulatory role of SmeRySy TCS in the expression of *smeYZ* operon. However, it is worthily mentioned that the expression of SmeYZ pump was not totally abolished in the case of SmeRySy inactivation. The MICs reduction in KJΔRSy did not reach the level displayed in KJΔYZ (Table 1), and there was detectable *smeZ* transcript in KJΔRSy (Fig 1) and detectable C23O activity in the strain KJΔRSy(pSmeY_xylE_) (Fig 2), indicating that a remnant expression of *smeYZ* pump exists in KJΔRSy.

**Table 1:** Antimicrobial susceptibilities of *S*. *maltophilia* KJ and its derived constructs.

	MIC (μg/ml)							
	CHL	CIP	TET	ERY	LM	KAN	GEN	TOB
KJ	8	1	16	64	256	256	1024	512
KJΔRSy	16	4	32	256	512	32	32	32
KJΔRSyΔDEF	4	0.5	8	32	128	32	32	32
KJΔDEF	4	0.5	8	32	128	256	1024	512
KJΔYZ	8	1	8	128	128	8	8	32
KJΔRy	8	1	16	64	256	32	32	32
KJΔT	32	8	32	512	2048	64	128	64
KJΔRSyΔT	32	8	32	512	1024	4	8	16
KJΔRSy(pSmeRy)	16	4	-^a^	256	512	64	256	512
KJΔ Ry(pSmeRy)	8	1	-^a^	65	256	256	1024	512

CHL, chloramphenicol; CIP, ciprofloxacin; TET, tetracycline; ERY, erythromycin; LM, leucomycin; KAN, kanamycin; GEN, gentamicin; TOB, tobramycin.

^a^The complementation plasmid carries the tetracycline-resistant gene

### Inactivation of SmeRySy system increases the expression of *smeDEF* operon

In the results of susceptibility test of KJΔRSy, it was surprisingly noticed that the MICs of KJΔRSy to chloramphenicol, ciprofloxacin, tetracycline, and macrolides were increased (Table 1), and these antimicrobials are not the substrates of the SmeYZ pump [17]. It is thus likely that in addition to *smeYZ* operon, the SmeRySy system regulates other antimicrobials resistance-relevant determinants, which are responsible for the increased MICs of chloramphenicol, ciprofloxacin, tetracycline, and macrolides. We sought to determine genes that comprise the SmeRySy regulon by comparing transcription in wild-type KJ and *smeRySy* mutant, KJΔRSy. The transcriptomic data showed that the difference in gene expression caused by the *smeRySy* deletion was small, with only 21 genes showing greater than a 3-fold change (up or down) in their transcript abundance (Table 2; S2 Table). Interestingly, the greatest dysregulation was displayed by two RND-type efflux pump operons, *smeYZ* and *smeDEF*. The expression of *smeDEF* and *smeYZ* was inversely changed in response to the *smeRySy* inactivation (Table 2). To validate the expression profiles obtained by the transcriptome analysis, the *smeE*, *smeF*, and *smeZ* transcripts in KJ and KJΔRSy were quantified by qRT-PCR. Fig 1 shows that *smeDEF* and *smeYZ* operons were upregulated and downregulated, respectively, in response to *smeSyRy* inactivation, which is consistent with the RNA-seq data. It has been known the substrates extruded by the SmeDEF pump include chloramphenicol, ciprofloxacin, tetracycline, and macrolide [10]. Therefore, it is highly suggested that the *smeRySy*-mediated *smeDEF* upregulation is responsible for the increased MICs observed in KJΔRSy (Table 1).

**Table 2:** Transcriptomic analysis of genes differentially expressed in the *smeRySy* mutant compared to the wild-type *S*. *maltophilia* KJ.

Gene ID	Description / gene name	Fold change^a^
Smlt4071	SmeE (RND-type multidrug efflux pump)	11.036
Smlt4070	SmeF (RND-type multidrug efflux pump)	10.410
Smlt4072	SmeD (RND-type multidrug efflux pump)	9.576
Smlt3594	transmembrane protein	6.115
Smlt4069	transmembrane protein	3.655
Smlt0271	hypothetical protein	3.057
Smlt2932	glutamine amidotransferase class-I	3.019
Smlt4073	SmeT	2.718
Smlt2201	SmeY(RND-type multidrug efflux pump)	-19.440
Smlt2202	SmeZ (RND-type multidrug efflux pump)	-9.273
Smlt1290	conjugal transfer protein / *trbC*	-5.738
Smlt1284	conjugal transfer protein /*trbG*	-5.734
Smlt2200	SmeRy	-5.072
Smlt2203	Hypothetical protein	-4.641
Smlt2531	hypothetical protein	-4.568
Smlt1419	transmembrane protein	-4.055
Smlt0593	methionine sulfoxide reductase	-3.891
Smlt2283	flagellarbasal body-associated protein /*fliL*	-3.634
Smlt2290	flagellar MS-ring protein / *fliE*	-3.577
Smlt1293	conjugal transfer coupling protein TraG/*traG*	-3.478
Smlt2312	flagellar basal body rod protein FlgG/ *flgG*	-3.418
Smlt2314	flagellar hook protein FlgE/ *flgE*	-3.371

^a^A positive value signifies upregulation and a negative value signifies downregulation in *smeRySy* mutant.

To validate the SmeRySy TCS regulatory role in *smeDEF* and *smeYZ* operons, the promoter transcription fusions of pSmeD_xylE_ and pSmeY_xylE_ were prepared. The C23O activities expressed by pSmeD_xylE_ and pSmeY_xylE_ in strains KJ and KJΔRSy were comparatively determined. KJΔRSy(pSmeD_xylE_) had a ca. 3.72-fold increase in C23O activity relative to KJ(pSmeD_xylE_); and the C23O activity expressed by KJΔRSy(pSmeY_xylE_) was 82% lower than that by KJ(pSmeY_xylE_) (Fig 2), in consistent with the transcriptome result.

To determine whether upregulation of *smeDEF* in KJΔRSy may account for the increased antimicrobials resistance (Table 1), the *smeDEF* operon was deleted from the chromosome of strain KJΔRSy, yielding KJΔRSyΔDEF. The MICs of KJΔRSyΔDEF to chloramphenicol, ciprofloxacin, tetracycline, and macrolides were reverted to the comparable level as those of KJΔDEF (Table 1); but the MICs of KJΔRSyΔDEF to aminoglycosides were still as low as those of KJΔRSy. Taken together, we concluded that inactivation of the *smeRySy* system results in the upexpression of *smeDEF* operon, which then confers to the phenotype of increased resistance to chloramphenicol, ciprofloxacin, tetracycline, and macrolides.

### *SmeYZ* is irrelevant to the *ΔsmeRySy*-mediated upexpression of *smeDEF*

It has been demonstrated that altering the expression of a single RND pump may have downstream effect on any number of other RND efflux systems [23]. In view of the regulatory role of SmeRySy TCS in *smeYZ* operon, we wondered whether the increased *smeDEF* transcript in strain KJΔRSy results from the coordinated regulation between the SmeYZ pump and SmeDEF pump, other than from the direct regulation of the SmeRySy system. To solve the question, the C23O activity expressed by pSmeD_xylE_ was comparatively assessed in KJ and KJΔYZ cells. The promoter activity of *smeDEF* operon was little affected in the case of *smeYZ* inactivation (Fig 2). Furthermore, Table 1 also shows that KJΔYZ exhibited a similar degree of resistance to chloramphenicol, ciprofloxacin, tetracycline and macrolides, compared with wild-type KJ, further supporting that the elevated resistance in KJΔRSy is not simply compensatory upregulation in response to decrease expression of SmeYZ.

### *SmeRy* is not involved in the *ΔsmeRySy*-mediated increment of *smeDEF* transcript and antibiotic resistance

To further elucidate the SmeSy and SmeRy cognate relationship responsible for the *ΔsmeRySy*-mediated phenotype, we deleted the *smeRy* gene in wild-type KJ, yielding KJΔRy. The antibiotic susceptibility and the expression of *smeDEF* and *smeYZ* operons in KJΔRy were performed. KJΔRy increased the susceptibility toward aminoglycosides, which are the substrates of SmeYZ, but did not alter the susceptibility toward the antibiotics, which are the known substrates of SmeDEF pump (Table 1). The *smeZ* transcript and the promoter activity assay (*P*_*smeYZ*_) of KJΔRy were decreased relative to those of wild-type KJ (Fig 1A & 2). The *smeE* and *smeF* transcripts of KJΔRy were comparable to those of wild-type KJ (Fig 1), in despite of an increment in the *P*_*smeDEF*_ promoter activity of KJΔRy (Fig 2). These observations imply the irrelevance of SmeRy to *ΔsmeRySy*-mediated increment of *smeDEF* transcript and antibiotic resistance. To further verify this assumption, complementation assay was performed. Complementation of KJΔRSy or KJΔRy with a *smeRy* gene restored the resistance toward aminoglycosides (Table 1) and the *smeZ* transcript (Fig 1B), but little affected the susceptibility toward chloramphenicol, ciprofloxacin, tetracycline, and macrolides (Table 1) as well as the *smeE* and *smeF* transcripts (Fig 1B).

Based on these observations, we propose that SmeSy may cooperate with two different response regulators to individually regulate *smeDEF* and *smeYZ* operons. When SmeRy acts as a cognate response regulator of SmeSy, it can activate the expression of *smeYZ* and thus contribute to the SmeYZ pump-mediated resistance. Nevertheless, there should be another unidentified response regulator that can work with SmeSy to regulate *smeDEF* operon. In view of the increased *P*_*smeD*_ promoter activity (Fig 2) and the decreased *smeDEF* transcript (Fig 1A) in KJΔRy, SmeSy-mediated regulation in KJΔRy may link to the stability of *smeDEF* transcript. When *smeSy* and *smeRy* are simultaneously inactivated, the phenotype of SmeDEF pump upexpression and SmeYZ pump downexpression can coexist.

### The regulatory roles of SmeT and SmeRySy TCS in the expression of *smeDEF*

Since the expression of the *smeDEF* operon is under the negative control of the TetR-type transcriptional regulator SmeT, which is divergently transcribed from the *smeDEF* operon (S1 Fig) [16], we wondered whether SmeRySy TCS affects the expression of *smeT* and consequently alters the expression of *smeDEF* operon. Based on the transcriptome data, the *smeT* transcript had a 2.71-fold upregulation in *smeRSy* mutant compared with that in wild-type KJ (Table 2, S2 Table). To confirm this, the *smeT* promoter transcription fusion construct, pSmeT_xylE_, was transferred into wild-type KJ and KJΔRSy. As seen in Fig 2, the C23O activity expressed by KJΔRSy(pSmeT_xylE_) was increased compared to that by KJ(pSmeT_xylE_).

In view of upexpression of *smeDEF* operon in strains KJΔT and KJΔRSy, we further assessed whether simultaneous inactivation of *smeSyRy* and *smeT* has an additive effect on *smeDEF* upexpression. To assess this, the expressions of pSmeD_xylE_ in wild-type KJ, KJΔRSy, KJΔT, and KJΔRSyΔT were comparatively determined. The C23O activities determined from KJΔRSy(pSmeD_xylE_), KJΔT(pSmeD_xylE_), and KJΔRSyΔT(pSmeD_xylE_) were comparable (Fig 2). As a result, simultaneous inactivation of *smeRySy* and *smeT* did not further upregulate the expression of *smeDEF* operon. The antimicrobial susceptibilities of strains KJΔT and KJΔRSyΔT displayed a consistent result (Table 1).

In this article, we found that SmeRySy inactivation simultaneously upregulates the expression of *smeDEF* and *smeT* (Fig 2). This observation seems to contradict the previous report that the repression extent of *smeDEF* operon has a positive correlation with the amount of repressor SmeT [16]. Herein, we proposed two explanations for this phenotype. (i) The binding activity of transcriptions repressor toward DNA affects the repressor function [24]. Therefore, the *smeDE*F upexpression observed in SmeRySy inactivation may result from the compromise of SmeT-operator interaction and is less relevant to the amount of SmeT. It is highly possible that a modulator, whose expression is altered in the case of *smeRySy* inactivation, compromises the affinity between SmeT and operator, and thus simultaneously derepresses the expression of *smeDEF* and *smeT*. (ii) Both SmeT and the unidentified regulator have a direct effect and are needed for the repression of *smeDEF* operon, in which case the absence of one or another will produce the same effect on the *smeDEF* upregulated.

### SmeDEF upexpression in *ΔsmeRySy* compensates the SmeYZ downexpression-mediated alterations with respect to secreted protease activity

**Fig 3:**
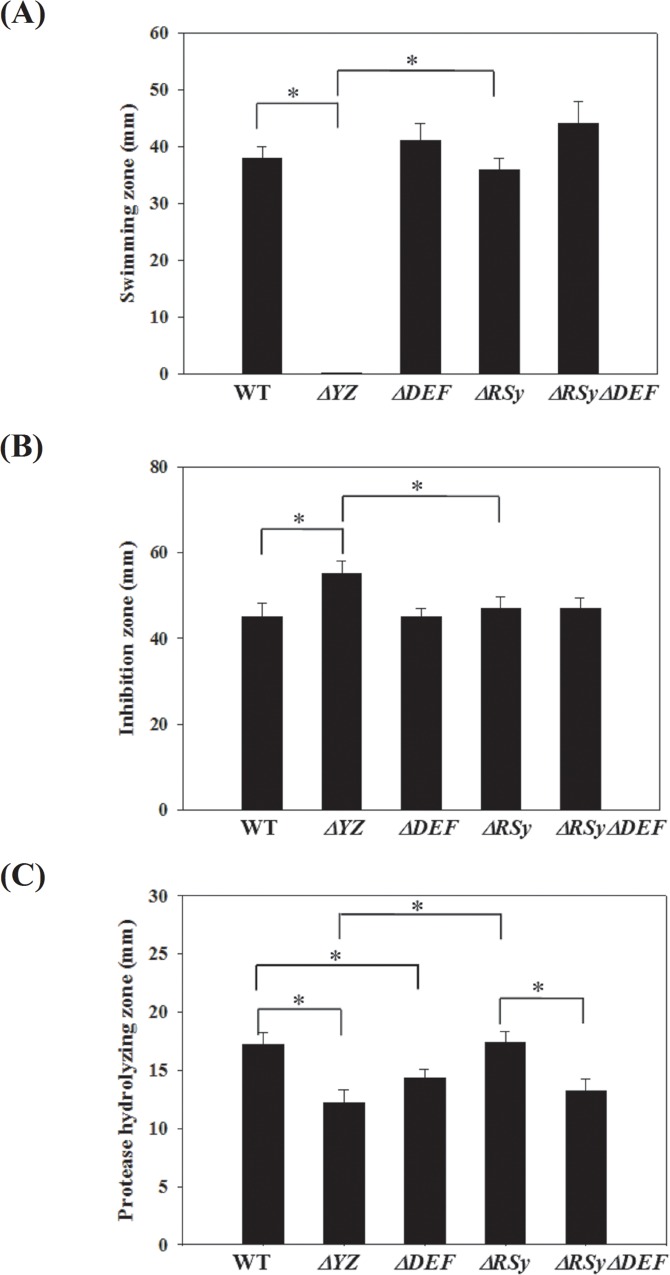
The physiological functions evaluation between wild-type KJ and its derived mutants. The data represent means of three repetitions. Error bars indicate the standard deviation for three triplicate samples. *, *p*≤0.05 significance calculated by a Student’s *t*-test. (A) Motility ability. Five microliter bacterial cell suspension was inoculated at the swimming agar (1% tryptone, 0.5% NaCl and 0.15% agar), and the diameter (mm) of swimming zone was measured after 48 h incubation at 37°C. (B) H_2_O_2_ susceptibility test. Sterile filter paper with 20 μl of 10% H_2_O_2_ was placed onto MH agar, which was uniformly spread with bacterial cell suspension. The diameter of a zone of growth inhibition was measured after 24 h incubation at 37°C. (C) Secreted protease activity assay. Forty microliter bacterial cell suspension was dipped on LB agar containing 1% skin milk. The proteolytic activity of bacteria was assessed by measuring the transparent zones around the bacteria after incubation at 37°C for 72 h.

The overall expression of MDR pumps is closely monitored, and whenever the levels of one of these systems are altered, compensatory changes in the levels of the other MDR pumps may follow. For example, there is an inverse correlation between the MexAB-OprM and MexEF-OprN expression in *P*. *aeruginosa* [23]. Inactivation of SmeRySy TCS inversely regulates the expression of SmeDEF and SmeYZ pumps, implying that there are some overlapped functions between SmeDEF and SmeYZ, and the inverse expression between both pumps may have a partially compensatory effect benefiting bacterial survival. Given the distinct difference in substrate profiles of SmeDEF and SmeYZ pumps (Table 1), the compensatory effect is less relevant to antibiotics extrusion. In our recent study, we have demonstrated that the SmeYZ pump contributes to an array of physiological functions, including oxidative stress susceptibility, swimming, and secreted protease activity [17]. Therefore, we were interested in assessing whether SmeDEF upexpression in *ΔsmeRySy* compensates the SmeYZ downexpression-mediated compromise of physiological functions. The physiological functions for oxidative stress susceptibility, swimming, and secreted protease activity in strains KJ, KJΔYZ, KJΔDEF, KJΔRSy, and KJΔRSyΔDEF were comparatively evaluated. Consistent with the previous reports [17], KJΔYZ displayed a compromise in oxidative stress susceptibility, swimming, and secreted protease activity; however, these compromises in KJΔRSy were not as severe as those in KJΔYZ, as compared with wild-type KJ (Fig 3). This observation suggested two possibilities: (i) Small level of SmeYZ expression could still mediate certain functions, since there are remnant expression of SmeYZ pump in KJΔRSy (Fig 1 & 2); (ii) some compensatory mechanisms for the physiological functions of SmeYZ pump may occur in KJΔRSy. As shown in Fig 3A, mutant KJΔYZ cannot swim but the mutants KJΔRSy and KJΔRSyΔDEF can. This suggests that downregulation of SmeYZ is not sufficient to abolish swimming but total loss of SmeYZ is. Since *smeDEF* inactivation in KJΔRSy did not change this, then either small level of SmeYZ expression can support swimming phenotype, or there is a compensatory mechanism occurring in KJΔRSy (but it is not SmeDEF). The similar phenomenon can be noticed in wild-type KJ and mutants KJΔYZ, KJΔRSy, and KJΔRSyΔDEF with respect to oxidative stress susceptibility (Fig 3B). Nevertheless, compared with wild-type KJ, KJΔDEF decreased the secreted protease activity, but hardly affected oxidative stress susceptibility and swimming (Fig 3), supporting that SmeDEF and SmeYZ have an overlapped physiological function in protease secretion, but not in oxidative stress tolerance and swimming. In an attempt to determine the linkage of SmeDEF upregulation to the compensatory circuit, *ΔsmeDEF* allele was introduced into KJΔRSy and the physiological functions of the resultant mutant KJΔRSyΔDEF were assessed. Inactivation of *smeDEF* conferred KJΔRSy a decreased secreted protease activity (Fig 3C), signifying that upregulation of SmeDEF in KJΔRSy compensates the SmeYZ downexpression-mediated compromise in secreted protease activity.

Microorganism contains a variety of TCSs that regulate complex antibiotic resistance. Based on the regulatory mechanisms of those TCSs already characterized, several regulatory models have been proposed. Firstly, the TCSs definitely regulate the antibiotics resistance determinants (either operons or genes), for instance, AdeABC by the AdeRS system of *Acintobacter baumannii*, CzcCBA by the CzcRS system of *Pseudomonas aeruginosa* and SmeABC by the SmeRS system of *S*. *maltophilia* [9, 25–26]. Secondly, the different response regulators from individually TCS act on the same antibiotics resistance determinants. The PhoP-PhoQ and PmrA-PmrB systems of *P*. *aeruginosa* provide such an example. The phosphorylated response regulators, PhoP and PmrA, can positively upregulate the transcription of *arnBCADTEF* operon and result in the resistance to polymyxin B and cationic antimicrobials peptides [27]. Thirdly, the activated TCSs can simultaneously affect the expression of a variety of targets genes which involved in the different resistance mechanisms. The CzcRS system of *P*. *aeruginosa* regulates the expression of *czcCAB* operon and *oprD* gene [28]. The BaeSR TCS is responsible for the regulation of MdtABC and AcrD [29]. The EvgSA system activates the expression of MdeEF and EmrKY [30–31]. The present results provide another kind of example that a sensor kinase can partner with different response regulators to inversely modulate two RND-type efflux pumps and the outcome can benefit the maintenance of bacterial physiologic functions.

## Supporting Information

S1 Fig Genetic map of six RND-type efflux pumps and their flanking regulatory determinants. (DOCX)

S2 Fig Schematic genomic organization, promoter transcription fusions, and deletion mutants of the *smeT-smeDEF* and *smeSyRy-smeYZ* clusters of *S*. *maltophilia*. (DOCX)

S3 Fig *SmeSy* and *smeRy* form an operon. (DOCX)

S4 Fig The domains and conserved phosphorylated residues analysis of SmeSy and SmeRy. (DOCX)

S1 Table Bacterial strains, plasmids and primers used in this study (DOCX)

S2 Table Transcriptomic analysis of *S*. *maltophilia* wild-type KJ and *smeRySy* mutant KJΔRSy (DOCX)
