# Supplementary material for: Inactivation of SmeSyRy Two-Component Regulatory System Inversely Regulates the Expression of SmeYZ and SmeDEF Efflux Pumps in Stenotrophomonas maltophilia
Source: PLoS One. 2016 Aug 11;11(8):e0160943. doi: 10.1371/journal.pone.0160943 (PMC4981351; doi:10.1371/journal.pone.0160943)
Supplement: S1 Fig — (DOCX) [file pone.0160943.s001.docx]

***smeR smeS smeA smeB smeC***

***smeT smeD smeE smeF ssssssmltsmlt4069 smeC***

***smeI smeJ smeK***

***smeRo smeO smeP***

***smeRv smeU1 smeV smeW smeU2 smeX***

***smeS_y_ smeR_y_ smeY smeZ***

**S1. Fig. Genetic map of six RND-type efflux pumps and their flanking regulatory determinants.** The six RND-type efflux pump genes are depicted as black arrows, two-component regulatory system genes as grey arrows, and transcription regulator genes as crosshatched arrows.
