# Supplementary material for: Inactivation of SmeSyRy Two-Component Regulatory System Inversely Regulates the Expression of SmeYZ and SmeDEF Efflux Pumps in Stenotrophomonas maltophilia
Source: PLoS One. 2016 Aug 11;11(8):e0160943. doi: 10.1371/journal.pone.0160943 (PMC4981351; doi:10.1371/journal.pone.0160943)
Supplement: S2 Fig — (DOCX) [file pone.0160943.s002.docx]

***smeT smeD smeE smeF ssssssmltsmlt4069 smeC***

***smeT smeD smeE smeF ssssssmltsmlt4069 smeC***

***smeT smeD smeE smeF ssssssmltsmlt4069 smeC***

SmeT3-F/R

(454)

SmeD5-F/R

(406)

SmeF3-F/R

(820)

**KJ**

**(A)**

**KJ∆T**

**KJ∆DEF**

**pSmeD_xylE_**

**pSmeT_xylE_**

**(B)**

SmeRy3-F/R

(390)

SmeRy5-F/R

(580)

SmeSy3-F/R

(503)

**pSmeY_xylE_**

**KJ**

***smeS_y_ smeR_y_ smeY smeZ***

**KJ∆RSy**

**KJ∆Ry**

**S2. Fig. Schematic genomic organization, promoter transcription fusions, and deletion mutants of the *smeT-smeDEF* and *smeRySy-smeYZ* clusters of *S. maltophilia*.** The orientation of gene is indicated by the arrow. The solid lines represent the PCR amplicons for the construction of recombinant plasmids. The primers of PCR were labeled above the solid lines and the numbers in the brackets represent the PCR amplicon size (bps). The gray bars indicate the DNA segments for the construction of promoter transcription fusions. The crosshatched arrows represent the *xylE* cassette. The white box indicates the deleted region for each mutant construct. (A) The *smeT-smeDEF* cluster. (B) The *smeRySy-smeYZ* cluster.
