# Supplementary material for: Inactivation of SmeSyRy Two-Component Regulatory System Inversely Regulates the Expression of SmeYZ and SmeDEF Efflux Pumps in Stenotrophomonas maltophilia
Source: PLoS One. 2016 Aug 11;11(8):e0160943. doi: 10.1371/journal.pone.0160943 (PMC4981351; doi:10.1371/journal.pone.0160943)
Supplement: S3 Fig — (DOCX) [file pone.0160943.s003.docx]

**(A)**

***smeRy***

***smeSy***

SmeRy-F/R

SmeSy-F/R

**(B)**

**1 2 3**


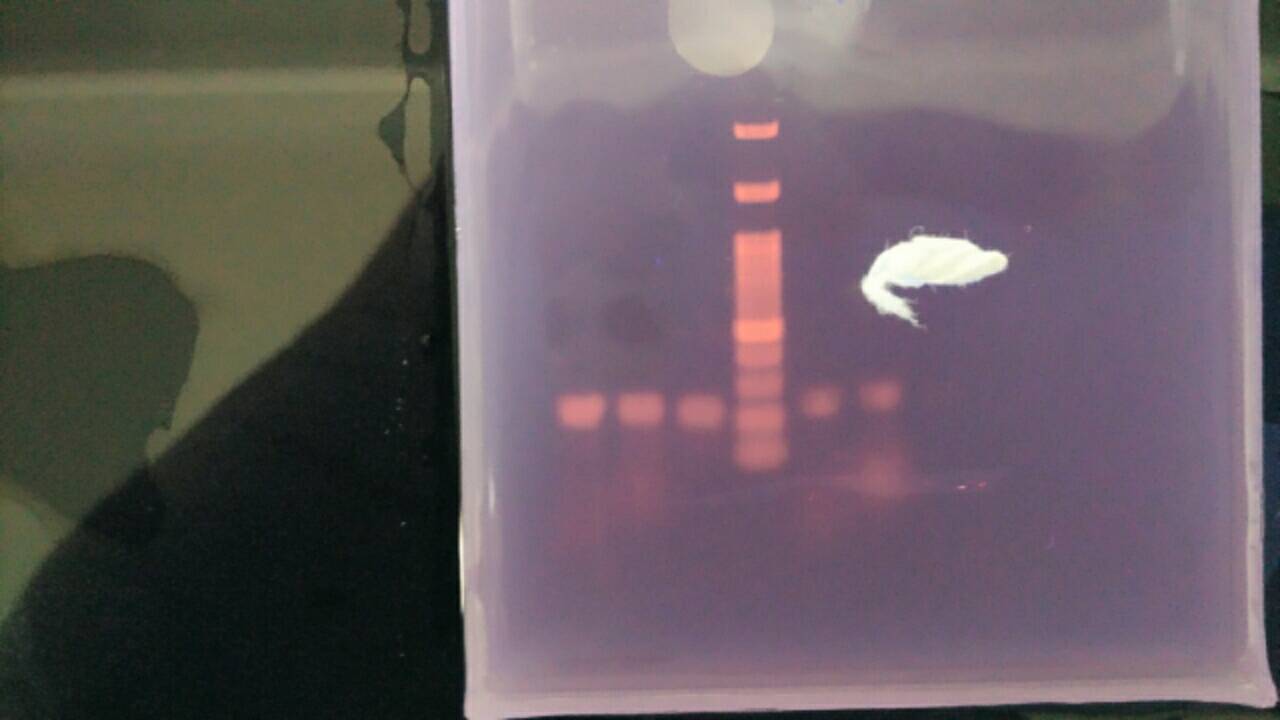


**S3. Fig. *SmeRy* and *smeSy* form an operon**. DNA-free RNA was isolated from logarithmic-phase KJ cells and the cDNAs were obtained by reverse transcriptase-PCR (RT-PCR). The cDNA was used as the template for PCR using the primer sets of SmeRy-F/R and SmeSy-F/R, respectively, and the PCR amplicons were analyzed by agarose gel electrophoresis. (A) Schematic representation of the *smeRy-smeS_Y_* operon and the RT-PCR strategy.  The solid black arrows depict the open reading frames of the operon. The gray arrow represents the primer used to generate the cDNA by RT-PCR. The bars with vertical lines represent the PCR amplicons and the corresponding primer sets are labeled above the bars.  (B) Agarose gel electrophoresis of PCR products. Lane1, the PCR product primered by SmeRy-F and SmeRy-R; Lane2, the PCR product primered by SmeSy-F and SmeSy-R; Lane3, the PCR product primered by SmeX-F and SmeX-R, as a negative control.
