# Supplementary material for: Inactivation of SmeSyRy Two-Component Regulatory System Inversely Regulates the Expression of SmeYZ and SmeDEF Efflux Pumps in Stenotrophomonas maltophilia
Source: PLoS One. 2016 Aug 11;11(8):e0160943. doi: 10.1371/journal.pone.0160943 (PMC4981351; doi:10.1371/journal.pone.0160943)
Supplement: S4 Fig — (DOCX) [file pone.0160943.s004.docx]

**(A)**


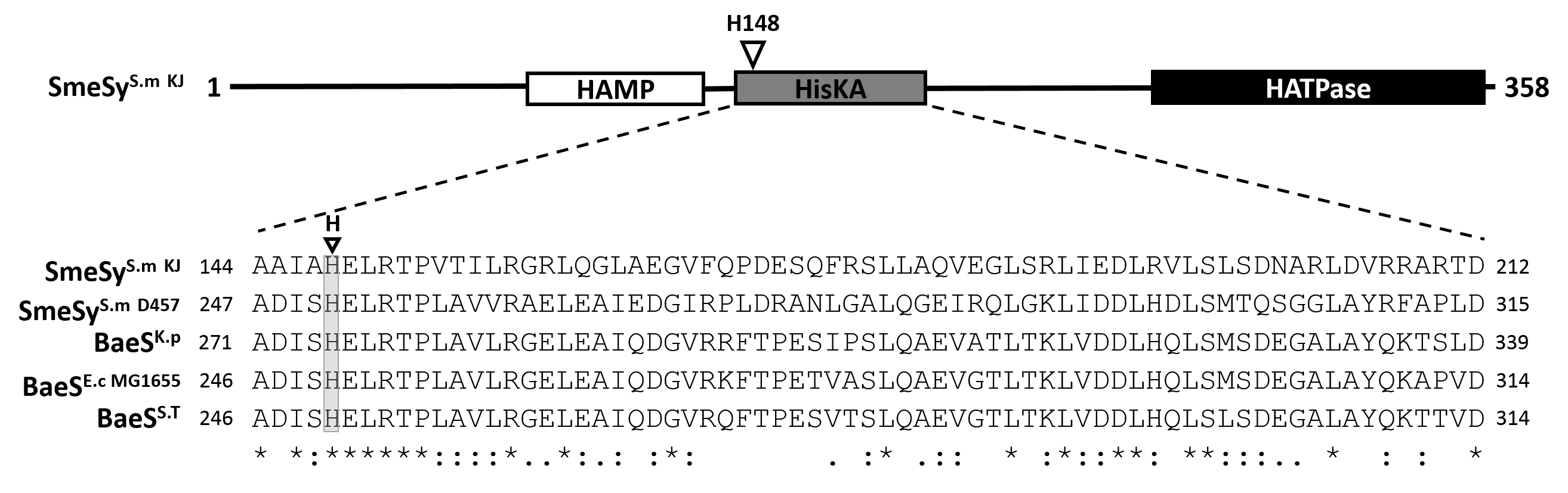


**(B)**

**
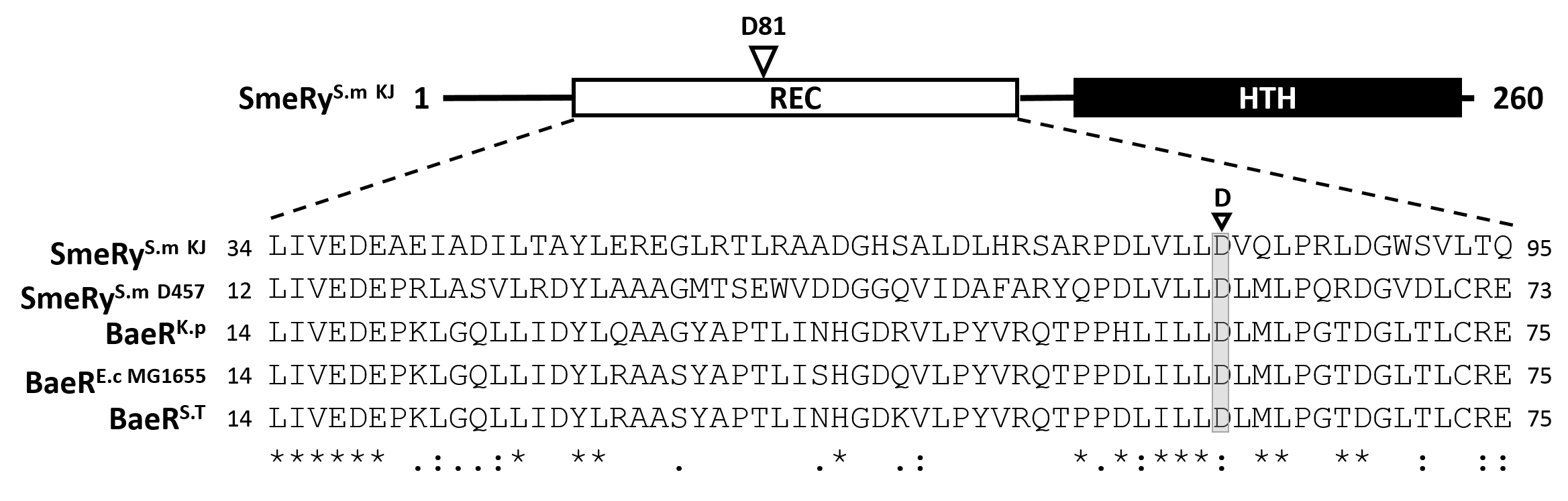
**

**S4. Fig. The domains and conserved phosphorylated residues analysis of SmeSy and SmeRy.** The domain architecture was analyzed by the website of http://www.ncbi.nlm.nih.gov. Multiple sequence alignments among the assayed proteins were performed using the ClustalX program for the identification of conserved phosphorylated residues. SmeSy^S.m KJ^, SmeSy protein of *S. maltophilia* KJ; SmeSy^S.m D457^, SmeSy protein of *S. maltophilia* D457; BaeS^K.p^, BaeS protein of *K. pneumoniae*; BaeS^E.c MG1655^, BaeS protein of *E. coli* MG1655; BaeS^S.T^, BaeS protein of *S.* Typhimurium. (A) Schematic diagram of the domain architecture and the conserved His residue of SmeSy sensor kinase. Inverted triangle depicts the autophosphorylation site (His) of SmeSy. HAMP, histidine kinase, adenylyl cyclase, methyl-accepting protein, and phosphatase (HAMP) domain; HisKA, histidine kinase A domain; HATPase, histidine kinase-like ATPases domain. (B) Schematic diagram of the domain architecture and the conserved Asp residue of SmeRy response regulator. Inverted triangle depicts the phosphorylation site (Asp) of SmeRy. REC, cheY-homologous receiver domain; HTH, helix-turn-helix domain.
