# Supplementary material for: Inactivation of SmeSyRy Two-Component Regulatory System Inversely Regulates the Expression of SmeYZ and SmeDEF Efflux Pumps in Stenotrophomonas maltophilia
Source: PLoS One. 2016 Aug 11;11(8):e0160943. doi: 10.1371/journal.pone.0160943 (PMC4981351; doi:10.1371/journal.pone.0160943)
Supplement: S1 Table — (DOCX) [file pone.0160943.s005.docx]

**S1. Table Bacterial strains, plasmids and primers used in this study**

| **Strain, plasmid, or primer** | **Genotype or properties** |
| --- | --- |
| ***S. maltophilia*** |  |
| KJ | Wild type, a clinical isolate from Taiwan |
| KJΔT | *S. maltophilia* KJ mutant of *smeT* gene; *ΔsmeT* |
| KJΔDEF | *S. maltophilia* KJ mutant of *smeDEF* operon; *ΔsmeDEF* |
| KJΔRSy | *S. maltophilia* KJ mutant of *smeRySy* operon; *ΔsmeRySy* |
| KJΔYZ | *S. maltophilia* KJ mutant of *smeYZ* operon; *ΔsmeYZ* |
| KJΔRSyΔT | *S. maltophilia* KJ double mutant of *smeRySy* operon and *smeT*; *ΔsmeRySy, ΔsmeT* |
| KJΔRy | *S. maltophilia* KJ mutant of *smeRy* gene; *ΔsmeRy* |
| ***Escherichia coli*** |  |
| DH5α | F- φ80d*lacZ*Δ*M15* Δ(*lacZYA-argF*)*U169 deoR recA1 endA1hsdR17* (r_k_^-^ m_k_^+^) *phoA supE44λ^-^thi-1 gyrA96 relA1* |
| S17-1  **Plasmids** | λ pir + mating strain |
| pEX18Tc | *sacB oriT*, Tc^r^ |
| pRK415 | Derived from pRK415, replacing the tetracycline resistance gene with kanamycin resistance gene; Km^r^ |
| pTXylE | Plasmid containing the *xylE* cassette; Amp^r^ |
| pRKXylE | A pRK415-derived vector for the construction of promoter-*xylE* transcription fusion, the orientation of *xylE* gene in this plasmid is opposite to that of *P_lacZ_*of pRK415; Tc^r^ |
| pΔT | pEX18Tc vector with a 888-bp DNA fragment of *S. maltophilia* KJ, containing the *smeT* gene with a internal 135-bp deletion; Tc^r^ |
| pΔDEF | pEX18Tc vector with a 752-bp DNA fragment of *S. maltophilia* KJ, containing the partial N-terminus of *smeD* gene and partial C-terminus of *smeF* gene; Tc^r^ |
| pΔRSy | pEX18Tc vector with a 1068-bp DNA fragment of *S. maltophilia* KJ, containing the *smeRySy* genes with an internal 1001-bp deletion; Tc^r^ |
| pSmeT_xylE_ | pRK415 with a 359-bp DNA fragment containing the upstream region of the *smeT* gene and a *P_smeT_::xylE* transcriptional fusion |
| pSmeD_xylE_ | pRK415 with a 359-bp DNA fragment containing the upstream region of *smeD* gene and a *P_smeD_::xylE* transcriptional fusion |
| pSmeY_xylE_ | pRK415 with a 343-bp DNA fragment containing the upstream region of *smeY* gene and a *P_smeY_::xylE* transcriptional fusion |
| pSmeRy | pRK415 containing an intact *smeRy* gene |
| **Primers**  SmeT3-F  SmeT3-R  SmeD5-F  SmeD5-R  SmeF3-F  SmeF3-R  SmeRy5-F  SmeRy5-R  SmeRy3-F  SmeRy3-R  SmeSy3-F  SmeSy3-R  SmeEQ-F  SmeEQ-R  SmeFQ-F  SmeFQ-R  SmeZQ-F  SmeZQ-R  rDNA-F  rDNA-R | CCAGATCTTCATCGAGCTGTCC  GAGGTACCATACCACGTTGTCC  CCAGATCTTCATCGAGCTGTCC  GAGGTACCATACCACGTTGTCC  AGACTCTAGATGTCAACGAACAGTTCAC  TAGCAAGCTTCGTCCAGGCTGACATTCAAC  AGTACGCTGCAGCCATCCAGCC  TCCGAAGCTTGCTCAAACAGCC  ACGGTCGACAGCCCCGACC  CCAGCGAATTCAACGGCGAC  TGGAGCTCGGCGATGGCCTTGACC  GCGTGCTGCAGCCAGACGAATCCC  TCCTGCCCAACGAAGACC  CTTGAACGAACGCCATGCC  CCCGAGCATCTCGCTGAC  AAGCCCACCTGGATCGAC  TGTCCAGCGTCAAGCACC  GCCGACCAGCATCAGGAAG  GACCTTGCGCGATTGAATG  CGGATCGTCGCCTTGGT |
