# Supplementary material for: Inactivation of SmeSyRy Two-Component Regulatory System Inversely Regulates the Expression of SmeYZ and SmeDEF Efflux Pumps in Stenotrophomonas maltophilia
Source: PLoS One. 2016 Aug 11;11(8):e0160943. doi: 10.1371/journal.pone.0160943 (PMC4981351; doi:10.1371/journal.pone.0160943)
Supplement: S2 Table — (DOCX) [file pone.0160943.s006.docx]

**S2. Table Transcriptomic analysis of *S. maltophilia* wild-type KJ and *smeRySy* mutant KJΔRSy**

| \|  \|  \| \| \| --- \| --- \| --- \| \| **Feature ID** \|  \|  \| | **Transcript abundance** | |
| --- | --- | --- | --- | --- | --- | --- | --- | --- |
|  | **KJ** | **KJΔRSy** |
| \| Smlt0001 (dnaA) \| \| --- \| \| Smlt0002 (dnaN) \| \| Smlt0003 \| \| Smlt0004 (recF) \| \| Smlt0005 (gyrB) \| \| Smlt0006 \| \| Smlt0007 \| \| Smlt0008 \| \| Smlt0009 \| \| Smlt0010 (exbB1) \| \| Smlt0011 (exbD1) \| \| Smlt0012 (exbD2) \| \| Smlt0013 (cls) \| \| Smlt0014 \| \| Smlt0015 \| \| Smlt0016 \| \| Smlt0017 \| \| Smlt0018 \| \| Smlt0019 \| \| Smlt0020 \| \| Smlt0021 \| \| Smlt0023 \| \| Smlt0024 \| \| Smlt0025 \| \| Smlt0026 \| \| Smlt0027 \| \| Smlt0028 \| \| Smlt0029 \| \| Smlt0032 \| \| Smlt0033 \| \| Smlt0034 \| \| Smlt0035 \| \| Smlt0036 (smmL) \| \| Smlt0037 (smmK) \| \| Smlt0038 (smmJ) \| \| Smlt0039 \| \| Smlt0040 \| \| Smlt0041 \| \| Smlt0042 \| \| Smlt0043 \| \| Smlt0044 \| \| Smlt0045 \| \| Smlt0046 \| \| Smlt0047 \| \| Smlt0048 (aspC) \| \| Smlt0049 \| \| Smlt0050 \| \| Smlt0051 \| \| Smlt0052 \| \| Smlt0053 \| \| Smlt0054 \| \| Smlt0056 \| \| Smlt0057 \| \| Smlt0058 \| \| Smlt0059 \| \| Smlt0060 \| \| Smlt0061 \| \| Smlt0062 \| \| Smlt0063 \| \| Smlt0064 \| \| Smlt0065 \| \| Smlt0066 (Q) \| \| Smlt0067 \| \| Smlt0068 \| \| Smlt0069 \| \| Smlt0073 \| \| Smlt0074 \| \| Smlt0075 \| \| Smlt0076 \| \| Smlt0077 (phhA) \| \| Smlt0078 \| \| Smlt0079 \| \| Smlt0080 \| \| Smlt0082 \| \| Smlt0083 \| \| Smlt0084 \| \| Smlt0085 \| \| Smlt0086 \| \| Smlt0087 \| \| Smlt0088 \| \| Smlt0089 \| \| Smlt0090b (fpg) \| \| Smlt0091 (mdoD) \| \| Smlt0092 (tdk) \| \| Smlt0093 \| \| Smlt0094 \| \| Smlt0095 (rep) \| \| Smlt0096 \| \| Smlt0097 \| \| Smlt0098 (hel) \| \| Smtl0099 (pyrF) \| \| Smlt0101 \| \| Smlt0102 (plsB) \| \| Smlt0103 \| \| Smlt0104 (rdgC) \| \| Smlt0105 \| \| Smlt0106 \| \| Smlt0107 \| \| Smlt0108 (gltD) \| \| Smlt0109 (gltB) \| \| Smlt0110 \| \| Smlt0111 \| \| Smlt0112 \| \| Smlt0113 \| \| Smlt0114 \| \| Smlt0115 (ampC) \| \| Smlt0116 \| \| Smlt0117 \| \| Smlt0118 \| \| Smlt0119 \| \| Smlt0120 (lhr) \| \| Smlt0121 \| \| Smlt0122 \| \| Smlt0123 \| \| Smlt0124 \| \| Smlt0125 \| \| Smlt0126 \| \| Smlt0127 \| \| Smlt0128 \| \| Smlt0129 \| \| Smlt0130 \| \| Smlt0131 \| \| Smlt0133 \| \| Smlt0134 (hipA) \| \| Smlt0135 (hipB) \| \| Smlt0136 \| \| Smlt0137 \| \| Smlt0138 \| \| Smlt0139 \| \| Smlt0140 \| \| Smlt0141 \| \| Smlt0142 \| \| Smlt0143 \| \| Smlt0144 \| \| Smlt0145 \| \| Smlt0146 \| \| Smlt0147 \| \| Smlt0148 \| \| Smlt0149 \| \| Smlt0150 \| \| Smlt0151 (glnA) \| \| Smlt0152 (glnB) \| \| Smlt0153 (amtB) \| \| Smlt0154 \| \| Smlt0155 \| \| Smlt0156 \| \| Smlt0157 \| \| Smlt0158 (glnL) \| \| Smlt0519(glnG) \| \| Smlt0160(sodC1) \| \| Smlt0161 (sodC2) \| \| Smlt0162 \| \| Smlt0163 \| \| Smlt0164 (fadI) \| \| Smlt0165 \| \| Smlt0166 \| \| Smlt0167 (hemD) \| \| Smlt0168 \| \| Smlt0169 \| \| Smlt0170 \| \| Smlt0171 (secB) \| \| Smlt0172 (gpsA) \| \| Smlt0173 \| \| Smlt0174 \| \| Smlt0175 \| \| Smlt0176 \| \| Smlt0177 (arsC) \| \| Smlt 0178 (arsR) \| \| Smlt0179 (arsH) \| \| Smlt0180 \| \| Smlt0181 \| \| Smlt0182 \| \| Smlt0183 \| \| Smlt0184 \| \| Smlt0185 \| \| Smlt0186 \| \| Smlt0187 \| \| Smlt0188 \| \| Smlt0189 \| \| Smlt0190 \| \| Smlt0191 \| \| Smlt0192 \| \| Smlt0193 \| \| Smlt0194 \| \| Smlt0195 \| \| Smlt0196 \| \| Smlt0197 \| \| Smlt0198 \| \| Smlt0199 (GCDH) \| \| Smlt0200 \| \| Smlt0201 \| \| Smlt0203 \| \| Smlt0204 \| \| Smlt0205 (fabH) \| \| Smlt0206 (dhaA) \| \| Smlt0207 \| \| Smlt0208 \| \| Smlt0209 \| \| Smlt0210 \| \| Smlt0211 (ddl) \| \| Smlt0212 \| \| Smlt0213 (ubiB) \| \| Smlt0214 \| \| Smlt0215 \| \| Smlt0216 \| \| Smlt0217 \| \| Smlt0218 \| \| Smlt0219 (truC) \| \| Smlt0220 \| \| Smlt0221 \| \| Smlt0222 \| \| Smlt0223 (dcp) \| \| Smlt0224 \| \| Smlt0225 \| \| Smlt0226 \| \| Smlt0227 \| \| Smlt0228 \| \| Smlt0230 \| \| Smlt0231 (mls) \| \| Smlt0232 (aceA) \| \| Smlt0233 \| \| Smlt0234 \| \| Smlt0236 \| \| Smlt0237 \| \| Smlt0238 \| \| Smlt0239 \| \| Smlt0240 \| \| Smlt0241 \| \| Smlt0242 \| \| Smlt0244 \| \| Smlt0245 \| \| Smlt0246 \| \| Smlt0247 (RRM1) \| \| Smlt0248 \| \| Smlt0249 \| \| Smlt0250 \| \| Smlt0251 \| \| Smlt0252 \| \| Smlt0253 \| \| Smlt0254 \| \| Smlt0255 (purU) \| \| Smlt0257 (pncA) \| \| Smlt0258 \| \| Smlt0259 \| \| Smlt0260 \| \| Smlt0261 \| \| Smlt0262 \| \| Smlt0263 \| \| Smlt0264 \| \| Smlt0265 \| \| Smlt0266 \| \| Smlt0267 \| \| Smlt0268 (mmsB) \| \| Smlt0269 (ohr) \| \| Smlt0270 \| \| Smlt0271 \| \| Smlt0272 \| \| Smlt0273 \| \| Smlt0274 \| \| Smlt0275 (dusA) \| \| Smlt0276 \| \| Smlt0277 \| \| Smlt0278 \| \| Smlt0279 \| \| Smlt0280 \| \| Smlt0281 \| \| Smlt0282 (birA) \| \| Smlt0283 \| \| Smlt0284 \| \| Smlt_t11 \| \| Smlt0285 \| \| Smlt0286 \| \| Smlt0287 \| \| Smlt0288 \| \| Smlt0289 \| \| Smlt0290 \| \| Smlt0291 \| \| Smlt0293 \| \| Smlt0294 \| \| Smlt0295 \| \| Smlt0296 (Gp17) \| \| Smlt0297 \| \| Smlt0298 \| \| Smlt0299 (D) \| \| Smlt0300 (GpU) \| \| Smlt0301 (GpT) \| \| Smlt0302 \| \| Smlt0303 (GpE+E') \| \| Smlt0304 (FII) \| \| Smlt0305 (FI) \| \| Smlt0306 (W) \| \| Smlt0307 (V) \| \| Smlt0308 \| \| Smlt0309 \| \| Smlt0310 (I) \| \| Smlt0311 (J) \| \| Smlt0312 (S) \| \| Smlt0313 (R) \| \| Smlt0314 \| \| Smlt0315 (lys) \| \| Smlt0316 \| \| Smlt0317 \| \| Smlt0318 (X) \| \| Smlt0319 (L) \| \| Smlt0320 (M) \| \| Smlt0321 (N) \| \| Smlt0322 (O) \| \| Smlt0323 (P) \| \| Smlt0324 (Q) \| \| Smlt0325 \| \| Smlt0327 \| \| Smlt0328 \| \| Smlt0329 \| \| Smlt0330 \| \| Smlt0331 \| \| Smlt0332 \| \| Smlt0333 \| \| Smlt0334 \| \| Smlt0335 \| \| Smlt0336 \| \| Smlt0337 \| \| Smlt0339 \| \| Smlt0340 \| \| Smlt0341 (arcA) \| \| Smlt0342 (ecnA) \| \| Smlt0343 \| \| Smlt0344 \| \| Smlt0345 \| \| Smlt0346 \| \| Smlt0347 \| \| Smlt0348 \| \| Smlt0349 \| \| Smlt0350 \| \| Smlt0351 \| \| Smlt0352 \| \| Smlt0353 \| \| Smlt0354 \| \| Smlt0355 \| \| Smlt0357 \| \| Smlt0358 \| \| Smlt0359 \| \| Smlt0360 \| \| Smlt0361 \| \| Smlt0362 \| \| Smlt0363 \| \| Smlt0364 \| \| Smlt0365 \| \| Smlt0366 \| \| Smlt0367 \| \| Smlt0368 \| \| Smlt0369 \| \| Smlt0370 \| \| Smlt0371 \| \| Smlt0372 (katA) \| \| Smlt0373 \| \| Smlt0374 \| \| Smlt0375 \| \| Smlt0376 \| \| Smlt0377 \| \| Smlt0378 \| \| Smlt0379 \| \| Smlt0380 \| \| Smlt0381 \| \| Smlt0382 \| \| Smlt0383 \| \| Smlt0384 \| \| Smlt0385 \| \| Smlt0386 \| \| Smlt0387 \| \| Smlt0389 \| \| Smlt0390 \| \| Smlt0391 \| \| Smlt0392 \| \| Smlt0393 \| \| Smlt0394 \| \| Smlt0395 \| \| Smlt0396 \| \| Smlt0397 \| \| Smlt0398 (argS) \| \| Smlt0399 (radC) \| \| Smlt0400 \| \| Smlt0401 (dfp) \| \| Smlt0402 (dut) \| \| Smlt0403 (algC) \| \| Smlt0404 (kdpE) \| \| Smlt0405 (kdpD) \| \| Smlt0406 (kdpC) \| \| Smlt0407 (kdpB) \| \| Smlt0408 (kdpA) \| \| Smlt0410 \| \| Smlt0411 (pyre) \| \| Smlt0412 \| \| Smlt0413 (ampG) \| \| Smlt0414 \| \| Smlt0415 (anmK) \| \| Smlt0416 \| \| Smlt0417 \| \| Smlt_r10 \| \| Smlt_t22 \| \| Smlt_t33 \| \| Smlt_r01 \| \| Smlt_r05 \| \| Smlt_r12 \| \| Smlt_t44 \| \| Smlt_t55 \| \| Smlt_r06 \| \| Smlt_r04 \| \| Smlt0418 \| \| Smlt0419 \| \| Smlt0420 \| \| Smlt0421 (ctpA) \| \| Smlt0422 \| \| Smlt0423 \| \| Smlt0424 \| \| Smlt0425 (nadR) \| \| Smlt0426 (putA) \| \| Smlt0427 \| \| Smlt0428 (ctaC) \| \| Smlt0429 (ctaD) \| \| Smlt0430 \| \| Smlt0431 \| \| Smlt0432 \| \| Smlt0433 \| \| Smlt0434 \| \| Smlt0435 \| \| Smlt0436 \| \| Smlt0436A \| \| Smlt0438 \| \| Smlt0439 \| \| Smlt0440 \| \| Smlt0441 (dnaG) \| \| Smlt0442 \| \| Smlt0443 \| \| Smlt0444 (rpsU) \| \| Smlt0445 (gcp) \| \| Smlt0446 \| \| Smlt0447 (folB) \| \| Smlt0448 \| \| Smlt0449 (bglX) \| \| Smlt0450 \| \| Smlt0451 \| \| Smlt0452 \| \| Smlt0453 \| \| Smlt0454 \| \| Smlt0455 \| \| Smlt0456 \| \| Smlt0458 \| \| Smlt0459 \| \| Smlt0460 \| \| Smlt0461 \| \| Smlt0462 \| \| Smlt0463 \| \| Smlt0465 \| \| Smlt0466 \| \| Smlt0467 \| \| Smlt0468 \| \| Smlt0469 \| \| Smlt0470 \| \| Smlt0471 \| \| Smlt0473 (glnE) \| \| Smlt0476 \| \| Smlt0477 \| \| Smlt0478 \| \| Smlt0479 \| \| Smlt0480 \| \| Smlt0481 \| \| Smlt0482 \| \| Smlt0483 \| \| Smlt0484 \| \| Smlt0485 \| \| Smlt0487 \| \| Smlt0488 \| \| Smlt0490 (aceE) \| \| Smlt0493 \| \| Smlt0494 \| \| Smlt0496 \| \| Smlt0497 \| \| Smlt0498 \| \| Smlt0498A \| \| Smlt0499 \| \| Smlt0500 \| \| Smlt0501 \| \| Smlt0502 \| \| Smlt0503 \| \| Smlt0504 \| \| Smlt0505 \| \| Smlt0506 \| \| Smlt0507 \| \| Smlt0508 \| \| Smlt0509 \| \| Smlt0510 \| \| Smlt0510A \| \| Smlt0510B \| \| Smlt0512 \| \| Smlt0513 \| \| Smlt0514 \| \| Smlt0515 \| \| Smlt0516 \| \| Smlt0517 \| \| Smlt0518 \| \| Smlt0519 \| \| Smlt0520 \| \| Smlt0521 \| \| Smlt0522 \| \| Smlt0523 \| \| Smlt0524 \| \| Smlt0525 \| \| Smlt0526 \| \| Smlt0527 \| \| Smlt0528 \| \| Smlt0529 \| \| Smlt0531 \| \| Smlt0532 \| \| Smlt0533 \| \| Smlt0534 \| \| Smlt0535 \| \| Smlt0537 \| \| Smlt0538 \| \| Smlt0539 \| \| Smlt0540 \| \| Smlt0541 \| \| Smlt0542 \| \| Smlt0543 \| \| Smlt0544 \| \| Smlt0545 \| \| Smlt0546 \| \| Smlt0547 \| \| Smlt0548 \| \| Smlt0549 \| \| Smlt0550 \| \| Smlt0551 \| \| Smlt0552 \| \| Smlt0553 \| \| Smlt0554 \| \| Smlt0555 \| \| Smlt0556 \| \| Smlt0557 \| \| Smlt0558 \| \| Smlt0559 \| \| Smlt0560 \| \| Smlt0561 (motB) \| \| Smlt0562 (motA) \| \| Smlt0563 \| \| Smlt0564 \| \| Smlt00566 (lrp) \| \| Smlt0567 \| \| Smlt00568 (alr) \| \| Smlt0569 \| \| Smlt0570 \| \| Smlt0571 \| \| Smlt0572 \| \| Smlt0573 \| \| Smlt0574 (rsuA) \| \| Smlt0575 \| \| Smlt0576 (fabB) \| \| Smlt0577 (fabA) \| \| Smlt0578 (dinP) \| \| Smlt0580 \| \| Smlt0581 \| \| Smlt0582 \| \| Smlt0583 \| \| Smlt0584 (gph) \| \| Smlt0585 \| \| Smlt0586 \| \| Smlt0587 \| \| Smlt0588 \| \| Smlt0589 (bioD) \| \| Smlt0590 \| \| Smlt0591 \| \| Smlt0592 \| \| Smlt0593 \| \| Smlt0594 \| \| Smlt0595 \| \| Smlt0596  Smlt0597 \| \| Smlt0598 \| \| Smlt0599 \| \| Smlt0600 \| \| Smlt0601 \| \| Smlt0602 \| \| Smlt0603 \| \| Smlt0605 (rhlE1) \| \| Smlt0608 \| \| Smlt0609 \| \| Smlt0610 \| \| Smlt0611 \| \| Smlt0612 (pilU) \| \| Smlt0613 \| \| Smlt0614 \| \| Smlt0615 \| \| Smlt0616 (cbsB) \| \| Smlt0617 (CYS1) \| \| Smlt0618 (wzm) \| \| Smlt0619 (wzt) \| \| Smlt0620 (cat) \| \| Smlt0621 (wxocD) \| \| Smlt0622 (wxocA) \| \| Smlt0623 (wxocBC) \| \| Smlt0624 \| \| Smlt0625 \| \| Smlt0626 \| \| Smlt0627 \| \| Smlt0628 \| \| Smlt0629 \| \| Smlt0630 \| \| Smlt0631 \| \| Smlt0632 \| \| Smlt0633 \| \| Smlt0634 \| \| Smlt0635 \| \| Smlt0636 \| \| Smlt0637 \| \| Smlt0638 \| \| Smlt0639 \| \| Smlt0640 \| \| Smlt0641 (arnC) \| \| Smlt0642 \| \| Smlt0644 \| \| Smlt0645 (etfA) \| \| Smlt0646 (etfB) \| \| Smlt0647 (rfbB) \| \| Smlt0648 (rmlA) \| \| Smlt0649 (rmlC) \| \| Smlt0650 (rfbD) \| \| Smlt0651 \| \| Smlt0652 (manA) \| \| Smlt0653 (manB) \| \| Smlt0654 \| \| Smlt0655 (lpsI) \| \| Smlt0656 (lpsJ) \| \| Smlt0657 (wxocA) \| \| Smlt0658 \| \| Smlt0659 \| \| Smlt0660 \| \| Smlt0661 \| \| Smlt0662 \| \| Smlt0663 \| \| Smlt0664 \| \| Smlt0665 (pros) \| \| Smlt0666 \| \| Smlt0668 \| \| Smlt0669 \| \| Smlt0670 \| \| Smlt0671 (rimI) \| \| Smlt0672 \| \| Smlt0673 (valS) \| \| Smlt0674 \| \| Smlt0675 (pepA) \| \| Smlt0676 \| \| Smlt0677 \| \| Smlt0678 \| \| Smlt0679 \| \| Smlt0680 (xerD) \| \| Smlt0681 (dsbC) \| \| Smlt0682 (chiA) \| \| Smlt0684 (purl) \| \| Smlt0685 \| \| Smlt0686 \| \| Smlt0687 (xpsE) \| \| Smlt0688 (xpsF) \| \| Smlt0689 (xpsG) \| \| Smlt0690 (xpsH) \| \| Smlt0691 (xpsI) \| \| Smlt0692 (xpsJ) \| \| Smlt0693 (pefK) \| \| Smlt0694 (pefL) \| \| Smlt0695 (xpsM) \| \| Smlt0696 (xpsN) \| \| Smlt0697 (xpsD) \| \| Smlt0698 \| \| Smlt0700 \| \| Smlt0701 \| \| Smlt0702 \| \| Smlt0703 (pncB) \| \| Smlt0704 \| \| Smlt0706 (smf-1) \| \| Smlt0707 \| \| Smlt0708 (mrkC) \| \| Smlt0709 \| \| Smlt0710 (wecB) \| \| Smlt0711 \| \| Smlt0712 (nfrB) \| \| Smlt0713 \| \| Smlt0714 \| \| Smlt0715 \| \| Smlt0716 \| \| Smlt0717 \| \| Smlt0718 (glyA) \| \| Smlt0719 \| \| Smlt0720 \| \| Smlt0721 \| \| Smlt0722 (nrdR) \| \| Smlt0723 \| \| Smlt0724 \| \| Smlt0725 \| \| Smlt0726 \| \| Smlt0727 (ribE) \| \| Smlt0728 (rib) \| \| Smlt0729 (ribH) \| \| Smlt0730 (nusB) \| \| Smlt0731 \| \| Smlt0732 \| \| Smlt0733 \| \| Smlt0734 \| \| Smlt0735 \| \| Smlt0736 \| \| Smlt0737 \| \| Smlt0738 (glcD) \| \| Smlt0739 \| \| Smlt0741 \| \| Smlt0742 \| \| Smlt0743 \| \| Smlt0745 \| \| Smlt0746 \| \| Smlt0747 \| \| Smlt0748 (mraW) \| \| Smlt0749 (ftsL) \| \| Smlt0750 (ftsI) \| \| Smlt0751 (murE) \| \| Smlt0752 (murF) \| \| Smlt0753 (mraY) \| \| Smlt0754 (ftsW) \| \| Smlt0755 (murG) \| \| Smlt0756 (murC) \| \| Smlt0757 (ddl) \| \| Smlt0758 (ftsQ) \| \| Smlt0759 (ftsA) \| \| Smlt0760 (ftsZ) \| \| Smlt0761 (lpxC) \| \| Smlt0762 \| \| Smlt0763 \| \| Smlt0764 (secA) \| \| Smlt0765 \| \| Smlt0766 \| \| Smlt0767 (metF) \| \| Smlt0768 \| \| Smlt0769 \| \| Smlt0770 \| \| Smlt0771 \| \| Smlt0772 \| \| Smlt0773 \| \| Smlt0774 \| \| Smlt0775 (sahH) \| \| Smlt0777 \| \| Smlt0778 (ppc) \| \| Smlt0779 \| \| Smlt0780 (metK) \| \| Smlt0781 \| \| Smlt0782 \| \| Smlt0783 \| \| Smlt0784 \| \| Smlt0785 \| \| Smlt0786 \| \| Smlt0787 (dusB) \| \| Smlt0788 \| \| Smlt0789 \| \| Smlt0790 \| \| Smlt0791 (rbsK) \| \| Smlt0792 \| \| Smlt0793 \| \| Smlt0794 \| \| Smlt0795 (huvA) \| \| Smlt0796 \| \| Smlt0797 \| \| Smlt0798 \| \| Smlt0799 \| \| Smlt0800 \| \| Smlt0801 \| \| Smlt0802 \| \| Smlt0803 \| \| Smlt0804 (dgkA) \| \| Smlt0805 \| \| Smlt0806 \| \| Smlt0807 \| \| Smlt0808 \| \| Smlt0809 (lemA) \| \| Smlt0810 \| \| Smlt0811 \| \| Smlt0812 (lgt) \| \| Smlt0813 (thyA) \| \| Smlt0814 (folA) \| \| Smlt0815 \| \| Smlt0816 (apaH) \| \| Smlt0817 (apaG) \| \| Smlt0818 (ksgA) \| \| Smlt0819 (pdxA) \| \| Smlt0820 (surA) \| \| Smlt0821 (ostA) \| \| Smlt0822 \| \| Smlt0823 \| \| Smlt0824 \| \| Smlt0825 (ubiH) \| \| Smlt0826 (ubiF) \| \| Smlt0827 \| \| Smlt0828 \| \| Smlt0829 \| \| Smlt0830 \| \| Smlt0831 \| \| Smlt0832 (glnS) \| \| Smlt0833 \| \| Smlt0834 (msrA) \| \| Smlt0835 \| \| Smlt0836 \| \| Smlt0837 (talB) \| \| Smlt0838 (rnk) \| \| Smlt0839 (oxyR) \| \| Smlt0840 (ahpF) \| \| Smlt0841 (ahpC) \| \| Smlt0842 (hemK) \| \| Smlt0843 (pip) \| \| Smlt0844 \| \| Smlt0845 \| \| Smlt0846 \| \| Smlt0847 (nudF) \| \| Smlt0848 \| \| Smlt0849 \| \| Smlt0851 \| \| Smlt0852 (pntAa) \| \| Smlt0853 \| \| Smlt0854 \| \| Smlt0855 \| \| Smlt0856 (pntAb) \| \| Smlt0857 (pntB) \| \| Smlt0858 \| \| Smlt0859 \| \| Smlt0860 (ilvE) \| \| Smlt0861 (StmPr2) \| \| Smlt0862 \| \| Smlt0863 \| \| Smlt0864 \| \| Smlt0865 (rbn) \| \| Smlt0866 \| \| Smlt0868 \| \| Smlt0869 \| \| Smlt0870 (prfA) \| \| Smlt0871 (hemA) \| \| Smlt0872 \| \| Smlt0873 (lolB) \| \| Smlt0874 (ipk) \| \| Smlt_t66 \| \| Smlt0875 (prs) \| \| Smlt0876 (rplY) \| \| Smlt0877 (pth) \| \| Smlt0878 \| \| Smlt0880 \| \| Smlt0881 \| \| Smlt0882 \| \| Smlt0883 \| \| Smlt0884 \| \| Smlt0885 \| \| Smlt0886 \| \| Smlt0887 \| \| Smlt_t72 \| \| Smlt_t73 \| \| Smlt_t74 \| \| Smlt0890 (tufB) \| \| Smlt_t01 \| \| Smlt0891 (secE) \| \| Smlt0892 (nusG) \| \| Smlt0894 (rplK) \| \| Smlt0895 (rplA) \| \| Smlt0896 (rplJ) \| \| Smlt0897 (rplL) \| \| Smlt0898 (rpoB) \| \| Smlt0899 (rpoC) \| \| Smlt0901 (rpsL) \| \| Smlt0902 (rpsG) \| \| Smlt0903 (fusA) \| \| Smlt0904 (tuf) \| \| Smlt0905 (rpsJ) \| \| Smlt0906 (rplC) \| \| Smlt0907 (rplD) \| \| Smlt0908 (rplW) \| \| Smlt0909 (rplB) \| \| Smlt0910 (rpsS) \| \| Smlt0911 (rplV) \| \| Smlt0912 (rpsC) \| \| Smlt0913 (rplP) \| \| Smlt0914 (rpmC) \| \| Smlt0915 (rpsQ) \| \| Smlt0916 (rplN) \| \| Smlt0917 (rplX) \| \| Smlt0918 (rplE) \| \| Smlt0919 (rpsN) \| \| Smlt0920 (rpsH) \| \| Smlt0921 (rplF) \| \| Smlt0922 (rplR) \| \| Smlt0923 (rpsE) \| \| Smlt0924 (rpmD) \| \| Smlt0925 (rplO) \| \| Smlt0926 (secY) \| \| Smlt0928 (rpsM) \| \| Smlt0929 (rpsK) \| \| Smlt0930 (rpsD) \| \| Smlt0931 (rpoA) \| \| Smlt0933 (rplQ) \| \| Smlt0935 \| \| Smlt0936 \| \| Smlt0937 (aroH) \| \| Smlt0939 \| \| Smlt0940 \| \| Smlt0941 \| \| Smlt0942 (typA) \| \| Smlt0943 (ppi) \| \| Smlt0944(mdh) \| \| Smlt0945 (rluA) \| \| Smlt0947 (prpE) \| \| Smlt0948 \| \| Smlt0949 \| \| Smlt0950 (gst) \| \| Smlt0951 \| \| Smlt0952 (fadH) \| \| Smlt0953 \| \| Smlt0954 \| \| Smlt0955 \| \| Smlt0956 \| \| Smlt0957 \| \| Smlt0958 \| \| Smlt0959 (kbl) \| \| Smlt0960 \| \| Smlt0961 (tdh) \| \| Smlt0962 \| \| Smlt0963 \| \| Smlt0964 (folC) \| \| Smlt0965 \| \| Smlt0966 \| \| Smlt0967 (purF) \| \| Smlt0968 \| \| Smlt0969 \| \| Smlt0970 \| \| Smlt0971 (lpxH) \| \| Smlt0972 \| \| Smlt0973 (pimB) \| \| Smlt0974 \| \| Smlt0975 (ppx) \| \| Smlt0976 (ppk) \| \| Smlt0977 (phoR) \| \| Smlt0978 (phoB) \| \| Smlt0979 \| \| Smlt0980 (grxC) \| \| Smlt0981 \| \| Smlt0982 \| \| Smlt0983 \| \| Smlt0984 \| \| Smlt0985 \| \| Smlt0986 \| \| Smlt_t02 \| \| Smlt_t03 \| \| Smlt_t04 \| \| Smlt_t05 \| \| Smlt_t06 \| \| Smlt0987 \| \| Smlt_t07 \| \| Smlt0988 (tig) \| \| Smlt0989 (clpP) \| \| Smlt0990 (clpX) \| \| Smlt0991 (lon) \| \| Smlt0992 (hupB) \| \| Smlt_t08 \| \| Smlt_t09 \| \| Smlt_t10 \| \| Smlt_t12 \| \| Smlt_t13 \| \| Smlt0993 \| \| Smlt0994 \| \| Smlt0995 \| \| Smlt0996 \| \| Smlt0997 (rnhA) \| \| Smlt0998 (dnaQ) \| \| Smlt0999 \| \| Smlt_t14 \| \| Smlt1001 \| \| Smlt1002 \| \| Smlt1003 (waaF) \| \| Smlt1004 \| \| Smlt1005 \| \| Smlt1006 \| \| Smlt1007 \| \| Smlt1009 \| \| Smlt1010 \| \| Smlt1011 \| \| Smlt1012 \| \| Smlt1013 \| \| Smlt_t15 \| \| Smlt1014 (dnaX) \| \| Smlt1015 \| \| Smlt1016 (recR) \| \| Smlt1017 \| \| Smlt1018 \| \| Smlt1019 \| \| Smlt1020 \| \| Smlt1021 \| \| Smlt1022 \| \| Smlt1023 \| \| Smlt1024 \| \| Smlt1025 (rpmF) \| \| Smlt1026 (fabH) \| \| Smlt1027 \| \| Smlt1028 (fabD) \| \| Smlt1029 (fabG) \| \| Smlt1030 (acpP) \| \| Smlt1031 (fabF) \| \| Smlt1032 \| \| Smlt1034 \| \| Smlt1035 \| \| Smlt1036 (holB) \| \| Smlt1037 \| \| Smlt_t16 \| \| Smlt1038 \| \| Smlt1039 \| \| Smlt1040 \| \| Smlt1041 \| \| Smlt1042 \| \| Smlt1043 \| \| Smlt1044 \| \| Smlt1045 \| \| Smlt1046 \| \| Smlt1047 \| \| Smlt1048 \| \| Smlt1049 \| \| Smlt1050 (gpW) \| \| Smlt1051 (V) \| \| Smlt1052 \| \| Smlt1053 \| \| Smlt1054 \| \| Smlt1055 \| \| Smlt1056 \| \| Smlt1057 \| \| Smlt1058 \| \| Smlt1059 \| \| Smlt1060 \| \| Smlt1061 \| \| Smlt1064 \| \| Smlt1065 \| \| Smlt1066 \| \| Smlt1067 \| \| Smlt1068 \| \| Smlt1069 \| \| Smlt1070 \| \| Smlt1071 (qnrB) \| \| Smlt1072 \| \| Smlt1073 \| \| Smlt1074 \| \| Smlt1075 \| \| Smlt1076 \| \| Smlt1077 \| \| Smlt1078 \| \| Smlt1080 \| \| Smlt1081 \| \| Smlt1082 \| \| Smlt1083 \| \| Smlt1084 (soxR) \| \| Smlt1085 \| \| Smlt1086 \| \| Smlt1087 \| \| Smlt1088 \| \| Smlt1089 (pilT) \| \| Smlt1090 (pilU) \| \| Smlt1091 \| \| Smlt1092 \| \| Smlt1093 \| \| Smlt1094 \| \| Smlt1095 \| \| Smlt1096 (tag) \| \| Smlt1097 (yhgE) \| \| Smlt1098 \| \| Smlt1099 \| \| Smlt1100 (pyrB) \| \| Smlt1101 \| \| Smlt1102 \| \| Smlt1103 \| \| Smlt1104 (ptsI) \| \| Smlt1105 (ptsH) \| \| Smlt1106 \| \| Smlt1108 \| \| Smlt1109 \| \| Smlt1110 (rpoP) \| \| Smlt1111 \| \| Smlt1112 (rpoN) \| \| Smlt1113 \| \| Smlt1114 \| \| Smlt1115 \| \| Smlt1116 \| \| Smlt1117 (kdsD) \| \| Smlt1118 \| \| Smlt1119 (murA) \| \| Smlt1120 \| \| Smlt1121 \| \| Smlt1122 \| \| Smlt1123 (purN) \| \| Smlt1124 \| \| Smlt1125 \| \| Smlt1126 (purM) \| \| Smlt1127 \| \| Smlt1128 \| \| Smlt1129 \| \| Smlt1130 \| \| Smlt1131 \| \| Smlt1132 \| \| Smlt1133 \| \| Smlt1134 \| \| Smlt1135 \| \| Smlt1136 \| \| Smlt1137 \| \| Smlt1138 \| \| Smlt1139 (apbE) \| \| Smlt1140 \| \| Smlt1141 \| \| Smlt1142 \| \| Smlt1144c(bfrA) \| \| Smlt1145 \| \| Smlt1146 \| \| Smlt1147 \| \| Smlt1148 \| \| Smlt1149 \| \| Smlt1150 \| \| Smlt1151 \| \| Smlt1152 \| \| Smlt1153 \| \| Smlt1154 \| \| Smlt1155 \| \| Smlt1156 \| \| Smlt1157 \| \| Smlt1158 \| \| Smlt1159 \| \| Smlt1160 \| \| Smlt1161 \| \| Smlt1162 \| \| Smlt1163 \| \| Smlt1164 \| \| Smlt1165 (osmC) \| \| Smlt1166 (pab) \| \| Smlt1167 \| \| Smlt1168 \| \| Smlt1169  Smlt1170 (murD) \| \| Smlt1171 \| \| Smlt1172 \| \| Smlt1173 (ispB) \| \| Smlt1174 (aglA) \| \| Smlt1175 \| \| Smlt1176 \| \| Smlt1177 \| \| Smlt1178 \| \| Smlt1179 (amyM) \| \| Smlt1180 \| \| Smlt1181 \| \| Smlt1182 \| \| Smlt1183 \| \| Smlt1184 \| \| Smlt1185 \| \| Smlt1186 \| \| Smlt1187 (ssb) \| \| Smlt1188 \| \| Smlt1189 \| \| Smlt1190 \| \| Smlt1191 \| \| Smlt1192 \| \| Smlt1193 \| \| Smlt1194 \| \| Smlt1195 \| \| Smlt1196 \| \| Smlt1197 \| \| Smlt1198 (adrA) \| \| Smlt1199 \| \| Smlt1200 \| \| Smlt1201 \| \| Smlt1202 \| \| Smlt1203 \| \| Smlt1204 \| \| Smlt1205 \| \| Smlt1206 \| \| Smlt1207 \| \| Smlt1208 \| \| Smlt1209 \| \| Smlt1210 \| \| Smlt1211 \| \| Smlt1212 \| \| Smlt1213 \| \| Smlt1214 \| \| Smlt1215 \| \| Smlt1216 \| \| Smlt1217 \| \| Smlt1218 \| \| Smlt1219 \| \| Smlt1220 \| \| Smlt1221 \| \| Smlt1222 \| \| Smlt1223 \| \| Smlt1224 \| \| Smlt1225 \| \| Smlt1226 \| \| Smlt1227 \| \| Smlt1228 \| \| Smlt1229 \| \| Smlt1230 \| \| Smlt1231 \| \| Smlt1232 \| \| Smlt1233 \| \| Smlt1234 (mqo) \| \| Smlt1235 \| \| Smlt1236 \| \| Smlt1237 \| \| Smlt1238 \| \| Smlt1240 \| \| Smlt1241 \| \| Smlt1242 \| \| Smlt1243 \| \| Smlt1245 \| \| Smlt1246 \| \| Smlt1247 \| \| Smlt1249 \| \| Smlt1250 (minE) \| \| Smlt1251 (mind) \| \| Smlt1252 (minC) \| \| Smlt1253 \| \| Smlt1254 \| \| Smlt1255 \| \| Smlt1256 \| \| Smlt1257 \| \| Smlt1259 \| \| Smlt1260 \| \| Smlt1261 \| \| Smlt1262 \| \| Smlt1263 \| \| Smlt1264 (purT) \| \| Smlt1265 \| \| Smlt1266 \| \| Smlt1268 \| \| Smlt1269 (rfaY) \| \| Smlt1270 \| \| Smlt1271 (tesB) \| \| Smlt1273 \| \| Smlt1274 \| \| Smlt1275 \| \| Smlt1276 \| \| Smlt1277 (uvrA) \| \| Smlt1278 (rplU) \| \| Smlt1279 (rpmA) \| \| Smlt1280(obgE) \| \| Smlt1282 \| \| Smlt1283 (trbI) \| \| Smlt1284 (trbG) \| \| Smlt1285 (trbF) \| \| Smlt1286 (trbL) \| \| Smlt1287 (trbJ) \| \| Smlt1288 (trbE) \| \| Smlt1289 (trbD) \| \| Smlt1290 (trbC) \| \| Smlt1291 (trbB) \| \| Smlt1292 \| \| Smlt1293 (traG) \| \| Smlt1294 \| \| Smlt1295 \| \| Smlt1296 \| \| Smlt1297 \| \| Smlt1298 \| \| Smlt1299 \| \| Smlt1300 \| \| Smlt1301 \| \| Smlt1302 \| \| Smlt1303 \| \| Smlt1304 \| \| Smlt1305 \| \| Smlt1306 \| \| Smlt1307 \| \| Smlt1308 \| \| Smlt1309 \| \| Smlt1310 \| \| Smlt1311 \| \| Smlt1312 \| \| Smlt1313 \| \| Smlt1314 \| \| Smlt1315 \| \| Smlt1316 \| \| Smlt1318 \| \| Smlt1319 \| \| Smlt1320 \| \| Smlt1320A \| \| Smlt1322 \| \| Smlt1323 \| \| Smlt1324 \| \| Smlt1325 \| \| Smlt1326 \| \| Smlt1327 \| \| Smlt1328 \| \| Smlt1329 \| \| Smlt1331 \| \| Smlt1332 \| \| Smlt1333 \| \| Smlt1334 \| \| Smlt1335 \| \| Smlt1336 \| \| Smlt1337 (rpsT) \| \| Smlt1338 (mviN) \| \| Smlt1339 (ribF) \| \| Smlt1340 (ileS) \| \| Smlt1341 (lspA) \| \| Smlt1342 (ispH) \| \| Smlt1343 \| \| Smlt1344 \| \| Smlt1345 \| \| Smlt1346 \| \| Smlt1347 \| \| Smlt1348 \| \| Smlt1349 \| \| Smlt1350 \| \| Smlt1351 (ldhA) \| \| Smlt_t17 \| \| Smlt1353 \| \| Smlt1354 \| \| Smlt1355 \| \| Smlt1356 \| \| Smlt1357 \| \| Smlt1358 \| \| Smlt1359 \| \| Smlt1360(cyoA) \| \| Smlt1361(qoxB) \| \| Smlt1362(qoxC) \| \| Smlt1363(cyoxD) \| \| Smlt1365 \| \| Smlt1366 (radA) \| \| Smlt1367 (htpG) \| \| Smlt1368 \| \| Smlt1369 \| \| Smlt1370 \| \| Smlt1371 (ffh) \| \| Smlt1372 \| \| Smlt1373 \| \| Smlt1374 (rpsP) \| \| Smlt1375 (rim) \| \| Smlt1376 (trmD) \| \| Smlt1377 (rplS) \| \| Smlt1378 \| \| Smlt1379 \| \| Smlt1380 \| \| Smlt1381 \| \| Smlt1383 \| \| Smlt1384 \| \| Smlt1385 (katA) \| \| Smlt1386 \| \| Smlt1387 (mutS) \| \| Smlt1388 \| \| Smlt1389 \| \| Smlt1390 \| \| Smlt1391 \| \| Smlt1395 \| \| Smlt1396A \| \| Smlt1399 \| \| Smlt1400 \| \| Smlt1401 \| \| Smlt1402 \| \| Smlt1403 \| \| Smlt1404 \| \| Smlt1405 \| \| Smlt1406 \| \| Smlt1407 \| \| Smlt1408 \| \| Smlt1409 \| \| Smlt1410 (map) \| \| Smlt1411 \| \| Smlt1412 \| \| Smlt1413 \| \| Smlt1414 \| \| Smlt1415 (dat) \| \| Smlt1416 \| \| Smlt1417 \| \| Smlt1418 \| \| Smlt1419 \| \| Smlt1420 \| \| Smlt1421 (qseC) \| \| Smlt1422 \| \| Smlt1423 \| \| Smlt1424 \| \| Smlt1425 \| \| Smlt1426 \| \| Smlt1427 \| \| Smlt1428 \| \| Smlt1429 (nfi) \| \| Smlt1430 (gpmA) \| \| Smlt1431 \| \| Smlt1432 \| \| Smlt1433 (mfd) \| \| Smlt1434 \| \| Smlt1435 \| \| Smlt1436 \| \| Smlt1437 \| \| Smlt1438 \| \| Smlt1439 \| \| Smlt1440 \| \| Smlt1441 (gltX) \| \| Smlt1442 (fur) \| \| Smlt1443 \| \| Smlt1444 \| \| Smlt1445 \| \| Smlt1446 \| \| Smlt1447 \| \| Smlt1448 \| \| Smlt1449 \| \| Smlt1450 \| \| Smlt1451 (ogt) \| \| Smlt1452 \| \| Smlt1453 \| \| Smlt1454 \| \| Smlt1455 \| \| Smlt1456 \| \| Smlt1458 \| \| Smlt1459 \| \| Smlt1460 \| \| Smlt1461 (deaD) \| \| Smlt1462 \| \| Smlt1463 \| \| Smlt1464 \| \| Smlt1465 \| \| Smlt1466 \| \| Smlt1467 \| \| Smlt1468 \| \| Smlt1469 \| \| Smlt1470 \| \| Smlt1471 \| \| Smlt1472 \| \| Smlt1473 (algL) \| \| Smlt1474 \| \| Smlt1475 \| \| Smlt1476 \| \| Smlt1477 \| \| Smlt1478 \| \| Smlt1479 \| \| Smlt1480 \| \| Smlt1481 \| \| Smlt1483 \| \| Smlt1484 \| \| Smlt1485 \| \| Smlt1486 \| \| Smlt1487 \| \| Smlt1488 \| \| Smlt1490(accA) \| \| Smlt1491 \| \| Smlt1492 (dnaE) \| \| Smlt1493 (rnhB) \| \| Smlt1494 (lpxB) \| \| Smlt1495 (lpxA) \| \| Smlt1496 (fabZ) \| \| Smlt1497 (lpxD) \| \| Smlt1498 \| \| Smlt1499 \| \| Smlt1500 (dxr) \| \| Smlt1501 (cdsA) \| \| Smlt1502 (uppS) \| \| Smlt1503 (frr) \| \| Smlt1504 (pyrH) \| \| Smlt1505 \| \| Smlt1506 (tsf) \| \| Smlt1507 (rpsB) \| \| Smlt1508 \| \| Smlt1509 \| \| Smlt1510 \| \| Smlt1511 \| \| Smlt1512 \| \| Smlt1513 \| \| Smlt1514 (map) \| \| Smlt1515 (glnD) \| \| Smlt1516 (dapD) \| \| Smlt1517 \| \| Smlt1518(dapE) \| \| Smlt1520(asnB) \| \| Smlt1521 \| \| Smlt1522 \| \| Smlt1524(bfrA) \| \| Smlt1525(parC) \| \| Smlt1526 \| \| Smlt1527 \| \| Smlt1528 \| \| Smlt1529(emrA) \| \| Smlt1530(emrB) \| \| Smlt1530A \| \| Smlt_t18 \| \| Smlt1532 \| \| Smlt1533 \| \| Smlt_t19 \| \| Smlt_t20 \| \| Smlt1534 (rnr) \| \| Smlt1535 \| \| Smlt1536 \| \| Smlt1537 \| \| Smlt1538 (macB) \| \| Smlt1539 \| \| Smlt1540 \| \| Smlt1541 \| \| Smlt1542 \| \| Smlt1543 (rnt) \| \| Smlt1544 \| \| Smlt1545 \| \| Smlt1546 \| \| Smlt1547 \| \| Smlt1548 \| \| Smlt1549(pstB) \| \| Smlt1550(pstA) \| \| Smlt1551(pstC) \| \| Smlt1552(pstS) \| \| Smlt1554(pstS) \| \| Smlt1555 \| \| Smlt1556 (nth) \| \| Smlt1557 \| \| Smlt1558 (crt2) \| \| Smlt1559 \| \| Smlt1560 \| \| Smlt1561 \| \| Smlt1562 \| \| Smlt1563 \| \| Smlt1564 (rlmL) \| \| Smlt1565 \| \| Smlt1566 \| \| Smlt1567 \| \| Smlt1568 \| \| Smlt1569 \| \| Smlt1570 (aptA) \| \| Smlt1571 (aldH) \| \| Smlt1572 \| \| Smlt1573 \| \| Smlt1574 \| \| Smlt1575 \| \| Smlt1576 \| \| Smlt1577 \| \| Smlt1578 \| \| Smlt1579 \| \| Smlt1580 \| \| Smlt1581 (potF) \| \| Smlt1582 \| \| Smlt1584(potG) \| \| Smlt1585(potH) \| \| Smlt1586 (potI) \| \| Smlt1587 \| \| Smlt1589(corA) \| \| Smlt1590 \| \| Smlt1591 \| \| Smlt1592 \| \| Smlt1593 \| \| Smlt1594 \| \| Smlt1595 \| \| Smlt1596 \| \| Smlt1597 \| \| Smlt1598 \| \| Smlt1599 \| \| Smlt1600 \| \| Smlt1601 (gstA) \| \| Smlt1602 \| \| Smlt1603 \| \| Smlt1604 (petA) \| \| Smlt1605 (petB) \| \| Smlt1606 (petC) \| \| Smlt1607 (sspA) \| \| Smlt1608 (sspB) \| \| Smlt1609 \| \| Smlt1610 \| \| Smlt1611 (nadC) \| \| Smlt1612 \| \| Smlt1613 (purE) \| \| Smlt1614 (purK) \| \| Smlt1615 \| \| Smlt1616 (sodB) \| \| Smlt1617 \| \| Smlt1618 \| \| Smlt1619 \| \| Smlt1620 \| \| Smlt1621 (fimU) \| \| Smlt1622 (pilV) \| \| Smlt1623 (pilW) \| \| Smlt1624 (pilX) \| \| Smlt1625 (pilY1) \| \| Smlt1626 (pilE) \| \| Smlt_t21 \| \| Smlt1627 (fimT) \| \| Smlt1628 (uvrB) \| \| Smlt_t23 \| \| Smlt_t24 \| \| Smlt1629 \| \| Smlt1630 \| \| Smlt1631 \| \| Smlt1632 \| \| Smlt1633 \| \| Smlt1634 \| \| Smlt1635 \| \| Smlt1636 \| \| Smlt1637 \| \| Smlt1638 \| \| Smlt1639 \| \| Smlt1640 (msbA) \| \| Smlt1641 (lpxK) \| \| Smlt1642 (kdsB) \| \| Smlt1643 \| \| Smlt1644 \| \| Smlt1645(uvrC) \| \| Smlt1646(pgsA) \| \| Smlt_t25 \| \| Smlt1648 \| \| Smlt_t26 \| \| Smlt1651 \| \| Smlt1652 \| \| Smlt1653 \| \| Smlt1654 \| \| Smlt1655 \| \| Smlt1656 \| \| Smlt_t27 \| \| Smlt_t28 \| \| Smlt_t29 \| \| Smlt1657 \| \| Smlt1658 \| \| Smlt1659 \| \| Smlt1660 \| \| Smlt1661 \| \| Smlt1662 \| \| Smlt1663 \| \| Smlt1664 \| \| Smlt1665 \| \| Smlt1666 \| \| Smlt1667 \| \| Smlt1668 \| \| Smlt1669 \| \| Smlt1670 \| \| Smlt1671 \| \| Smlt1672 \| \| Smlt1674 \| \| Smlt1675 \| \| Smlt1677 \| \| Smlt1678 \| \| Smlt1679 \| \| Smlt1680 \| \| Smlt1681 \| \| Smlt1682 \| \| Smlt1683 \| \| Smlt1684 \| \| Smlt1685 \| \| Smlt1686 \| \| Smlt1687 \| \| Smlt1688 \| \| Smlt1689 \| \| Smlt1690 (hemN) \| \| Smlt_t30 \| \| Smlt1691 \| \| Smlt1692 (wbpO) \| \| Smlt1693 \| \| Smlt1694 \| \| Smlt1695 \| \| Smlt1696 (fkbP) \| \| Smlt1697 \| \| Smlt1699 \| \| Smlt1700 \| \| Smlt1701 \| \| Smlt1703(exoD) \| \| Smlt1704 \| \| Smlt1705 \| \| Smlt1706 (pitA) \| \| Smlt1707 \| \| Smlt1708 \| \| Smlt1709 (pare) \| \| Smlt1710 \| \| Smlt1711 (pyrG) \| \| Smlt1712 (kdsA) \| \| Smlt1713 \| \| Smlt1714 \| \| Smlt1715 (eno) \| \| Smlt1716 (ftsB) \| \| Smlt1717 (ispD) \| \| Smlt1718 (ispF) \| \| Smlt1719 (truD) \| \| Smlt1720 \| \| Smlt1721 (surE) \| \| Smlt1722 (pcm) \| \| Smlt1723 \| \| Smlt1724 \| \| Smlt1725 \| \| Smlt1726 \| \| Smlt1727 \| \| Smlt1728 (ftsJ) \| \| Smlt1729 (ftsH) \| \| Smlt1730 \| \| Smlt1731 \| \| Smlt1733 \| \| Smlt1734 (folP) \| \| Smlt1735 (miaA) \| \| Smlt1736 (hfq) \| \| Smlt1737 (hflX) \| \| Smlt1738 \| \| Smlt1739 \| \| Smlt1740 (lexA) \| \| Smlt1741 (recA) \| \| Smlt1742 (recX) \| \| Smlt1743 (alaS) \| \| Smlt1744 \| \| Smlt_t31 \| \| Smlt1745 \| \| Smlt1746 \| \| Smlt1747 \| \| Smlt1748 (ace) \| \| Smlt1749 \| \| Smlt1750 \| \| Smlt1751 \| \| Smlt1753 \| \| Smlt1754 \| \| Smlt1755 (plcN1) \| \| Smlt1756 \| \| Smlt1757 \| \| Smlt1758 (adhB) \| \| Smlt1759 \| \| Smlt1760 \| \| Smlt1761 \| \| Smlt1762 \| \| Smlt1763 \| \| Smlt1764 \| \| Smlt_t32 \| \| Smlt_t34 \| \| Smlt_t35 \| \| Smlt1766 \| \| Smlt1767(thiD)  Smlt1768 \| \| Smlt1769 \| \| Smlt1770 \| \| Smlt1771 \| \| Smlt1772 \| \| Smlt1773 (dapA) \| \| Smlt1774 \| \| Smlt1774B \| \| Smlt1775 \| \| Smlt_t36 \| \| Smlt1777(pcnB) \| \| Smlt1778 \| \| Smlt1779 (panB) \| \| Smlt1780 (panC) \| \| Smlt1781 \| \| Smlt1782 (panD) \| \| Smlt1783 (pgi) \| \| Smlt1784 \| \| Smlt1785 \| \| Smlt1786 (ispG) \| \| Smlt1787 \| \| Smlt1788 \| \| Smlt1789 (kgdA) \| \| Smlt1790 (edd) \| \| Smlt1791 (pgl) \| \| Smlt1792 (glk) \| \| Smlt1793 (zwf) \| \| Smlt1794 \| \| Smlt1795 \| \| Smlt1796 (sdhC) \| \| Smlt1797 (sdhD) \| \| Smlt1798 (sdhA) \| \| Smlt1799 (sdhB) \| \| Smlt1800 \| \| Smlt1801 \| \| Smlt1802 \| \| Smlt1803 \| \| Smlt1804 \| \| Smlt1805 \| \| Smlt1806 (mutY) \| \| Smlt1807 (ftsY) \| \| Smlt1808 \| \| Smlt1809 (htpG) \| \| Smlt1810 \| \| Smlt1811 (coaD) \| \| Smlt1812 \| \| Smlt1813 (fdx) \| \| Smlt1814 (ggt) \| \| Smlt1815 \| \| Smlt1816 (psiF) \| \| Smlt1817 \| \| Smlt1818 \| \| Smlt1819 (upp) \| \| Smlt1820 \| \| Smlt1821 \| \| Smlt1822 \| \| Smlt1823 \| \| Smlt1824 \| \| Smlt1825 \| \| Smlt1826 \| \| Smlt1827 \| \| Smlt1828 \| \| Smlt1829 \| \| Smlt1830 (smeV) \| \| Smlt1831 (smeW) \| \| Smlt1832 \| \| Smlt1833 (smeX) \| \| Smlt1834 \| \| Smlt1835 \| \| Smlt1836 \| \| Smlt1837 \| \| Smlt1838 \| \| Smlt1839 \| \| Smlt1840 \| \| Smlt1841 \| \| Smlt1842 \| \| Smlt1843 \| \| Smlt1844 \| \| Smlt1844A \| \| Smlt1844B \| \| Smlt1845 \| \| Smlt1846 \| \| Smlt1846A \| \| Smlt1846B \| \| Smlt1849 \| \| Smlt1849A \| \| Smlt1850 \| \| Smlt1851 \| \| Smlt1852 \| \| Smlt1853 \| \| Smlt1854 \| \| Smlt1855 \| \| Smlt1856 \| \| Smlt1857 \| \| Smlt1858 \| \| Smlt1859 \| \| Smlt1860 \| \| Smlt1861 \| \| Smlt1862 \| \| Smlt1863 \| \| Smlt1864 \| \| Smlt1865 \| \| Smlt1866 \| \| Smlt1867 \| \| Smlt1868 \| \| Smlt1869 (B) \| \| Smlt1870 \| \| Smlt1871 \| \| Smlt1872 \| \| Smlt1873 \| \| Smlt1874 \| \| Smlt1875 \| \| Smlt1876 \| \| Smlt1877 \| \| Smlt1878 \| \| Smlt1879 \| \| Smlt1880 \| \| Smlt1881 \| \| Smlt1882 \| \| Smlt1883 \| \| Smlt1884 \| \| Smlt1885 \| \| Smlt1886 \| \| Smlt1887 \| \| Smlt1888 \| \| Smlt1889 \| \| Smlt1890 \| \| Smlt1891 \| \| Smlt1892 \| \| Smlt1893 \| \| Smlt1894 \| \| Smlt1895 \| \| Smlt1896 \| \| Smlt1897 \| \| Smlt1898 \| \| Smlt1900 \| \| Smlt1901 \| \| Smlt1902 \| \| Smlt1903 \| \| Smlt1905 \| \| Smlt1906 \| \| Smlt1907 \| \| Smlt1908 \| \| Smlt1911 \| \| Smlt1912 \| \| Smlt1913 \| \| Smlt1914 \| \| Smlt1915 \| \| Smlt1916 \| \| Smlt1917 \| \| Smlt1918(rdgC) \| \| Smlt1920 \| \| Smlt1921 \| \| Smlt1922 \| \| Smlt1923 \| \| Smlt1924 \| \| Smlt1925 \| \| Smlt1926 \| \| Smlt1927 \| \| Smlt1929 \| \| Smlt1930 \| \| Smlt1931 \| \| Smlt1932 \| \| Smlt1933 \| \| Smlt1934 \| \| Smlt1935 \| \| Smlt1936 \| \| Smlt1937 \| \| Smlt1938 \| \| Smlt1939 \| \| Smlt1940 \| \| Smlt1941 \| \| Smlt_t37 \| \| Smlt1944 \| \| Smlt1946 \| \| Smlt1947 \| \| Smlt1948 \| \| Smlt1949 \| \| Smlt1950 \| \| Smlt1951 \| \| Smlt1952 \| \| Smlt1953 \| \| Smlt1954 \| \| Smlt1955 \| \| Smlt1956 \| \| Smlt1957 \| \| Smlt1958 \| \| Smlt1959 \| \| Smlt1960 \| \| Smlt1961 \| \| Smlt1962 \| \| Smlt1963 \| \| Smlt1964 \| \| Smlt1965 \| \| Smlt1967 \| \| Smlt1968 \| \| Smlt1969 \| \| Smlt1970 \| \| Smlt1971 \| \| Smlt1972 \| \| Smlt1973 \| \| Smlt1974 \| \| Smlt1975 \| \| Smlt1976 \| \| Smlt1977 \| \| Smlt1978 \| \| Smlt1979 \| \| Smlt1980 \| \| Smlt1982 \| \| Smlt1983(smpB) \| \| Smlt1983A \| \| Smlt1984 \| \| Smlt1985 \| \| Smlt1986 (fur) \| \| Smlt1987 \| \| Smlt1988 \| \| Smlt1989 \| \| Smlt1990 (hrcA) \| \| Smlt1991 \| \| Smlt1992 (dnaK) \| \| Smlt1993 (dnaJ) \| \| Smlt1994 \| \| Smlt1995 \| \| Smlt1997(pdxK) \| \| Smlt1998 \| \| Smlt2000 \| \| Smlt2001 \| \| Smlt2002 \| \| Smlt2003 \| \| Smlt2004 \| \| Smlt2005 \| \| Smlt2006 \| \| Smlt2007 \| \| Smlt2008(queA) \| \| Smlt2009(tgt) \| \| Smlt2010(yajC) \| \| Smlt2011(secD) \| \| Smlt2012(secF) \| \| Smlt2015 \| \| Smlt2016 \| \| Smlt2017 \| \| Smlt2018 \| \| Smlt2019 \| \| Smlt2020 \| \| Smlt2022(cspG) \| \| Smlt2023 \| \| Smlt2024 \| \| Smlt2026(apt) \| \| Smlt2027(dbpA) \| \| Smlt2028 \| \| Smlt2029 \| \| Smlt2030 (uup) \| \| Smlt2031 \| \| Smlt2032 \| \| Smlt2033 (smmG) \| \| Smlt2034 (smmH) \| \| Smlt2035 (smmI) \| \| Smlt2036 \| \| Smlt2037 \| \| Smlt2038 \| \| Smlt2039 \| \| Smlt2040 \| \| Smlt2041 (rpmJ) \| \| Smlt2042 (cmk) \| \| Smlt2043 (rpsA) \| \| Smlt2044 (ihfB) \| \| Smlt2045 \| \| Smlt2046 \| \| Smlt2047(wbpL) \| \| Smlt2048(wbiI) \| \| Smlt2049(galU) \| \| Smlt2051 \| \| Smlt2052 \| \| Smlt2053 \| \| Smlt2054 (ndk) \| \| Smlt2055 \| \| Smlt2056 \| \| Smlt2057 \| \| Smlt2058 \| \| Smlt2059 \| \| Smlt2060 (engA) \| \| Smlt2061 \| \| Smlt2062 \| \| Smlt2063 \| \| Smlt2064 \| \| Smlt2065 \| \| Smlt2066 \| \| Smlt2068 \| \| Smlt2069(fold) \| \| Smlt2071(guaB) \| \| Smlt2072(guaA) \| \| Smlt2074 \| \| Smlt2075 \| \| Smlt2076 \| \| Smlt2077A \| \| Smlt2079 \| \| Smlt2080 \| \| Smlt2081 \| \| Smlt2082 \| \| Smlt2083 \| \| Smlt2084 \| \| Smlt2085 \| \| Smlt2086 \| \| Smlt2087 \| \| Smlt2088 \| \| Smlt2089 \| \| Smlt2090 \| \| Smlt2091 \| \| Smlt2092 \| \| Smlt2093 \| \| Smlt2094 \| \| Smlt2095 \| \| Smlt2096 (cheW) \| \| Smlt2097 \| \| Smlt2098 \| \| Smlt2099 \| \| Smlt2100 (ssuE) \| \| Smlt2101 \| \| Smlt2102 \| \| Smlt2103 \| \| Smlt2104 \| \| Smlt2105 \| \| Smlt2106 \| \| Smlt2107 \| \| Smlt2108 \| \| Smlt2109 \| \| Smlt2110 \| \| Smlt2111 \| \| Smlt2112 \| \| Smlt2113 \| \| Smlt2114 \| \| Smlt2115 \| \| Smlt2116 \| \| Smlt2117 \| \| Smlt2118 \| \| Smlt2119 \| \| Smlt2120 \| \| Smlt2121 \| \| Smlt2123 \| \| Smlt2124 \| \| Smlt2125 (spcN) \| \| Smlt2126 \| \| Smlt2127 (murB) \| \| Smlt2128 (pyrD) \| \| Smlt2129 \| \| Smlt2130 \| \| Smlt2131 \| \| Smlt2132 \| \| Smlt_t38 \| \| Smlt2133 \| \| Smlt2134 \| \| Smlt2135 \| \| Smlt2136 \| \| Smlt2137 \| \| Smlt2138 (nfxB) \| \| Smlt2139 \| \| Smlt2140 \| \| Smlt2141 \| \| Smlt2142 \| \| Smlt2143 \| \| Smlt2144 \| \| Smlt2144A \| \| Smlt2148 \| \| Smlt2149 \| \| Smlt2150 \| \| Smlt2151 \| \| Smlt2152 \| \| Smlt2153 \| \| Smlt2154 \| \| Smlt2156(thrA) \| \| Smlt2157(thrB) \| \| Smlt2158(thrC) \| \| Smlt2159(fnr1) \| \| Smlt2160(hiss) \| \| Smlt2161 \| \| Smlt2162 (hisG) \| \| Smlt2163 (hisD) \| \| Smlt2164 (hisC) \| \| Smlt2165 (hisB) \| \| Smlt21666 (hisH) \| \| Smlt2167 (hisA) \| \| Smlt2168 (hisF) \| \| Smlt2169 (hisI) \| \| Smlt2170 \| \| Smlt2171 \| \| Smlt2172 \| \| Smlt2173 \| \| Smlt2174 \| \| Smlt2175 \| \| Smlt2176 (actP) \| \| Smlt2177 \| \| Smlt2178 \| \| Smlt2179 \| \| Smlt2180 \| \| Smlt2181 \| \| Smlt2182 \| \| Smlt2183 (scrK) \| \| Smlt2184 \| \| Smlt2185(bmnA) \| \| Smlt2187(masA) \| \| Smlt2188 \| \| Smlt2189 \| \| Smlt2190 \| \| Smlt2191 \| \| Smlt2192 \| \| Smlt2193 \| \| Smlt2194 \| \| Smlt2195 (serA) \| \| Smlt2196 \| \| Smlt2197 \| \| Smlt2198 \| \| Smlt2199 \| \| Smlt2200 \| \| Smlt2201 (smeY) \| \| Smlt2202 (smeZ) \| \| Smlt2203 (smeQ) \| \| Smlt2204 \| \| Smlt2205 \| \| Smlt2206 \| \| Smlt2207 (hmgcL) \| \| Smlt2208 \| \| Smlt2209 \| \| Smlt2210 (feoA) \| \| Smlt2211 (feoB) \| \| Smlt2212 \| \| Smlt2213 \| \| Smlt2214 \| \| Smlt2215 \| \| Smlt2216 (fabG) \| \| Smlt2217 (dapB) \| \| Smlt2218 (carA) \| \| Smlt2219 (carB) \| \| Smlt2220 (greA) \| \| Smlt2221 \| \| Smlt2222 \| \| Smlt2223 (recJ) \| \| Smlt2224 \| \| Smlt2225 \| \| Smlt2226(lytT) \| \| Smlt2227(prfB) \| \| Smlt2229 \| \| Smlt2230 \| \| Smlt2231 (ilvK) \| \| Smlt2232 (lysS) \| \| Smlt2233 (rpfG) \| \| Smlt2234 (rpfC) \| \| Smlt2235 (rpfF) \| \| Smlt2236 (fadD) \| \| Smlt2237 (beta) \| \| Smlt2238 (betB) \| \| Smlt2239 (betI) \| \| Smlt2240 \| \| Smlt2241 (can) \| \| Smlt2242 (smpA) \| \| Smlt2243 \| \| Smlt2244 \| \| Smlt2245 (acnB) \| \| Smlt2246 \| \| Smlt2247 \| \| Smlt2248 (cheB) \| \| Smlt2249 (cheD) \| \| Smlt2250 (cheR) \| \| Smlt2251 \| \| Smlt2253 \| \| Smlt2254 \| \| Smlt2255 \| \| Smlt2256 (cheW) \| \| Smlt2257 \| \| Smlt2258 \| \| Smlt2260 (cheA) \| \| Smlt2261(cheY) \| \| Smlt2262 \| \| Smlt2263 \| \| Smlt2264 \| \| Smlt2265 (motB) \| \| Smlt2266 (motC) \| \| Smlt2267 (cheA) \| \| Smlt2268 (cheZ) \| \| Smlt2269 (cheY2) \| \| Smlt2270 (fliA) \| \| Smlt2271 \| \| Smlt2272 (flhF) \| \| Smlt2273 (flhA) \| \| Smlt2274 (flhB) \| \| Smlt2275 \| \| Smlt2276 \| \| Smlt2277 (fliR) \| \| Smlt2278 (fliQ) \| \| Smlt2279 (flip) \| \| Smlt2280 (fliO) \| \| Smlt2281 (fliN) \| \| Smlt2282 (fliM) \| \| Smlt2283 (fliL) \| \| Smlt2284 (fliK) \| \| Smlt2285 (fliJ) \| \| Smlt2286 (fliI) \| \| Smlt2287 (fliH) \| \| Smlt2288 (fliG) \| \| Smlt2289 (fliF) \| \| Smlt2290 (fliE) \| \| Smlt2291 \| \| Smlt2292 \| \| Smlt2293 \| \| Smlt2294 \| \| Smlt2295 \| \| Smlt2296 \| \| Smlt2297 \| \| Smlt2299 \| \| Smlt2300 \| \| Smlt2301 \| \| Smlt2302 (fliS) \| \| Smlt2303 (fliD) \| \| Smlt2304 (fliC) \| \| Smlt2305 (flaA) \| \| Smlt2306 \| \| Smlt2307 (flgL) \| \| Smlt2308 (flgK) \| \| Smlt2309 (flgJ) \| \| Smlt2310 (flgI) \| \| Smlt2311 (flgH) \| \| Smlt2312 (flgG) \| \| Smlt2313 (flgF) \| \| Smlt2314 (flgE) \| \| Smlt2315 (flgD) \| \| Smlt2316 (flgC) \| \| Smlt2317 (flgB) \| \| Smlt2318 \| \| Smlt2319 (flgA) \| \| Smlt2320 (flgM) \| \| Smlt2321 (flgN) \| \| Smlt2322 \| \| Smlt2323 \| \| Smlt2324 \| \| Smlt2325 \| \| Smlt2326 \| \| Smlt2328 \| \| Smlt2329 \| \| Smlt2330 \| \| Smlt2331 \| \| Smlt2332 (mnmA) \| \| Smlt2333 \| \| Smlt2334 \| \| Smlt2335 (clpS) \| \| Smlt2336 \| \| Smlt2337 (clpA) \| \| Smlt2338 \| \| Smlt2339 (infA) \| \| Smlt2340 (aat) \| \| Smlt2341 \| \| Smlt2342 \| \| Smlt2343 (trxB) \| \| Smlt2344 (ftsK) \| \| Smlt2345 \| \| Smlt2346(lolA) \| \| Smlt2348 \| \| Smlt2349 \| \| Smlt2350 \| \| Smlt2351 \| \| Smlt2352 \| \| Smlt2353 \| \| Smlt2354 \| \| Smlt2355 \| \| Smlt2356 \| \| Smlt2357 \| \| Smlt2358 \| \| Smlt2360 \| \| Smlt2361 \| \| Smlt2362 \| \| Smlt2364 \| \| Smlt2365 \| \| Smlt2366 \| \| Smlt2367 \| \| Smlt2368 \| \| Smlt2369 \| \| Smlt2370 \| \| Smlt2371 \| \| Smlt2372 \| \| Smlt2373 \| \| Smlt2374 \| \| Smlt2375 \| \| Smlt2377 \| \| Smlt2378 \| \| Smlt2379 \| \| Smlt2380 \| \| Smlt2381 \| \| Smlt2382  Smlt2383 \| \| Smlt2384 \| \| Smlt2385 \| \| Smlt2386 \| \| Smlt2387 \| \| Smlt2388 \| \| Smlt2389 \| \| Smlt2390 \| \| Smlt2391 \| \| Smlt2392 \| \| Smlt2393 \| \| Smlt2394 \| \| Smlt2395 \| \| Smlt2397 \| \| Smlt2398 \| \| Smlt2399 \| \| Smlt2400 \| \| Smlt2401 \| \| Smlt2403 \| \| Smlt2404 \| \| Smlt2405 \| \| Smlt2406 \| \| Smlt2407 \| \| Smlt2407A \| \| Smlt2408 \| \| Smlt2409 (merR) \| \| Smlt2410 (merT) \| \| Smlt2411 (merP) \| \| Smlt2412 (merA) \| \| Smlt2413 \| \| Smlt2414 \| \| Smlt2416 \| \| Smlt2417 \| \| Smlt2418 \| \| Smlt2419 (arsR) \| \| Smlt2420 \| \| Smlt2421 (arsC) \| \| Smlt2422 (arsH) \| \| Smlt2423 (arsR2) \| \| Smlt2424 (arsC2) \| \| Smlt2425 (arsB) \| \| Smlt2426 (trxB) \| \| Smlt2427 \| \| Smlt2428 \| \| Smlt2431 \| \| Smlt2432 (smmD) \| \| Smlt2433 (cusB) \| \| Smlt2434 (cusA) \| \| Smlt2435 \| \| Smlt2436 \| \| Smlt2437 \| \| Smlt2438 \| \| Smlt2439 \| \| Smlt2440 (copF) \| \| Smlt2441 (copD) \| \| Smlt2442 (copC) \| \| Smlt2443 \| \| Smlt2444 (copG) \| \| Smlt2445 \| \| Smlt2446 (copM) \| \| Smlt2447 (copB) \| \| Smlt2448 (copA) \| \| Smlt2449 (copL) \| \| Smlt2450 \| \| Smlt2451 \| \| Smlt2452 \| \| Smlt2453 \| \| Smlt2454 \| \| Smlt2455 \| \| Smlt2456 \| \| Smlt2457 \| \| Smlt2458 \| \| Smlt2459 \| \| Smlt2460 \| \| Smlt2461 \| \| Smlt2462 \| \| Smlt2463 \| \| Smlt2464 \| \| Smlt2465 (tnpA) \| \| Smlt2466 \| \| Smlt2467 \| \| Smlt2468 \| \| Smlt2469 \| \| Smlt2470 \| \| Smlt2471 \| \| Smlt2472 \| \| Smlt2474 \| \| Smlt2475 \| \| Smlt2476 \| \| Smlt2477 \| \| Smlt2478 \| \| Smlt2479 \| \| Smlt2479A \| \| Smlt2481 \| \| Smlt2482 \| \| Smlt2482A \| \| Smlt2482B \| \| Smlt2486 \| \| Smlt2487 \| \| Smlt2488 \| \| Smlt2489 \| \| Smlt2490 \| \| Smlt2491 \| \| Smlt2492 \| \| Smlt2493 \| \| Smlt2494 \| \| Smlt2495 \| \| Smlt2496 \| \| Smlt2497 \| \| Smlt2498 \| \| Smlt2499 \| \| Smlt2500 \| \| Smlt2501 \| \| Smlt2502 \| \| Smlt2503 (yagR) \| \| Smlt2504 (yagS) \| \| Smlt2505 (yagT) \| \| Smlt2506 \| \| Smlt2507 \| \| Smlt2508 \| \| Smlt2509 \| \| Smlt2510 \| \| Smlt2511 \| \| Smlt2512 \| \| Smlt2513 \| \| Smlt2514 \| \| Smlt2516 \| \| Smlt2518 \| \| Smlt2519 \| \| Smlt2521 \| \| Smlt2522 \| \| Smlt2523 \| \| Smlt2524 \| \| Smlt2525 \| \| Smlt2526 \| \| Smlt2527 \| \| Smlt2528 \| \| Smlt2529 \| \| Smlt2530 \| \| Smlt2531 \| \| Smlt2532 \| \| Smlt2533 \| \| Smlt2534 \| \| Smlt2535 \| \| Smlt2536 \| \| Smlt2537 \| \| Smlt2538 \| \| Smlt2540 \| \| Smlt2541 \| \| Smlt2543 \| \| Smlt2544 \| \| Smlt2545 \| \| Smlt2546 \| \| Smlt2547 \| \| Smlt2548 \| \| Smlt2549 \| \| Smlt2550 \| \| Smlt2551 \| \| Smlt2552 \| \| Smlt2553 \| \| Smlt2554 \| \| Smlt2555 \| \| Smlt2556 \| \| Smlt2557 (fruK) \| \| Smlt2558 (fruA) \| \| Smlt2559 (rpfN) \| \| Smlt2560 \| \| Smlt2561 \| \| Smlt2562 \| \| Smlt2563 \| \| Smlt2566 \| \| Smlt2567 \| \| Smlt2568 \| \| Smlt2569 \| \| Smlt2570 \| \| Smlt2571 \| \| Smlt2572 \| \| Smlt2573 \| \| Smlt2574 \| \| Smlt2575 \| \| Smlt2576 \| \| Smlt2577 (uvrA2) \| \| Smlt2578 \| \| Smlt2579 \| \| Smlt2580 \| \| Smlt2581 \| \| Smlt2582 \| \| Smlt2583 \| \| Smlt2584 (msuE) \| \| Smlt2585 \| \| Smlt2586 (metE) \| \| Smlt2587 \| \| Smlt2588 \| \| Smlt2589 \| \| Smlt2590 \| \| Smlt2591 \| \| Smlt2592 \| \| Smlt2593 \| \| Smlt2594 \| \| Smlt2595 \| \| Smlt2596 \| \| Smlt2597 (ssuE) \| \| Smlt2598 \| \| Smlt2599 \| \| Smlt2600 \| \| Smlt2601 \| \| Smlt2602 \| \| Smlt2603 \| \| Smlt2604 \| \| Smlt2605 \| \| Smlt2606 \| \| Smlt2607 \| \| Smlt2608 \| \| Smlt2609 \| \| Smlt2610 \| \| Smlt2611 \| \| Smlt2612 \| \| Smlt2613 \| \| Smlt2614 \| \| Smlt2615 \| \| Smlt2616 \| \| Smlt2617 \| \| Smlt2618 \| \| Smlt2619 \| \| Smlt2620 \| \| Smlt2621 \| \| Smlt2622 \| \| Smlt2623 \| \| Smlt2624 \| \| Smlt2625 \| \| Smlt2626 \| \| Smlt2627 \| \| Smlt2628 \| \| Smlt2629 \| \| Smlt2630 \| \| Smlt2631 \| \| Smlt2632 \| \| Smlt2633 \| \| Smlt2634 \| \| Smlt2635 \| \| Smlt2636 \| \| Smlt2637 \| \| Smlt2638 \| \| Smlt2639 \| \| Smlt2640 \| \| Smlt2641 \| \| Smlt2642 \| \| Smlt2643 \| \| Smlt2644 \| \| Smlt2645 \| \| Smlt2646 \| \| Smlt2647 \| \| Smlt2648 \| \| Smlt2649 \| \| Smlt2650 \| \| Smlt2652(trbP) \| \| Smlt2653(pip) \| \| Smlt2654 \| \| Smlt2655 (mgtA) \| \| Smlt2656 (mgtC) \| \| Smlt2657 \| \| Smlt2658 \| \| Smlt2659 \| \| Smlt2660 \| \| Smlt2661 \| \| Smlt2662 \| \| Smlt2663 \| \| Smlt2664 \| \| Smlt2665 \| \| Smlt2666 \| \| Smlt2667 \| \| Smlt2668 \| \| Smlt2669 \| \| Smlt2670 \| \| Smlt2671 \| \| Smlt2372(fdsC) \| \| Smlt2675 \| \| Smlt2676 \| \| Smlt2677 \| \| Smlt2678 \| \| Smlt2679 \| \| Smlt2680 \| \| Smlt2681 \| \| Smlt2682 \| \| Smlt2683 \| \| Smlt2684 \| \| Smlt2685 \| \| Smlt2686 \| \| Smlt2687 (dkgB) \| \| Smlt2688 \| \| Smlt2689 \| \| Smlt2690 \| \| Smlt2691 (copC) \| \| Smlt2692(copD) \| \| Smlt2693 \| \| Smlt2694 \| \| Smlt2695 \| \| Smlt2696 \| \| Smlt2697 (smmA) \| \| Smlt2698 (smmB) \| \| Smlt2699 (smmC) \| \| Smlt2700 \| \| Smlt2701 \| \| Smlt2702 \| \| Smlt2703 \| \| Smlt2704 \| \| Smlt2706 \| \| Smlt2707 \| \| Smlt2708 \| \| Smlt2709 \| \| Smlt2710 \| \| Smlt2711 \| \| Smlt2712 \| \| Smlt2713 \| \| Smlt2714 \| \| Smlt2715 \| \| Smlt2716 \| \| Smlt2718 \| \| Smlt2719 \| \| Smlt2720 \| \| Smlt2721 \| \| Smlt2722 \| \| Smlt2723 \| \| Smlt2724 \| \| Smlt2725 \| \| Smlt2726 (exbD) \| \| Smlt2727 (exbB) \| \| Smlt2728 \| \| Smlt2729 \| \| Smlt2730 (gspC) \| \| Smlt2731 (gspH) \| \| Smlt2732 (gspI) \| \| Smlt2733 (gspJ) \| \| Smlt2735 \| \| Smlt2737 \| \| Smlt2738(pstS) \| \| Smlt2740(gspF) \| \| Smlt2741(gspE) \| \| Smlt2742 (spD) \| \| Smlt2743(gspM) \| \| Smlt2744 (gspL) \| \| Smlt2745(gspK) \| \| Smlt2746(gspG) \| \| Smlt2747 \| \| Smlt2748 \| \| Smlt2749 (fecR) \| \| Smlt2750 \| \| Smlt2751 \| \| Smlt2753 \| \| Smlt2754 \| \| Smlt2755 \| \| Smlt2756 (glgX) \| \| Smlt2757 \| \| Smlt2758 \| \| Smlt2759 \| \| Smlt2760 (glgB) \| \| Smlt2761 (glgA) \| \| Smlt2762 \| \| Smlt2763 (cysJ) \| \| Smlt2764 \| \| Smlt2765 \| \| Smlt2766 \| \| Smlt2767 (fnr2) \| \| Smlt2768 \| \| Smlt2769 (narK2) \| \| Smlt2770 \| \| Smlt2771 (narI) \| \| Smlt2772 (narJ) \| \| Smlt2773 (narH) \| \| Smlt2774 (narG) \| \| Smlt2775 (nark) \| \| Smlt2776 \| \| Smlt2777 (mobA) \| \| Smlt2778 (moaE) \| \| Smlt2779 (moaD) \| \| Smlt2780 (moeA) \| \| Smlt2781 (moaC) \| \| Smlt2782 (moaA) \| \| Smlt2783 \| \| Smlt2784 \| \| Smlt2785 \| \| Smlt2786 \| \| Smlt2787 \| \| Smlt2788 \| \| Smlt2789 \| \| Smlt2790 \| \| Smlt2791 \| \| Smlt2792 \| \| Smlt2793 \| \| Smlt2794 \| \| Smlt2795 \| \| Smlt2796 \| \| Smlt2797 \| \| Smlt2798 \| \| Smlt2799 \| \| Smlt2800 \| \| Smlt2801 \| \| Smlt2802 \| \| Smlt2803 (dgk) \| \| Smlt2804 \| \| Smlt2805 \| \| Smlt2806 \| \| Smlt2807 \| \| Smlt2808 \| \| Smlt2809 \| \| Smlt2816 \| \| Smlt2817 (entA) \| \| Smlt2818 (entF) \| \| Smlt2819 \| \| Smlt2820 \| \| Smlt2821 \| \| Smlt2822 (entC) \| \| Smlt2823 \| \| Smlt2824 \| \| Smlt2825 \| \| Smlt2826 \| \| Smlt2827 \| \| Smlt2828 (soda) \| \| Smlt2829 \| \| Smlt2830 \| \| Smlt2831 \| \| Smlt2832 \| \| Smlt2833 \| \| Smlt2834 \| \| Smlt2835 \| \| Smlt2836 \| \| Smlt2837 \| \| Smlt2838 \| \| Smlt2839 \| \| Smlt2840 \| \| Smlt2841 (nrdF) \| \| Smlt2842 (nrdE) \| \| Smlt2843 \| \| Smlt2844 \| \| Smlt2845 \| \| Smlt2846 (betT) \| \| Smlt2847 \| \| Smlt2848 \| \| Smlt2849 \| \| Smlt2850 \| \| Smlt2851 \| \| Smlt2852 \| \| Smlt2853 \| \| Smlt2857 \| \| Smlt2858 \| \| Smlt2859 \| \| Smlt2860 \| \| Smlt2861 \| \| Smlt2862 \| \| Smlt2863 \| \| Smlt2864 \| \| Smlt2865 \| \| Smlt2866 \| \| Smlt2867 \| \| Smlt2868 \| \| Smlt2869 \| \| Smlt2870 \| \| Smlt2871 \| \| Smlt2872 \| \| Smlt2873 \| \| Smlt2874 \| \| Smlt2875 \| \| Smlt2876 \| \| Smlt2877 \| \| Smlt2878 \| \| Smlt2880 \| \| Smlt2882(cpo) \| \| Smlt2883 \| \| Smlt2884 \| \| Smlt2885 \| \| Smlt2886 \| \| Smlt2887 \| \| Smlt2888 \| \| Smlt2889 \| \| Smlt2890 \| \| Smlt2891 \| \| Smlt2892 \| \| Smlt2893 \| \| Smlt2894 \| \| Smlt2895 \| \| Smlt2896 \| \| Smlt2897 \| \| Smlt2898 (atzC2) \| \| Smlt2899 \| \| Smlt2900 \| \| Smlt2901 \| \| Smlt2902 \| \| Smlt2903 \| \| Smlt2904 \| \| Smlt2905 \| \| Smlt2906 \| \| Smlt2907 (dld) \| \| Smlt2908 (lctD) \| \| Smlt2909 (lctR) \| \| Smlt2910 (lctP) \| \| Smlt2911 \| \| Smlt2912 \| \| Smlt2913 \| \| Smlt2915 \| \| Smlt2916 \| \| Smlt2917 \| \| Smlt2918 \| \| Smlt2919 (cgb) \| \| Smlt2920 \| \| Smlt2921 \| \| Smlt2922 \| \| Smlt2923 \| \| Smlt2924 \| \| Smlt2925 \| \| Smlt2926 \| \| Smlt2927 \| \| Smlt2928 (dnaE2) \| \| Smlt2929 \| \| Smlt2930 \| \| Smlt2931 \| \| Smlt2932 \| \| Smlt2933 \| \| Smlt2934 \| \| Smlt2935 \| \| Smlt2936 \| \| Smlt2937 \| \| Smlt2938 \| \| Smlt2939 \| \| Smlt2940 \| \| Smlt2941 \| \| Smlt2942 \| \| Smlt2943 \| \| Smlt2944 \| \| Smlt2946(dsbG) \| \| Smlt2947(dsbE) \| \| Smlt2948 \| \| Smlt2949 (zntR) \| \| Smlt_t39 \| \| Smlt_t40 \| \| Smlt_t41 \| \| Smlt_t42 \| \| Smlt_t43 \| \| Smlt_t45 \| \| Smlt2950 \| \| Smlt2951 \| \| Smlt2952 \| \| Smlt_t46 \| \| Smlt2953 \| \| Smlt2954 (mcpA) \| \| Smlt2955 \| \| Smlt2956 \| \| Smlt2957 \| \| Smlt2958 \| \| Smlt2959 \| \| Smlt2960 (tex) \| \| Smlt2961 \| \| Smlt2962 \| \| Smlt2963 \| \| Smlt2964 (phaC) \| \| Smlt2965 (phaE) \| \| Smlt2966 \| \| Smlt2967 \| \| Smlt2970 \| \| Smlt2971 \| \| Smlt2972 (mutX) \| \| Smlt2973 \| \| Smlt2974 \| \| Smlt2975(pps) \| \| Smlt2977 \| \| Smlt2978 \| \| Smlt2979 \| \| Smlt2981(orn) \| \| Smlt2982 \| \| Smlt2983  Smlt2984 \| \| Smlt2985 \| \| Smlt2986 \| \| Smlt2987 \| \| Smlt2988 \| \| Smlt2989 \| \| Smlt2990 \| \| Smlt2991 \| \| Smlt2992 \| \| Smlt2993 \| \| Smlt2994 \| \| Smlt2995 \| \| Smlt2996 \| \| Smlt2997 (trwI) \| \| Smlt2998 \| \| Smlt2999 \| \| Smlt3000 \| \| Smlt3001 \| \| Smlt3002 \| \| Smlt3003 \| \| Smlt3004 \| \| Smlt3005 \| \| Smlt3006 \| \| Smlt3007 \| \| Smlt3008 \| \| Smlt3009 \| \| Smlt3010 \| \| Smlt3011 \| \| Smlt3012 \| \| Smlt3013 \| \| Smlt3014 \| \| Smlt3015 \| \| Smlt3016 \| \| Smlt3017 \| \| Smlt3018 \| \| Smlt3019 \| \| Smlt3020 \| \| Smlt3021 \| \| Smlt3022 \| \| Smlt3023 \| \| Smlt3024 \| \| Smlt3025 \| \| Smlt3026 \| \| Smlt3027 \| \| Smlt3028 \| \| Smlt3029 \| \| Smlt3030 \| \| Smlt3031 \| \| Smlt3032 \| \| Smlt3033 \| \| Smlt3034 \| \| Smlt3035 \| \| Smlt3036 \| \| Smlt3037 \| \| Smlt3038 \| \| Smlt3039 (umuC) \| \| Smlt3040 \| \| Smlt3041 \| \| Smlt3042 \| \| Smlt3043 \| \| Smlt3044 \| \| Smlt3045 \| \| Smlt3046 \| \| Smlt3048 \| \| Smlt3049 \| \| Smlt3050 \| \| Smlt3051 (traA) \| \| Smlt3052 \| \| Smlt3053 (traD) \| \| Smlt3054 \| \| Smlt3055 \| \| Smlt3056 \| \| Smlt3057 \| \| Smlt3058 \| \| Smlt3059 \| \| Smlt3060 \| \| Smlt3061 \| \| Smlt3062 \| \| Smlt3062AA \| \| Smlt3062A \| \| Smlt3065 \| \| Smlt3066 \| \| Smlt3067 \| \| Smlt3068 \| \| Smlt3069 \| \| Smlt3070 \| \| Smlt3071 \| \| Smlt3073 \| \| Smlt3074 \| \| Smlt3075 \| \| Smlt3076 \| \| Smlt3077 \| \| Smlt3078 \| \| Smlt3079 \| \| Smlt3080 \| \| Smlt3081 \| \| Smlt3082 \| \| Smlt3083 \| \| Smlt3083A \| \| Smlt3084 \| \| Smlt3085 \| \| Smlt3086 \| \| Smlt_t47 \| \| Smlt3087 \| \| Smlt3089 \| \| Smlt3090 (acpD) \| \| Smlt3091 \| \| Smlt3092 (serS) \| \| Smlt3093 \| \| Smlt3094 \| \| Smlt3095 \| \| Smlt3096 (aroA) \| \| Smlt3097 (pheA) \| \| Smlt3098 (serC) \| \| Smlt3099 \| \| Smlt3100 \| \| Smlt3101(phaF) \| \| Smlt3103 \| \| Smlt3104 \| \| Smlt3105 \| \| Smlt3106 \| \| Smlt3107 (hutI) \| \| Smlt3108 (hutH1) \| \| Smlt3109 \| \| Smlt3110 \| \| Smlt3111 \| \| Smlt3112 \| \| Smlt3114 \| \| Smlt3115 \| \| Smlt3116 \| \| Smlt3117 \| \| Smlt3118 \| \| Smlt3119 \| \| Smlt3120 \| \| Smlt3121 \| \| Smlt3122 \| \| Smlt3123 \| \| Smlt3124 \| \| Smlt3125 \| \| Smlt3126 \| \| Smlt3128 \| \| Smlt3129 \| \| Smlt3130 \| \| Smlt3131 \| \| Smlt3132 \| \| Smlt3133 \| \| Smlt3134 \| \| Smlt3135 \| \| Smlt3136 (gcd) \| \| Smlt3137 (gyrA) \| \| Smlt3138 (mtnA) \| \| Smlt3139 \| \| Smlt3140 \| \| Smlt3141 \| \| Smlt3142 (ligA) \| \| Smlt3143 \| \| Smlt3144 (zipA) \| \| Smlt3145 \| \| Smlt3146 \| \| Smlt3147 \| \| Smlt3148(rplI) \| \| Smlt3149(rpsR) \| \| Smlt3150(rpsF) \| \| Smlt3152 \| \| Smlt3153 \| \| Smlt3154 (asnC) \| \| Smlt3155 \| \| Smlt3156 \| \| Smlt3157 \| \| Smlt3158 \| \| Smlt3159 (nbaC) \| \| Smlt3160 \| \| Smlt3161 (qbsG) \| \| Smlt3162 (sbcB) \| \| Smlt3163 \| \| Smlt3164 \| \| Smlt3165 \| \| Smlt3166 (ppnK) \| \| Smlt3167 \| \| Smlt3169 \| \| Smlt3170 (smeG) \| \| Smlt3171 (acrD) \| \| Smlt3172 \| \| Smlt3173 \| \| Smlt3174 (bcd) \| \| Smlt3175 \| \| Smlt3176 (metH2) \| \| Smlt3177 (metH1) \| \| Smlt3178 \| \| Smlt3179 \| \| Smlt3179A \| \| Smlt3181 \| \| Smlt3182 \| \| Smlt3183 \| \| Smlt3184 \| \| Smlt3185 \| \| Smlt3186 \| \| Smlt3187 \| \| Smlt3188 \| \| Smlt3189 \| \| Smlt3190 \| \| Smlt3191(fumC) \| \| Smlt3193(purB) \| \| Smlt3195 \| \| Smlt3196 \| \| Smlt3197 (sucA) \| \| Smlt3198 (sucB) \| \| Smlt3199 (odhL) \| \| Smlt3200 \| \| Smlt3201 \| \| Smlt3202 (phoD) \| \| Smlt3203 \| \| Smlt3204 (dnaB) \| \| Smlt3205 \| \| Smlt3206 \| \| Smlt3207 \| \| Smlt3208 \| \| Smlt3209 \| \| Smlt3210 \| \| Smlt3211 \| \| Smlt3212 \| \| Smlt3213 \| \| Smlt3214 \| \| Smlt3215 \| \| Smlt3216 \| \| Smlt3217 \| \| Smlt3218 \| \| Smlt3219 (arsC2) \| \| Smlt3220 \| \| Smlt3221 \| \| Smlt3222 \| \| Smlt3223 \| \| Smlt3224 \| \| Smlt3226 \| \| Smlt3227 (fpr) \| \| Smlt3228 \| \| Smlt3229 \| \| Smlt3230 \| \| Smlt3231 (cfa) \| \| Smlt3232 \| \| Smlt3233 \| \| Smlt3234 \| \| Smlt3235 \| \| Smlt3236 \| \| Smlt3237 \| \| Smlt3238 (soda) \| \| Smlt3239 \| \| Smlt3240 \| \| Smlt3241 \| \| Smlt3242 \| \| Smlt3243 (phhB) \| \| Smlt3244 \| \| Smlt3245 \| \| Smlt3246 (rluC) \| \| Smlt3247 (rnE) \| \| Smlt3248 \| \| Smlt3249 \| \| Smlt3250 \| \| Smlt3251 \| \| Smlt3252 \| \| Smlt3253 \| \| Smlt3254 \| \| Smlt3255 \| \| Smlt3256 \| \| Smlt3257 \| \| Smlt3259 \| \| Smlt3260 \| \| Smlt3261 (rluB) \| \| Smlt3262 \| \| Smlt3263 \| \| Smlt3264 \| \| Smlt3265 \| \| Smlt3266 \| \| Smlt3267 \| \| Smlt3268 (ccmA) \| \| Smlt3269 (ccmB) \| \| Smlt3270 (ccmC) \| \| Smlt3271 \| \| Smlt3272 (ccmE) \| \| Smlt3273 (ccmF) \| \| Smlt3274 (dsbE) \| \| Smlt3275 (ccmH) \| \| Smlt3276 (cycH) \| \| Smlt3277 (metX) \| \| Smlt3278 \| \| Smlt3279(cydC) \| \| Smlt3280(cydD) \| \| Smlt3282(cydA) \| \| Smlt3283(appB) \| \| Smlt3284 \| \| Smlt_t48 \| \| Smlt_t49 \| \| Smlt3287 \| \| Smlt3289(hmsH) \| \| Smlt3290(hmsF) \| \| Smlt3291 (hmsR) \| \| Smlt3292(hmsS) \| \| Smlt3293(proA) \| \| Smlt3294(proB) \| \| Smlt3295 \| \| Smlt3296 (argH) \| \| Smlt3297 (argC) \| \| Smlt3298 \| \| Smlt3299 \| \| Smlt3300 \| \| Smlt3301 (argG) \| \| Smlt3302 \| \| Smlt3303 \| \| Smlt3304 \| \| Smlt3305 (cysS) \| \| Smlt3306 (sufE) \| \| Smlt3307 \| \| Smlt3308 \| \| Smlt3309 \| \| Smlt3310 \| \| Smlt3311 \| \| Smlt3312 \| \| Smlt3313 \| \| Smlt3314 (ubiG) \| \| Smlt3315 \| \| Smlt3316 (efp) \| \| Smlt3317 \| \| Smlt3318 \| \| Smlt3319 \| \| Smlt3320 \| \| Smlt3321(suhB) \| \| Smlt3323 (htpX) \| \| Smlt3324 \| \| Smlt3325 (phbB) \| \| Smlt3326 \| \| Smlt3327 \| \| Smlt3328 \| \| Smlt3329 (mutL) \| \| Smlt3330 \| \| Smlt3331 \| \| Smlt3332 \| \| Smlt3333 \| \| Smlt3334 (xseA) \| \| Smlt3335 \| \| Smlt3336 \| \| Smlt3337 \| \| Smlt3338 (rnd) \| \| Smlt3339 \| \| Smlt3340 \| \| Smlt_t50 \| \| Smlt3342 \| \| Smlt3344 \| \| Smlt3345 \| \| Smlt3346 \| \| Smlt3347 \| \| Smlt_t51 \| \| Smlt_t52 \| \| Smlt3348 \| \| Smlt3351 \| \| Smlt3352 \| \| Smlt3354 \| \| Smlt3355(dxs) \| \| Smlt3357 \| \| Smlt3358 \| \| Smlt3359 \| \| Smlt3360 \| \| Smlt3361 \| \| Smlt3362 \| \| Smlt3363 \| \| Smlt3364 \| \| Smlt3365 \| \| Smlt3366 \| \| Smlt3367 \| \| Smlt3368 \| \| Smlt3369 \| \| Smlt3370 \| \| Smlt_t53 \| \| Smlt3371 \| \| Smlt3372 (ihfA) \| \| Smlt3373 (pheT) \| \| Smlt3374 (pheS) \| \| Smlt3375 (rplT) \| \| Smlt3376 (RL35) \| \| Smlt3377 (infC) \| \| Smlt3378 (thrS) \| \| Smlt3379 \| \| Smlt3380 \| \| Smlt3381 \| \| Smlt3382 \| \| Smlt3383 (chiA1) \| \| Smlt3384 \| \| Smlt3385 (pnp) \| \| Smlt3386 (rpsO) \| \| Smlt3387 (truB) \| \| Smlt3388 (rbfA) \| \| Smlt3389 (infB) \| \| Smlt3390 (nusA) \| \| Smlt3391 \| \| Smlt_t54 \| \| Smlt_t56 \| \| Smlt3392(nuoN) \| \| Smlt3393(nuoM) \| \| Smlt3394(nuoL) \| \| Smlt3395(nuoK) \| \| Smlt3396(nuoJ) \| \| Smlt3397(nuoI) \| \| Smlt3398(nuoH) \| \| Smlt3399(nuoG) \| \| Smlt3400(nuoF) \| \| Smlt3401(nuoE) \| \| Smlt3402(nuoD) \| \| Smlt3403(nuoC) \| \| Smlt3404(nuoB) \| \| Smlt3405(nuoA) \| \| Smlt_t57 \| \| Smlt3406(secG) \| \| Smlt3407(tpiA) \| \| Smlt3409 \| \| Smlt3410 \| \| Smlt3411 \| \| Smlt3412 \| \| Smlt3413(galE) \| \| Smlt3414(glmM) \| \| Smlt3415(accD) \| \| Smlt3417(trpA) \| \| Smlt3418 \| \| Smlt3419 (trpB) \| \| Smlt3420 \| \| Smlt3421 \| \| Smlt3422 \| \| Smlt3423 (trpF) \| \| Smlt3424 (truA) \| \| Smlt3425 \| \| Smlt3426 (fimV) \| \| Smlt3427 (asd) \| \| Smlt3428 \| \| Smlt3429 (aroC) \| \| Smlt3430 \| \| Smlt3431 \| \| Smlt3432 (psd) \| \| Smlt3433 \| \| Smlt3434 (mltD) \| \| Smlt3435 \| \| Smlt3436 (greB) \| \| Smlt3437 \| \| Smlt3438 (rimO) \| \| Smlt3439 (clcD) \| \| Smlt3440 \| \| Smlt3441 \| \| Smlt3442 (dcd) \| \| Smlt3443 \| \| Smlt3444 \| \| Smlt3446 \| \| Smlt3447(pepO) \| \| Smlt3449 \| \| Smlt3450 \| \| Smlt3451 \| \| Smlt3452 \| \| Smlt3453 \| \| Smlt3454 \| \| Smlt3455 \| \| Smlt3456 (gor) \| \| Smlt3457 \| \| Smlt3458 \| \| Smlt3459 \| \| Smlt3460 \| \| Smlt3461 \| \| Smlt3462 \| \| Smlt3463 \| \| Smlt3464 \| \| Smlt3465 (mesJ) \| \| Smlt3466 (xseB) \| \| Smlt3467 (ispA) \| \| Smlt3468 \| \| Smlt3469 \| \| Smlt3470 \| \| Smlt3471 (tldD) \| \| Smlt3472 \| \| Smlt3473 (rnG) \| \| Smlt3474 \| \| Smlt3475 \| \| Smlt3477 \| \| Smlt3478 \| \| Smlt3479 (arcD) \| \| Smlt_t58 \| \| Smlt3480 \| \| Smlt3481 \| \| Smlt3482 (nadD) \| \| Smlt3483 (holA) \| \| Smlt3484 \| \| Smlt3485 (leuS) \| \| Smlt3486 \| \| Smlt3487 \| \| Smlt3488 \| \| Smlt3489 \| \| Smlt3490 \| \| Smlt3491 \| \| Smlt3492 \| \| Smlt3493 \| \| Smlt3494 \| \| Smlt3495 \| \| Smlt3496 \| \| Smlt3497 \| \| Smlt3498 (metG) \| \| Smlt3499 \| \| Smlt3500 \| \| Smlt3501 \| \| Smlt3502 (lpiA) \| \| Smlt3503 (acvB) \| \| Smlt3504 \| \| Smlt3505 \| \| Smlt3506 \| \| Smlt3507 \| \| Smlt3508 (phnA) \| \| Smlt3509 \| \| Smlt3510 \| \| Smlt3511 \| \| Smlt3512 \| \| Smlt3513 \| \| Smlt3514 (sigma) \| \| Smlt3515 \| \| Smlt3517 \| \| Smlt3518 \| \| Smlt3519 \| \| Smlt3520 \| \| Smlt3521 \| \| Smlt3522 \| \| Smlt3523 (vdh) \| \| Smlt3524 (sphB) \| \| Smlt3525 \| \| Smlt3526 \| \| Smlt3527 \| \| Smlt3528 \| \| Smlt3529 \| \| Smlt3530 (ligB) \| \| Smlt3531 \| \| Smlt3532 \| \| Smlt3533 \| \| Smlt3534 \| \| Smlt3535 (cspA) \| \| Smlt3536 \| \| Smlt3537 (hpt) \| \| Smlt3538 (nagZ) \| \| Smlt3539 \| \| Smlt3540 \| \| Smlt3541 (rumA) \| \| Smlt3542 \| \| Smlt3544 \| \| Smlt3545 \| \| Smlt3546 \| \| Smlt3547 (recO) \| \| Smlt3548 (era) \| \| Smlt3549 (rnc) \| \| Smlt3550 \| \| Smlt3551 (lepB) \| \| Smlt3552 (lepA) \| \| Smlt3553 \| \| Smlt3554 (rseA) \| \| Smlt3555 (rpoE) \| \| Smlt3556 \| \| Smlt3558 \| \| Smlt3559 (aqpZ) \| \| Smlt3560 \| \| Smlt3561 \| \| Smlt3562 (cstA) \| \| Smlt3563 \| \| Smlt3565 \| \| Smlt3566 \| \| Smlt3567 \| \| Smlt3568 \| \| Smlt3569 \| \| Smlt3570 \| \| Smlt3571 \| \| Smlt3573 \| \| Smlt3574 \| \| Smlt3576 \| \| Smlt3577 \| \| Smlt3578 (bcr) \| \| Smlt3579 (gcvP) \| \| Smlt3580 \| \| Smlt3581 \| \| Smlt3582  Smlt3583 (katE) \| \| Smlt3584 \| \| Smlt3585 \| \| Smlt3586 \| \| Smlt3587 \| \| Smlt3588 \| \| Smlt3589 \| \| Smlt3590 \| \| Smlt3591 \| \| Smlt3592 (purA) \| \| Smlt3593 \| \| Smlt3594 \| \| Smlt3595 \| \| Smlt3596 (hflK) \| \| Smlt3597 \| \| Smlt3598 \| \| Smlt3599 (cbpA) \| \| Smlt3600 \| \| Smlt3601 (bfr) \| \| Smlt3602 (pbpC) \| \| Smlt3603 \| \| Smlt3604 (prpD) \| \| Smlt3605 \| \| Smlt3606 \| \| Smlt3607 \| \| Smlt3608 (acnA) \| \| Smlt3609 (prpC) \| \| Smlt3610 (prpB) \| \| Smlt3611 \| \| Smlt3612 \| \| Smlt3613 \| \| Smlt3614 \| \| Smlt3615 \| \| Smlt3616 (pdxH) \| \| Smlt3617 \| \| Smlt3618 \| \| Smlt3619 (aroK) \| \| Smlt3620 (aroB) \| \| Smlt3621 \| \| Smlt3622 (hemE) \| \| Smlt3623 \| \| Smlt3625 \| \| Smlt3626 \| \| Smlt3627 \| \| Smlt3628 \| \| Smlt3629 \| \| Smlt3630 \| \| Smlt3631 (sdaA) \| \| Smlt3632 \| \| Smlt3633 (thrA) \| \| Smlt3634 (metB) \| \| Smlt3635 (met2) \| \| Smlt3636 \| \| Smlt3637 (prfC) \| \| Smlt3638 \| \| Smlt3639 \| \| Smlt3640 \| \| Smlt3641 (gtrB) \| \| Smlt3642 (mtgA) \| \| Smlt3643 \| \| Smlt3644 \| \| Smlt3645 \| \| Smlt3646 \| \| Smlt3647 (bpoA) \| \| Smlt3648 (fade) \| \| Smlt3649 \| \| Smlt3650 \| \| Smlt3651 \| \| Smlt3652 \| \| Smlt3653 \| \| Smlt3654 \| \| Smlt3655 \| \| Smlt3656 \| \| Smlt3657 (gcvH) \| \| Smlt3658 (gcvT) \| \| Smlt3659 \| \| Smlt3660 \| \| Smlt3661 \| \| Smlt3662 \| \| Smlt3663 (mazG) \| \| Smlt3654 (cysQ) \| \| Smlt3655 (nude) \| \| Smlt3656 (bioA) \| \| Smlt3667 \| \| Smlt3668 \| \| Smlt3669 \| \| Smlt3670 (frzE) \| \| Smlt3671 (pilJ) \| \| Smlt3672 (pilI) \| \| Smlt3673 (pilH) \| \| Smlt3674 (pilG) \| \| Smlt3675 (gshB) \| \| Smlt3676 \| \| Smlt3677 \| \| Smlt3678 \| \| Smlt3680 \| \| Smlt3681 (mrcB) \| \| Smlt3682 \| \| Smlt3683 (wbpV) \| \| Smlt3684 \| \| Smlt3685 (relA) \| \| Smlt3686 \| \| Smlt3687 (hrpA) \| \| Smlt3688 \| \| Smlt3689 (recQ) \| \| Smlt3690 (copL2) \| \| Smlt3691 (copA2) \| \| Smlt3692 (copB2) \| \| Smlt3693 \| \| Smlt3694 \| \| Smlt3695 \| \| Smlt3696 \| \| Smlt3697 \| \| Smlt3698 \| \| Smlt_t59 \| \| Smlt3699 \| \| Smlt3700 \| \| Smlt3701 \| \| Smlt3702 \| \| Smlt3703 \| \| Smlt3704 (tolB) \| \| Smlt3705 (tolA) \| \| Smlt3706 (tolR) \| \| Smlt3707 (tolQ) \| \| Smlt3708 \| \| Smlt3709 (ruvB) \| \| Smlt3710 (kup) \| \| Smlt3711 (ruvA) \| \| Smlt3712 (ruvC) \| \| Smlt3713 \| \| Smlt3715 \| \| Smlt3716 \| \| Smlt3718(asps) \| \| Smlt3719 \| \| Smlt3720 \| \| Smlt3721 \| \| Smlt3722 \| \| Smlt3723 \| \| Smlt3724 \| \| Smlt3725 \| \| Smlt3726 \| \| Smlt3728 \| \| Smlt3729 \| \| Smlt3730 \| \| Smlt3731 \| \| Smlt3732 (clpB) \| \| Smlt3733 \| \| Smlt3734 \| \| Smlt3735 \| \| Smlt3737(poxB) \| \| Smlt3738 \| \| Smlt3739 \| \| Smlt3740 \| \| Smlt3741 (otsB) \| \| Smlt3742 \| \| Smlt3743 (otsA) \| \| Smlt3744 \| \| Smlt3745 \| \| Smlt3746 \| \| Smlt3747 (rluD) \| \| Smlt3748 (comL) \| \| Smlt3749 \| \| Smlt3751(nadE) \| \| Smlt3752(sucD) \| \| Smlt3753(sucC) \| \| Smlt3754(pilS) \| \| Smlt3755(hydG) \| \| Smlt3756(pilF) \| \| Smlt3757(ppdD) \| \| Smlt3758(pilE) \| \| Smlt3759(pilG) \| \| Smlt3760 \| \| Smlt3761(coaE) \| \| Smlt3763 \| \| Smlt3764 \| \| Smlt3765 \| \| Smlt3766 \| \| Smlt3767 \| \| Smlt3768 \| \| Smlt3769 (rimK) \| \| Smlt3770 \| \| Smlt3771 \| \| Smlt3772 \| \| Smlt_t60 \| \| Smlt3773 \| \| Smlt3774 (this) \| \| Smlt3775 (thiG) \| \| Smlt3776 (trmB) \| \| Smlt3777 \| \| Smlt3778 \| \| Smlt3779 \| \| Smlt3780 \| \| Smlt3781 \| \| Smlt3782 (mscL) \| \| Smlt3783 \| \| Smlt3784 \| \| Smlt3785 \| \| Smlt3786 \| \| Smlt3787 (smeN) \| \| Smlt3788 (smeM) \| \| Smlt3789 \| \| Smlt3790 \| \| Smlt3793(cysB) \| \| Smlt3794(cysK1) \| \| Smlt3795 \| \| Smlt3796 \| \| Smlt3797 (alf1) \| \| Smlt3798 (pykA) \| \| Smlt3799 \| \| Smlt3800(pgk) \| \| Smlt3802 \| \| Smlt3803 \| \| Smlt3804 (gap) \| \| Smlt3805 \| \| Smlt3806 \| \| Smlt3807 \| \| Smlt3808 \| \| Smlt3809 \| \| Smlt3810 (blaI) \| \| Smlt3811 \| \| Smlt3812 \| \| Smlt3813 (tktA) \| \| Smlt3814 \| \| Smlt3815 \| \| Smlt3816 \| \| Smlt3817 \| \| Smlt3818 \| \| Smlt3819 \| \| Smlt3820 \| \| Smlt3821 (pilQ) \| \| Smlt3822 (pilP) \| \| Smlt3823 (pilO) \| \| Smlt3824 (pilN) \| \| Smlt3825 (pilM) \| \| Smlt3826 (mrcA) \| \| Smlt3827 \| \| Smlt3828 \| \| Smlt3829 \| \| Smlt3830 (cblB) \| \| Smlt3831 (cblD) \| \| Smlt3832 (cblC) \| \| Smlt3833 (cblA) \| \| Smlt3834 \| \| Smlt3835 (gltA) \| \| Smlt3836 (rpmE2) \| \| Smlt3837 \| \| Smlt3838 (recG) \| \| Smlt3839 \| \| Smlt3840 (spot) \| \| Smlt3841 \| \| Smlt3842(gmk) \| \| Smlt3843a(fdnG) \| \| Smlt3845(fdnH) \| \| Smlt3846(fdnI) \| \| Smlt3848(fdhE) \| \| Smlt3849(selA) \| \| Smlt3850(selB) \| \| Smlt_t61 \| \| Smlt3851(selD) \| \| Smlt3852 \| \| Smlt3853 \| \| Smlt3854 \| \| Smlt3855 (rph) \| \| Smlt3856 \| \| Smlt3857 \| \| Smlt3858 \| \| Smlt3859 \| \| Smlt3860 \| \| Smlt3861 (pepQ) \| \| Smlt3862 \| \| Smlt3863 \| \| Smlt3864 \| \| Smlt3865 \| \| Smlt3866 \| \| Smlt3867 \| \| Smlt3868 (rpiA) \| \| Smlt3869 \| \| Smlt3870 \| \| Smlt3871(rubA) \| \| Smlt3872(thiE) \| \| Smlt3873(hemL) \| \| Smlt3873A \| \| Smlt3875 \| \| Smlt3876 (argD) \| \| Smlt3877 \| \| Smlt3878 \| \| Smlt3879 \| \| Smlt3880 \| \| Smlt3881 \| \| Smlt3882 \| \| Smlt3883 \| \| Smlt3884 \| \| Smlt3885 (mpl) \| \| Smlt3886 (adk) \| \| Smlt3887 \| \| Smlt3888 \| \| Smlt3889 \| \| Smlt3891(hppA) \| \| Smlt3892 \| \| Smlt3893 \| \| Smlt3894 \| \| Smlt3895 \| \| Smlt3896 (pigA) \| \| Smlt3897 \| \| Smlt3898 \| \| Smlt3899 \| \| Smlt3900(rpoE) \| \| Smlt3900A \| \| Smlt3902(Ppa) \| \| Smlt3903 \| \| Smlt3904 \| \| Smlt3905 \| \| Smlt3906 \| \| Smlt3907 \| \| Smlt3908 \| \| Smlt3909 (thiC) \| \| Smlt3910 (opuD2) \| \| Smlt3911 \| \| Smlt3912 \| \| Smlt3913 \| \| Smlt3914 (ilvG) \| \| Smlt3915 (ilvN) \| \| Smlt3916 (tdcB) \| \| Smlt3917 (leuA) \| \| Smlt3918 (tam) \| \| Smlt3919 (leuC) \| \| Smlt3920 (leuD) \| \| Smlt3921 (leuB) \| \| Smlt3922 \| \| Smlt3923 \| \| Smlt3924 (smeP) \| \| Smlt3925 (smeO) \| \| Smlt3926 \| \| Smlt3927 (pcm) \| \| 928 (tolC) \| \| Smlt3929 \| \| Smlt3930 (kdtA) \| \| Smlt3931 (htrB) \| \| Smlt3932 \| \| Smlt3933 \| \| Smlt3934 \| \| Smlt3935 \| \| Smlt3936 \| \| Smlt3938 \| \| Smlt3940(maeB) \| \| Smlt3942(dctA) \| \| Smlt3943(oprP) \| \| Smlt3944 \| \| Smlt3946 \| \| Smlt3947 \| \| Smlt3948 \| \| Smlt3949 \| \| Smlt3950 \| \| Smlt3951 (citM) \| \| Smlt3952 (fabG) \| \| Smlt3953 \| \| Smlt3954 \| \| Smlt3955 \| \| Smlt3956 \| \| Smlt3957 \| \| Smlt3958 \| \| Smlt3959 \| \| Smlt3960 \| \| Smlt3961 \| \| Smlt3962 \| \| Smlt3963 \| \| Smlt3964 \| \| Smlt3965 \| \| Smlt3966 \| \| Smlt3967 \| \| Smlt3968 \| \| Smlt3969 \| \| Smlt3970 \| \| Smlt3971 \| \| Smlt3972 \| \| Smlt3973 \| \| Smlt3974 \| \| Smlt3975 \| \| Smlt3976 \| \| Smlt3977 \| \| Smlt3978 (adhC) \| \| Smlt3979 \| \| Smlt3980 \| \| Smlt3981 \| \| Smlt3982 \| \| Smlt3983 \| \| Smlt3984 \| \| Smlt3985 \| \| Smlt3986 \| \| Smlt3987 \| \| Smlt3988 \| \| Smlt3989 \| \| Smlt3990 \| \| Smlt3991 \| \| Smlt3992(engB) \| \| Smlt3992A \| \| Smlt3993(dsbA) \| \| Smlt3994 \| \| Smlt3995 \| \| Smlt3996 \| \| Smlt3997 \| \| Smlt3998 \| \| Smlt3999(fhuE) \| \| Smlt3999A \| \| Smlt4003 \| \| Smlt4005 \| \| Smlt4006 \| \| Smlt4007 \| \| Smlt4008 (cca) \| \| Smlt4009 \| \| Smlt4010 \| \| Smlt4011 \| \| Smlt4012 \| \| Smlt4013 \| \| Smlt4014 \| \| Smlt4016 \| \| Smlt4017 \| \| Smlt4018 \| \| Smlt4019 \| \| Smlt4020 (nagA) \| \| Smlt4021 \| \| Smlt4022 \| \| Smlt4023 \| \| Smlt4025 \| \| Smlt4026 \| \| Smlt4027 (nahB) \| \| Smlt4028 \| \| Smlt4029 \| \| Smlt4030 \| \| Smlt4031 \| \| Smlt4032 \| \| Smlt4033 \| \| Smlt4037 \| \| Smlt4038 \| \| Smlt4039 \| \| Smlt4040 \| \| Smlt4041 \| \| Smlt4042 \| \| Smlt4043 \| \| Smlt4044 \| \| Smlt4045 \| \| Smlt4046 (lipA) \| \| Smlt4047 (lipB) \| \| Smlt4048 \| \| Smlt4049 \| \| Smlt4050(dacC) \| \| Smlt4051(rlpA) \| \| Smlt4052(mltB) \| \| Smlt4054(rodA) \| \| Smlt4055(mrdB) \| \| Smlt4056(mrdA) \| \| Smlt4057(mreD) \| \| Smlt4058(mreC) \| \| Smlt4059(mreB) \| \| Smlt4060 \| \| Smlt4061 \| \| Smlt4062 \| \| Smlt4063 \| \| Smlt4064 \| \| Smlt4065 (ubiE) \| \| Smlt4066 \| \| Smlt4067 \| \| Smlt4068 \| \| Smlt4069 \| \| Smlt4070 (smeF) \| \| Smlt4071 (acrB) \| \| Smlt4072 (acrA) \| \| Smlt4073 (smeT) \| \| Smlt4074 \| \| Smlt4075 (hslU) \| \| Smlt4076 (hslV) \| \| Smlt4077 (xerC) \| \| Smlt4078 \| \| Smlt4079 (dapF) \| \| Smlt4080 \| \| Smlt4081 \| \| Smlt4082 (ptrB) \| \| Smlt4083 \| \| Smlt4084 \| \| Smlt4085 \| \| Smlt4086 \| \| Smlt4087 \| \| Smlt4088 (metN) \| \| Smlt4089 \| \| Smlt4090 \| \| Smlt4091 \| \| Smlt4092 \| \| Smlt4093 (prlC) \| \| Smlt4094 \| \| Smlt4095 \| \| Smlt4096 \| \| Smlt4097 \| \| Smlt4098 \| \| Smlt4099 (glmS) \| \| Smlt4100 \| \| Smlt4101 \| \| Smlt4102 \| \| Smlt4103 \| \| Smlt4104 \| \| Smlt4105 \| \| Smlt4106 \| \| Smlt4107 \| \| Smlt4108 (glmU) \| \| Smlt4109 \| \| Smlt4110 (atpC) \| \| Smlt4111 (atpD) \| \| Smlt4112 (atpG) \| \| Smlt4113 (atpA) \| \| Smlt4114 (atpH) \| \| Smlt4115 (atpF) \| \| Smlt4116 (atpC) \| \| Smlt4117 \| \| Smlt4118 \| \| Smlt4119 \| \| Smlt4120 (lpdA) \| \| Smlt4121 (pdhB) \| \| Smlt4122 \| \| Smlt4123 \| \| Smlt4124 (glpD) \| \| Smlt4125 (glpK) \| \| Smlt4126 \| \| Smlt4127 \| \| Smlt4128 \| \| Smlt4129 (hemC) \| \| Smlt4130 \| \| Smlt4131 \| \| Smlt4132(estB) \| \| Smlt4133  Smlt4134 \| \| Smlt4135 (cirA) \| \| Smlt4136 \| \| Smlt4137 \| \| Smlt4138 \| \| Smlt4139 \| \| Smlt4140 \| \| Smlt4141 \| \| Smlt4142 \| \| Smlt4143 \| \| Smlt4144 \| \| Smlt4145 \| \| Smlt4146 \| \| Smlt4147 \| \| Smlt4148 \| \| Smlt4149 \| \| Smlt4150 \| \| Smlt4151 \| \| Smlt4152 \| \| Smlt4153 \| \| Smlt4154 \| \| Smlt4155 \| \| Smlt4156 \| \| Smlt4157 (hns1) \| \| Smlt4158 \| \| Smlt4159 \| \| Smlt4160 \| \| Smlt4161 \| \| Smlt4162 \| \| Smlt_t62 \| \| Smlt4163 \| \| Smlt4164 \| \| Smlt4165 (rpoD) \| \| Smlt4166 (dtd) \| \| Smlt4167 (htrB) \| \| Smlt4168 \| \| Smlt4169 \| \| Smlt4170 \| \| Smlt4171 \| \| Smlt4172 (waaE) \| \| Smlt4173 \| \| Smlt4174 \| \| Smlt4175 \| \| Smlt4176 (sun) \| \| Smlt4177 (fmt) \| \| Smlt4178 (def) \| \| Smlt4179 \| \| Smlt4180 (smf) \| \| Smlt4181 (smg) \| \| Smlt4182 \| \| Smlt4183 \| \| Smlt4184 (topA) \| \| Smlt4185 \| \| Smlt4186 \| \| Smlt4187 \| \| Smlt4188 \| \| Smlt4189 \| \| Smlt4190 (spa) \| \| Smlt4191 \| \| Smlt4192 \| \| Smlt4193 \| \| Smlt4194 (priA) \| \| Smlt4195 \| \| Smlt4196 \| \| Smlt4197 (ndh) \| \| Smlt4198 \| \| Smlt4199 \| \| Smlt4200 \| \| Smlt4201 \| \| Smlt4202 \| \| Smlt4203 (aroG) \| \| Smlt4204 \| \| Smlt4205 \| \| Smlt4206 \| \| Smlt4207 \| \| Smlt4208 \| \| Smlt4209 \| \| Smlt4210 \| \| Smlt4211 \| \| Smlt4213 \| \| Smlt4214 (groEL) \| \| Smlt4215 (groES) \| \| Smlt4216 \| \| Smlt4217 (cutA) \| \| Smlt4218 \| \| Smlt4219 \| \| Smlt4220 \| \| Smlt4221 \| \| Smlt4222 \| \| Smlt4223 \| \| Smlt4224 \| \| Smlt4225 \| \| Smlt4226 \| \| Smlt4227 \| \| Smlt4228 \| \| Smlt4229 \| \| Smlt4231 \| \| Smlt4232 (phoA) \| \| Smlt4233 \| \| Smlt4234 \| \| Smlt4235 \| \| Smlt4236 \| \| Smlt4237 \| \| Smlt4238 \| \| Smlt4239 (aroQ1) \| \| Smlt4240 (accB) \| \| Smlt4241 (accC) \| \| Smlt4242 \| \| Smlt4243 \| \| Smlt4244 (prmA) \| \| Smlt4245 \| \| Smlt4246 (fis) \| \| Smlt4247 \| \| Smlt4248 \| \| Smlt4249 \| \| Smlt4250 \| \| Smlt4251 \| \| Smlt4252 \| \| Smlt4253 (purH) \| \| Smlt4254 (purD) \| \| Smlt4255 \| \| Smlt4256 \| \| Smlt4258(rpoH) \| \| Smlt4259(ung) \| \| Smlt4260 \| \| Smlt4261 (ftsX) \| \| Smlt4262 (ftsE) \| \| Smlt4263 \| \| Smlt4264 (trxA) \| \| Smlt4265 (rho) \| \| Smlt4266 \| \| Smlt4267 \| \| Smlt4268 (aceK) \| \| Smlt4269 \| \| Smlt4270 \| \| Smlt4271 \| \| Smlt4272 \| \| Smlt4273(icd) \| \| Smlt4275 \| \| Smlt4276 \| \| Smlt4277 (queF) \| \| Smlt4278 \| \| Smlt4279 \| \| Smlt4280 \| \| Smlt4281 \| \| Smlt4282 \| \| Smlt4283 \| \| Smlt4284 \| \| Smlt4285 \| \| Smlt4286 \| \| Smlt4287 \| \| Smlt4289 \| \| Smlt4290 (nudC) \| \| Smlt4291 \| \| Smlt4292 \| \| Smlt4293 \| \| Smlt4294 \| \| Smlt4295 \| \| Smlt4296 \| \| Smlt4297 (bfr) \| \| Smlt4298 \| \| Smlt4299(nudH) \| \| Smlt_t63 \| \| Smlt_t64 \| \| Smlt4301(rpsI) \| \| Smlt4302(rplM) \| \| Smlt4303 \| \| Smlt4304 (sugE) \| \| Smlt4305 (speD) \| \| Smlt4306 (cap) \| \| Smlt4307 \| \| Smlt4308 \| \| Smlt4309 (trpC) \| \| Smlt4310 (trpD) \| \| Smlt4311 (pabA) \| \| Smlt4312 \| \| Smlt4313 (trpE) \| \| Smlt4314 \| \| Smlt4315 \| \| Smlt4316 (rpe) \| \| Smlt4317 \| \| Smlt4318 (purC) \| \| Smlt4319 \| \| Smlt4320 (phaAB) \| \| Smlt4321 (phaC) \| \| Smlt4322 (phaD) \| \| Smlt4323 \| \| Smlt4324 (phaF) \| \| Smlt4325 (phaG) \| \| Smlt4326 (yrbG) \| \| Smlt4327 \| \| Smlt4328 \| \| Smlt4329 (hmgA) \| \| Smlt4330 \| \| Smlt4331 \| \| Smlt4332 \| \| Smlt4333 \| \| Smlt4334 \| \| Smlt4335 \| \| Smlt4336 \| \| Smlt4338(pdhA) \| \| Smlt4339 \| \| Smlt4340 \| \| Smlt4341 \| \| Smlt4342 (ehpR) \| \| Smlt4343 \| \| Smlt4344 \| \| Smlt4345 \| \| Smlt4346 \| \| Smlt4347 \| \| Smlt4348(mdoB) \| \| Smlt4350 \| \| Smlt4352 \| \| Smlt4353 \| \| Smlt4354 \| \| Smlt4356 \| \| Smlt4358 \| \| Smlt4359 \| \| Smlt4360 \| \| Smlt4361 \| \| Smlt4362 \| \| Smlt4363 \| \| Smlt4364 (speA) \| \| Smlt4365 (speE) \| \| Smlt4366 \| \| Smlt4368 \| \| Smlt4369 \| \| Smlt4370 (glnB) \| \| Smlt4371 \| \| Smlt4372 \| \| Smlt4373 \| \| Smlt4374 \| \| Smlt4375 \| \| Smlt4376 \| \| Smlt4378 \| \| Smlt4380 \| \| Smlt4381 \| \| Smlt4382 \| \| Smlt4383 \| \| Smlt4384 \| \| Smlt4385 \| \| Smlt4386 \| \| Smlt4387 \| \| Smlt4389 \| \| Smlt4390 \| \| Smlt4391(exoD) \| \| Smlt4392(sndH) \| \| Smlt4392A \| \| Smlt4394 \| \| Smlt4395 \| \| Smlt4396 \| \| Smlt4397 \| \| Smlt4398 \| \| Smlt4399 (cyoC) \| \| Smlt4400 (cyoB) \| \| Smlt4401 (cyoA) \| \| Smlt4402 \| \| Smlt4403 \| \| Smlt4404 \| \| Smlt4405 \| \| Smlt4409 \| \| Smlt4410 \| \| Smlt4411 \| \| Smlt4412 \| \| Smlt4413 \| \| Smlt4414 \| \| Smlt4415 \| \| Smlt4416 \| \| Smlt4417 \| \| Smlt4418 \| \| Smlt4420 \| \| Smlt4422(ubiA) \| \| Smlt_t65 \| \| Smlt4423(afaD) \| \| Smlt4424 \| \| Smlt4426 \| \| Smlt4428 \| \| Smlt4429 \| \| Smlt4430 \| \| Smlt4431 \| \| Smlt4432 \| \| Smlt4433 \| \| Smlt4434 (agaS) \| \| Smlt4435 \| \| Smlt4436 (agaZ) \| \| Smlt4437 (agar) \| \| Smlt4438 (nagA) \| \| Smlt4439 \| \| Smlt4440 \| \| Smlt4441 \| \| Smlt4443 \| \| Smlt4444A \| \| Smlt4451 \| \| Smlt4447A \| \| Smlt4447B \| \| Smlt4447C \| \| Smlt4450 \| \| Smlt4452 \| \| Smlt4453 \| \| Smlt4454 \| \| Smlt4455 \| \| Smlt4458 \| \| Smlt4459 (bioB) \| \| Smlt4460 (bioF) \| \| Smlt4461 (bioH) \| \| Smlt4462 \| \| Smlt4463 \| \| Smlt4469 \| \| Smlt4470 \| \| Smlt4471 \| \| Smlt4472 \| \| Smlt4473 (gidA) \| \| Smlt4474 (oprM) \| \| Smlt4475 (smeB) \| \| Smlt4476 (acrA) \| \| Smlt4477 (smeS) \| \| Smlt4478 (smeR) \| \| Smlt4479 \| \| Smlt4480 \| \| Smlt4481 \| \| Smlt4482 \| \| Smlt4483 \| \| Smlt4484 \| \| Smlt4485 \| \| Smlt4486 \| \| Smlt4487 \| \| Smlt4489 \| \| Smlt4490 \| \| Smlt4491 \| \| Smlt4492 (ilvD) \| \| Smlt4493 (dinG) \| \| Smlt4494 \| \| Smlt4495(aroE) \| \| Smlt4495A \| \| Smlt4496 \| \| Smlt4497 (hemB) \| \| Smlt4498 \| \| Smlt4499 \| \| Smlt4500 \| \| Smlt4501 \| \| Smlt4502 \| \| Smlt4503 \| \| Smlt4504 \| \| Smlt4505 \| \| Smlt4506 \| \| Smlt4507 \| \| Smlt4508 \| \| Smlt4509 \| \| Smlt4510 \| \| Smlt4511 \| \| Smlt4512 \| \| Smlt4513 \| \| Smlt4514 \| \| Smlt4515 (cbpD1) \| \| Smlt4516 (cbpD2) \| \| Smlt4517 \| \| Smlt4518 \| \| Smlt4519 \| \| Smlt4520 \| \| Smlt4521 \| \| Smlt4522 \| \| Smlt4523 (tatD) \| \| Smlt4524 (hrpB) \| \| Smlt4525 \| \| Smlt4526 \| \| Smlt4527 \| \| Smlt4528 \| \| Smlt4529 \| \| Smlt_t67 \| \| Smlt4532 \| \| Smlt4533 \| \| Smlt4534 \| \| Smlt4535 \| \| Smlt4536 \| \| Smlt4537 \| \| Smlt4538 \| \| Smlt4539 \| \| Smlt4540 \| \| Smlt4541 \| \| Smlt4543 \| \| Smlt4544 \| \| Smlt4545 \| \| Smlt4546 \| \| Smlt4547 \| \| Smlt4548 \| \| Smlt4549 \| \| Smlt4550 \| \| Smlt4551 \| \| Smlt4552 \| \| Smlt4553 \| \| Smlt4554 \| \| Smlt4555 \| \| Smlt4556 \| \| Smlt4557 (fabG) \| \| Smlt4558 \| \| Smlt4559 \| \| Smlt4560 \| \| Smlt4561 \| \| Smlt4562 \| \| Smlt4563 \| \| Smlt4564 \| \| Smlt4565 \| \| Smlt4566 \| \| Smlt4567 \| \| Smlt4568 \| \| Smlt4569 \| \| Smlt4570 (acpP) \| \| Smlt4571 \| \| Smlt4572 \| \| Smlt4573 \| \| Smlt4574 (cioB) \| \| Smlt4575 \| \| Smlt4576 \| \| Smlt4577 \| \| Smlt4578 \| \| Smlt4579 \| \| Smlt4580 \| \| Smlt4581 \| \| Smlt4582 \| \| Smlt4583 \| \| Smlt4584 \| \| Smlt4585 \| \| Smlt4586 (hemF) \| \| Smlt4587 \| \| Smlt4588 \| \| Smlt4589 (polA) \| \| Smlt4590 \| \| Smlt4591 \| \| Smlt4592 \| \| Smlt4593 \| \| Smlt4594 \| \| Smlt4595 (uvrD) \| \| Smlt4596 \| \| Smlt4597(cls) \| \| Smlt4598(rpmG) \| \| Smlt4599(rpmB) \| \| Smlt4601 \| \| Smlt4602 \| \| Smlt4603 \| \| Smlt4604 \| \| Smlt4605 \| \| Smlt4606 (smmO) \| \| Smlt4607 (smmP) \| \| Smlt4608 (smmQ) \| \| Smlt4609 \| \| Smlt4610 \| \| Smlt4611 \| \| Smlt4612 \| \| Smlt4613 \| \| Smlt4614 (parB) \| \| Smlt4615 (parA) \| \| Smlt4616 (gidB) \| \| Smlt4617 (hetI) \| \| Smlt4618 \| \| Smlt4619 \| \| Smlt4620 (calB) \| \| Smlt4621 \| \| Smlt4622 \| \| Smlt4623 (acsA) \| \| Smlt4624 \| \| Smlt4626 \| \| Smlt4627 \| \| Smlt4628 \| \| Smlt4629 (hscC) \| \| Smlt4630 \| \| Smlt4631 \| \| Smlt4632 \| \| Smlt4633 \| \| Smlt4634 (glyS) \| \| Smlt4635 (glyQ) \| \| Smlt4636 \| \| Smlt4637 \| \| Smlt4638 \| \| Smlt4639 (tatC) \| \| Smlt4640 (tatB) \| \| Smlt4641 (tatA) \| \| Smlt4642 \| \| Smlt4643 (hemH) \| \| Smlt4644 \| \| Smlt4645 \| \| Smlt_r11 \| \| Smlt_r03 \| \| Smlt_t68 \| \| Smlt_t69 \| \| Smlt_r09 \| \| Smlt_r07 \| \| Smlt_r08 \| \| Smlt_t70 \| \| Smlt_t71 \| \| Smlt_r02 \| \| Smlt4647 \| \| Smlt4648 \| \| Smlt4649 \| \| Smlt4650 \| \| Smlt4651 \| \| Smlt4652 \| \| Smlt4653 \| \| Smlt4654 \| \| Smlt4655 \| \| Smlt4656 \| \| Smlt4657 \| \| Smlt4658 \| \| Smlt4659 (folE) \| \| Smlt4660 \| \| Smlt4661 \| \| Smlt4662 \| \| Smlt4663 \| \| Smlt4664 \| \| Smlt4665 \| \| Smlt4666 \| \| Smlt4667 (recD) \| \| Smlt4668 (recB) \| \| Smlt4669 (recC) \| \| Smlt4670 \| \| Smlt4671 \| \| Smlt4672 \| \| Smlt4673 \| \| Smlt4674 \| \| Smlt4675 \| \| Smlt4676 (btuE) \| \| Smlt4677 \| \| Smlt4678 \| \| Smlt4679 \| \| Smlt4680 \| \| Smlt4681 \| \| Smlt4682 \| \| Smlt4683 \| \| Smlt4684 \| \| Smlt4685 \| \| Smlt4686 \| \| Smlt4687 \| \| Smlt4688 \| \| Smlt4689 \| \| Smlt4690 \| \| Smlt4691 (trmE) \| \| Smlt4692 \| \| Smlt4693 \| \| Smlt4694 \| \| Smlt4695 (rpmH) \| | \| 239.65 \| \| --- \| \| 422.97 \| \| 32.57 \| \| 124.31 \| \| 464.76 \| \| 134.23 \| \| 300.22 \| \| 1,445.91 \| \| 650.2 \| \| 1,822.24 \| \| 1,232.08 \| \| 1,129.31 \| \| 51.9 \| \| 87.68 \| \| 185.94 \| \| 49.91 \| \| 0 \| \| 0 \| \| 0 \| \| 0 \| \| 0 \| \| 0 \| \| 0 \| \| 0 \| \| 0 \| \| 0 \| \| 0 \| \| 0 \| \| 0 \| \| 0 \| \| 0 \| \| 0 \| \| 0.35 \| \| 0 \| \| 0 \| \| 0 \| \| 0 \| \| 224.25 \| \| 0 \| \| 0 \| \| 0 \| \| 0 \| \| 242.43 \| \| 354.97 \| \| 589.11 \| \| 45.57 \| \| 25.72 \| \| 18.54 \| \| 36.95 \| \| 82.25 \| \| 151.66 \| \| 0 \| \| 0 \| \| 0 \| \| 0 \| \| 0 \| \| 0 \| \| 0 \| \| 0 \| \| 0 \| \| 0 \| \| 0 \| \| 0 \| \| 0 \| \| 0 \| \| 50.94 \| \| 118.79 \| \| 190.9 \| \| 0 \| \| 598.27 \| \| 114.92 \| \| 72.81 \| \| 113.24 \| \| 121.84 \| \| 111.08 \| \| 54.6 \| \| 19.36 \| \| 23.98 \| \| 79.65 \| \| 66.08 \| \| 27.77 \| \| 100.09 \| \| 171 \| \| 167.97 \| \| 106.11 \| \| 151.17 \| \| 213.47 \| \| 135.98 \| \| 148.18 \| \| 263.4 \| \| 179.34 \| \| 103.52 \| \| 162.14 \| \| 151.53 \| \| 398.05 \| \| 142.74 \| \| 72.53 \| \| 26.46 \| \| 46.81 \| \| 76.55 \| \| 30.12 \| \| 38.53 \| \| 23.06 \| \| 68.7 \| \| 53.23 \| \| 46.88 \| \| 60.21 \| \| 62.76 \| \| 17.61 \| \| 71.25 \| \| 130.43 \| \| 144.93 \| \| 113.98 \| \| 48.05 \| \| 42.69 \| \| 14.18 \| \| 30.1 \| \| 53 \| \| 38.66 \| \| 92.26 \| \| 112.3 \| \| 73.89 \| \| 58.94 \| \| 0 \| \| 0 \| \| 112.64 \| \| 162.26 \| \| 86.68 \| \| 177.9 \| \| 272.18 \| \| 85.59 \| \| 84.84 \| \| 90.98 \| \| 172.52 \| \| 100.78 \| \| 253.38 \| \| 156.54 \| \| 45.64 \| \| 657.57 \| \| 76.77 \| \| 288.04 \| \| 40.37 \| \| 53.47 \| \| 54.96 \| \| 114.74 \| \| 151.48 \| \| 37.6 \| \| 75.58 \| \| 49.52 \| \| 297.21 \| \| 730.33 \| \| 95.75 \| \| 189.52 \| \| 366.17 \| \| 88.19 \| \| 139.56 \| \| 67.88 \| \| 141.47 \| \| 158.89 \| \| 260.1 \| \| 652.11 \| \| 373.13 \| \| 29.97 \| \| 95.61 \| \| 25.99 \| \| 54.56 \| \| 44.77 \| \| 43.71 \| \| 51.25 \| \| 0 \| \| 254.41 \| \| 896.94 \| \| 75.07 \| \| 2,742.03 \| \| 88.4 \| \| 62.15 \| \| 227.62 \| \| 39.03 \| \| 44.49 \| \| 65.19 \| \| 65.29 \| \| 248.4 \| \| 255.13 \| \| 423.52 \| \| 116.93 \| \| 54.89 \| \| 45.09 \| \| 237.16 \| \| 150.73 \| \| 174.92 \| \| 176.02 \| \| 276.7 \| \| 182.62 \| \| 369.16 \| \| 73.48 \| \| 43.67 \| \| 121.96 \| \| 64.07 \| \| 9.48 \| \| 115.38 \| \| 156.44 \| \| 150.77 \| \| 0 \| \| 0 \| \| 0 \| \| 0 \| \| 81.01 \| \| 96.34 \| \| 104.43 \| \| 146.33 \| \| 150.99 \| \| 371.94 \| \| 20.69 \| \| 23 \| \| 160.46 \| \| 110.64 \| \| 86.93 \| \| 282.85 \| \| 43.53 \| \| 76.7 \| \| 109.08 \| \| 26.82 \| \| 3.24 \| \| 353.56 \| \| 334.53 \| \| 475.07 \| \| 89.28 \| \| 188.86 \| \| 71.47 \| \| 43.82 \| \| 194.3 \| \| 116.52 \| \| 1,208.64 \| \| 1,077.02 \| \| 53.4 \| \| 56.41 \| \| 72.69 \| \| 81.14 \| \| 136.73 \| \| 75.03 \| \| 133.28 \| \| 117.99 \| \| 144.29 \| \| 18.84 \| \| 143.76 \| \| 358.96 \| \| 239.36 \| \| 69.49 \| \| 2,978.24 \| \| 4,391.71 \| \| 2,492.32 \| \| 1,704.43 \| \| 2,034.16 \| \| 89.43 \| \| 42.13 \| \| 3.63 \| \| 7.15 \| \| 149.03 \| \| 83.91 \| \| 194.7 \| \| 434 \| \| 1,038.81 \| \| 225.87 \| \| 16.9 \| \| 84.83 \| \| 348.05 \| \| 161.08 \| \| 50.03 \| \| 44 \| \| 0 \| \| 0 \| \| 0 \| \| 0 \| \| 0 \| \| 0 \| \| 0 \| \| 0 \| \| 0 \| \| 0 \| \| 0 \| \| 0 \| \| 0 \| \| 0 \| \| 0 \| \| 0 \| \| 0 \| \| 0 \| \| 0 \| \| 0 \| \| 0 \| \| 0 \| \| 0 \| \| 0 \| \| 0 \| \| 0 \| \| 0 \| \| 0 \| \| 0 \| \| 0 \| \| 0 \| \| 0 \| \| 0 \| \| 0 \| \| 0 \| \| 0 \| \| 0 \| \| 0 \| \| 0 \| \| 0 \| \| 0 \| \| 0 \| \| 0 \| \| 0 \| \| 0 \| \| 0 \| \| 0 \| \| 0 \| \| 422.54 \| \| 122.07 \| \| 77.63 \| \| 116.08 \| \| 53.25 \| \| 0 \| \| 901.62 \| \| 276.07 \| \| 108.3 \| \| 28.77 \| \| 291.5 \| \| 55.65 \| \| 122.9 \| \| 287.99 \| \| 31.38 \| \| 141.03 \| \| 0 \| \| 0 \| \| 0 \| \| 0 \| \| 0 \| \| 61.24 \| \| 74.22 \| \| 520.61 \| \| 53.12 \| \| 40.89 \| \| 35.38 \| \| 57.81 \| \| 21.03 \| \| 20.31 \| \| 51.45 \| \| 39.26 \| \| 0 \| \| 1.59 \| \| 37.52 \| \| 39.95 \| \| 98.5 \| \| 422.61 \| \| 250.7 \| \| 0 \| \| 0 \| \| 60.98 \| \| 208.16 \| \| 310.99 \| \| 799.4 \| \| 278.06 \| \| 19.26 \| \| 801.42 \| \| 34.79 \| \| 33.91 \| \| 52.25 \| \| 8,623.54 \| \| 133.2 \| \| 302.55 \| \| 71.92 \| \| 74.66 \| \| 108.3 \| \| 152.54 \| \| 23.83 \| \| 319.78 \| \| 223.16 \| \| 257.1 \| \| 23.09 \| \| 29.01 \| \| 114.8 \| \| 117.05 \| \| 115.09 \| \| 69.42 \| \| 42.67 \| \| 27.27 \| \| 35.23 \| \| 32.66 \| \| 276.03 \| \| 348.59 \| \| 231.81 \| \| 121.68 \| \| 54.44 \| \| 102.15 \| \| 348.79 \| \| 316.17 \| \| 6,045.02 \| \| 0 \| \| 0 \| \| 5,217.65 \| \| 0 \| \| 293 \| \| 0 \| \| 0 \| \| 5,176.85 \| \| 0 \| \| 231.93 \| \| 68.62 \| \| 186.08 \| \| 230.51 \| \| 35.76 \| \| 720.34 \| \| 42.54 \| \| 36.51 \| \| 364.02 \| \| 324.29 \| \| 125.59 \| \| 74.07 \| \| 32.77 \| \| 39.43 \| \| 75.58 \| \| 32.09 \| \| 21.24 \| \| 103.49 \| \| 111.86 \| \| 101.78 \| \| 69.67 \| \| 83.34 \| \| 51.68 \| \| 136.47 \| \| 70.28 \| \| 197.44 \| \| 859.64 \| \| 222.8 \| \| 50.34 \| \| 106.58 \| \| 139 \| \| 84.18 \| \| 79.31 \| \| 128.04 \| \| 61.24 \| \| 20.55 \| \| 37.5 \| \| 55.82 \| \| 50.97 \| \| 49.66 \| \| 53.28 \| \| 44.81 \| \| 46.72 \| \| 35.94 \| \| 33.68 \| \| 122.49 \| \| 53.69 \| \| 115.79 \| \| 286.38 \| \| 121.78 \| \| 365.01 \| \| 300.3 \| \| 64.82 \| \| 89.67 \| \| 108.15 \| \| 79.21 \| \| 92.91 \| \| 4.8 \| \| 51.24 \| \| 97.63 \| \| 308.24 \| \| 21.16 \| \| 58.5 \| \| 102.9 \| \| 106.15 \| \| 1,192.22 \| \| 0 \| \| 0 \| \| 0 \| \| 0 \| \| 70.17 \| \| 8.33 \| \| 1.67 \| \| 126.92 \| \| 200.25 \| \| 71.02 \| \| 64.53 \| \| 0 \| \| 0 \| \| 65.34 \| \| 41.86 \| \| 12.75 \| \| 40.19 \| \| 71.41 \| \| 0 \| \| 0 \| \| 96.57 \| \| 114.15 \| \| 0 \| \| 96.68 \| \| 46.72 \| \| 75.49 \| \| 67.32 \| \| 19.73 \| \| 42.12 \| \| 76.6 \| \| 30.82 \| \| 26.13 \| \| 44.32 \| \| 36.14 \| \| 67.3 \| \| 64.27 \| \| 88 \| \| 71.62 \| \| 19.66 \| \| 29.73 \| \| 40.78 \| \| 19.69 \| \| 54.47 \| \| 162.3 \| \| 303.03 \| \| 383.52 \| \| 327.16 \| \| 571.64 \| \| 64.96 \| \| 35.6 \| \| 23.59 \| \| 99.81 \| \| 86.7 \| \| 78.63 \| \| 25.88 \| \| 26.61 \| \| 285.82 \| \| 75.68 \| \| 45.29 \| \| 25.2 \| \| 30.47 \| \| 119.93 \| \| 123.29 \| \| 117.16 \| \| 9.39 \| \| 61.65 \| \| 448.55 \| \| 59.87 \| \| 97.77 \| \| 125.67 \| \| 125.14 \| \| 66.27 \| \| 367.62 \| \| 304.77 \| \| 318.73 \| \| 123.42 \| \| 176.68 \| \| 61.65 \| \| 145.18 \| \| 121.71 \| \| 199 \| \| 221.5 \| \| 292.47 \| \| 45.94 \| \| 51.51 \| \| 32.42 \| \| 94.66 \| \| 269.01 \| \| 275.87 \| \| 150.81 \| \| 120.74 \| \| 128.39 \| \| 286.41 \| \| 75.71 \| \| 61.65 \| \| 21.05 \| \| 23.2 \| \| 21.83 \| \| 53.43 \| \| 43.28  55.23 \| \| 139.38 \| \| 49.59 \| \| 2.9 \| \| 30.82 \| \| 130.43 \| \| 110.83 \| \| 35.35 \| \| 500.77 \| \| 657.57 \| \| 354.4 \| \| 74.69 \| \| 167.66 \| \| 184.61 \| \| 331.95 \| \| 204.79 \| \| 1,932.14 \| \| 553.61 \| \| 277.8 \| \| 0 \| \| 2.12 \| \| 0 \| \| 0 \| \| 0 \| \| 0 \| \| 0 \| \| 0 \| \| 0 \| \| 0 \| \| 0 \| \| 0 \| \| 0 \| \| 0 \| \| 0 \| \| 0 \| \| 0 \| \| 0 \| \| 0 \| \| 0 \| \| 0 \| \| 0 \| \| 0 \| \| 0 \| \| 0 \| \| 0 \| \| 1,009.99 \| \| 869.01 \| \| 860.61 \| \| 681.45 \| \| 511.74 \| \| 249.86 \| \| 0 \| \| 300.07 \| \| 415.19 \| \| 0 \| \| 584.64 \| \| 640.43 \| \| 148.55 \| \| 43.4 \| \| 155.56 \| \| 113.32 \| \| 85.77 \| \| 104.5 \| \| 69.87 \| \| 523.04 \| \| 415.25 \| \| 0 \| \| 185.39 \| \| 268.96 \| \| 53.67 \| \| 80.22 \| \| 59.4 \| \| 251.69 \| \| 124.16 \| \| 412.99 \| \| 137.98 \| \| 119.62 \| \| 0 \| \| 66.91 \| \| 88.88 \| \| 179.98 \| \| 25.54 \| \| 251.73 \| \| 42.38 \| \| 69.37 \| \| 103.75 \| \| 80.28 \| \| 174.11 \| \| 100.74 \| \| 49.49 \| \| 51.42 \| \| 67.21 \| \| 118.78 \| \| 37.62 \| \| 69.79 \| \| 134.21 \| \| 0 \| \| 14.56 \| \| 59.91 \| \| 149.27 \| \| 133.96 \| \| 34.61 \| \| 19,547.32 \| \| 2,982.42 \| \| 1,783.20 \| \| 599.22 \| \| 0 \| \| 0 \| \| 0 \| \| 0 \| \| 0 \| \| 71.62 \| \| 564.27 \| \| 156.07 \| \| 969.55 \| \| 131.1 \| \| 33.72 \| \| 0 \| \| 79.36 \| \| 160.08 \| \| 0 \| \| 323.49 \| \| 60.46 \| \| 198.22 \| \| 245.25 \| \| 346.96 \| \| 336.75 \| \| 107.93 \| \| 0 \| \| 0 \| \| 0 \| \| 0 \| \| 0 \| \| 14.05 \| \| 57.38 \| \| 15.33 \| \| 36.09 \| \| 212.87 \| \| 97.1 \| \| 121.75 \| \| 82.03 \| \| 586.22 \| \| 416.45 \| \| 117.69 \| \| 222.13 \| \| 207.13 \| \| 191.7 \| \| 174.72 \| \| 175.98 \| \| 210.25 \| \| 394.85 \| \| 344.92 \| \| 198.26 \| \| 470.44 \| \| 1,046.21 \| \| 427.07 \| \| 1,070.20 \| \| 1,056.70 \| \| 497.78 \| \| 127.93 \| \| 61.33 \| \| 76.84 \| \| 27 \| \| 33.72 \| \| 43.46 \| \| 31.68 \| \| 119.98 \| \| 80.67 \| \| 100.66 \| \| 1,847.63 \| \| 120.8 \| \| 63.15 \| \| 144.45 \| \| 630.82 \| \| 26.73 \| \| 18.47 \| \| 48.55 \| \| 0 \| \| 0 \| \| 62.99 \| \| 96.18 \| \| 81.34 \| \| 0 \| \| 0 \| \| 217.87 \| \| 128.71 \| \| 191.83 \| \| 31.28 \| \| 33.17 \| \| 20.34 \| \| 33.25 \| \| 34.83 \| \| 52.51 \| \| 74.55 \| \| 1,086.38 \| \| 96.83 \| \| 119.27 \| \| 76.59 \| \| 201.71 \| \| 31.86 \| \| 0 \| \| 0 \| \| 90.54 \| \| 58.34 \| \| 58.28 \| \| 222.43 \| \| 260.08 \| \| 81.95 \| \| 31.24 \| \| 164.63 \| \| 169.53 \| \| 133.88 \| \| 95.02 \| \| 733.99 \| \| 249.1 \| \| 90.99 \| \| 83.97 \| \| 166.29 \| \| 220.28 \| \| 165.54 \| \| 86.87 \| \| 92.82 \| \| 105.49 \| \| 49.22 \| \| 52.03 \| \| 232.21 \| \| 82.87 \| \| 250.9 \| \| 0 \| \| 0 \| \| 324.66 \| \| 103.2 \| \| 165.16 \| \| 283.04 \| \| 2,127.49 \| \| 81.05 \| \| 391.87 \| \| 57.54 \| \| 30.76 \| \| 62.57 \| \| 191.27 \| \| 121.71 \| \| 207.82 \| \| 178.41 \| \| 193.85 \| \| 104.88 \| \| 64.48 \| \| 45.69 \| \| 69.14 \| \| 127.09 \| \| 117.13 \| \| 316.95 \| \| 540.81 \| \| 78.09 \| \| 336.4 \| \| 35.23 \| \| 95.9 \| \| 84.42 \| \| 293.96 \| \| 117.63 \| \| 81.19 \| \| 134.91 \| \| 159.3 \| \| 194.22 \| \| 218.57 \| \| 304.9 \| \| 0 \| \| 356.78 \| \| 1,993.67 \| \| 176.32 \| \| 276.74 \| \| 0 \| \| 0 \| \| 0 \| \| 0 \| \| 0 \| \| 59.42 \| \| 96.46 \| \| 103.95 \| \| 0 \| \| 0 \| \| 0 \| \| 7,409.19 \| \| 0 \| \| 570.91 \| \| 1,246.80 \| \| 2,303.80 \| \| 2,443.14 \| \| 2,622.94 \| \| 4,966.88 \| \| 1,138.68 \| \| 1,259.85 \| \| 2,148.29 \| \| 3,582.58 \| \| 2,822.14 \| \| 3,151.94 \| \| 3,547.10 \| \| 3,620.44 \| \| 3,654.29 \| \| 1,846.96 \| \| 3,763.62 \| \| 2,071.35 \| \| 1,254.58 \| \| 3,424.08 \| \| 2,036.15 \| \| 839.2 \| \| 2,191.91 \| \| 1,755.20 \| \| 1,595.85 \| \| 3,614.38 \| \| 1,555.69 \| \| 2,346.31 \| \| 2,378.53 \| \| 1,500.44 \| \| 2,463.85 \| \| 643.45 \| \| 1,966.05 \| \| 491.29 \| \| 2,076.33 \| \| 2,151.97 \| \| 3,754.62 \| \| 2,232.27 \| \| 925.68 \| \| 183.12 \| \| 57.04 \| \| 134.72 \| \| 36.05 \| \| 129.22 \| \| 91.68 \| \| 752.18 \| \| 1,670.50 \| \| 2,079.52 \| \| 94.73 \| \| 656.79 \| \| 253.19 \| \| 197.86 \| \| 427.3 \| \| 216.45 \| \| 67.57 \| \| 40.51 \| \| 143.6 \| \| 21,510.76 \| \| 476.4 \| \| 180.51 \| \| 367.68 \| \| 276.27 \| \| 158.9 \| \| 543.44 \| \| 244.2 \| \| 79.71 \| \| 140.16 \| \| 194.51 \| \| 132.74 \| \| 278.11 \| \| 132.26 \| \| 79.79 \| \| 90.85 \| \| 284.72 \| \| 14.38 \| \| 32.28 \| \| 35.15 \| \| 119.18 \| \| 78.3 \| \| 75.25 \| \| 88.99 \| \| 154.39 \| \| 133.06 \| \| 177.88 \| \| 1,292.94 \| \| 18.36 \| \| 41.91 \| \| 0 \| \| 481.48 \| \| 0 \| \| 0 \| \| 0 \| \| 0 \| \| 0 \| \| 0 \| \| 0 \| \| 908.44 \| \| 562.79 \| \| 907.22 \| \| 490.76 \| \| 2,165.11 \| \| 0 \| \| 0 \| \| 0 \| \| 0 \| \| 0 \| \| 418.11 \| \| 220.94 \| \| 363.6 \| \| 223.29 \| \| 127.38 \| \| 213.74 \| \| 113.85 \| \| 0 \| \| 237.87 \| \| 451.17 \| \| 246.94 \| \| 318.1 \| \| 212.39 \| \| 90.1 \| \| 20.74 \| \| 37.24 \| \| 25.83 \| \| 0 \| \| 0 \| \| 0 \| \| 0 \| \| 290.05 \| \| 546.18 \| \| 131.93 \| \| 229.12 \| \| 534.51 \| \| 44.2 \| \| 33.84 \| \| 115.93 \| \| 111.25 \| \| 76.57 \| \| 527.27 \| \| 610.78 \| \| 481.08 \| \| 310.84 \| \| 312.35 \| \| 369.39 \| \| 2,595.36 \| \| 518.37 \| \| 107.58 \| \| 134.44 \| \| 134.96 \| \| 131.41 \| \| 122.25 \| \| 0 \| \| 104.85 \| \| 82.77 \| \| 35.96 \| \| 57.7 \| \| 45.03 \| \| 53.39 \| \| 116.69 \| \| 130.67 \| \| 3.92 \| \| 41.72 \| \| 39.36 \| \| 31.34 \| \| 26.19 \| \| 23.16 \| \| 35.99 \| \| 24.46 \| \| 25.88 \| \| 14.75 \| \| 38.02 \| \| 188.8 \| \| 163.81 \| \| 69.76 \| \| 21.44 \| \| 110 \| \| 491.28 \| \| 274.15 \| \| 204.37 \| \| 57.06 \| \| 48.38 \| \| 41.1 \| \| 99.49 \| \| 96.39 \| \| 36.71 \| \| 26.74 \| \| 93.55 \| \| 64.33 \| \| 48.8 \| \| 69.16 \| \| 35.93 \| \| 42.61 \| \| 81.49 \| \| 64.57 \| \| 38.76 \| \| 167.51 \| \| 62.57 \| \| 53.62 \| \| 238.94 \| \| 99.29 \| \| 444 \| \| 402.57 \| \| 136.83 \| \| 49.73 \| \| 0 \| \| 13.89 \| \| 89.05 \| \| 63 \| \| 76.63 \| \| 376.41 \| \| 99.38 \| \| 267.2 \| \| 0 \| \| 107.68 \| \| 57.57 \| \| 112.22 \| \| 130.14 \| \| 176.94 \| \| 214.83 \| \| 130.3 \| \| 236.79 \| \| 4,040.82 \| \| 165.69 \| \| 173.64 \| \| 269.37 \| \| 96.97 \| \| 232.44 \| \| 164.27 \| \| 166.53 \| \| 324.52 \| \| 120.97 \| \| 223.34 \| \| 158.84 \| \| 70.05 \| \| 45.19 \| \| 52.95 \| \| 256.72 \| \| 208.08 \| \| 153.96 \| \| 128.5 \| \| 30.69 \| \| 60.57 \| \| 86.31 \| \| 496.89 \| \| 19,825.97 \| \| 117.85 \| \| 144.01 \| \| 139.69 \| \| 46.09 \| \| 67.32 \| \| 121.91 \| \| 71.27 \| \| 33.08 \| \| 27.2 \| \| 9.16 \| \| 44.74 \| \| 51.89 \| \| 90.39 \| \| 64.84 \| \| 112.19 \| \| 127.4 \| \| 25 \| \| 72.09 \| \| 80.83 \| \| 95.73 \| \| 74.89 \| \| 128.56 \| \| 108.69 \| \| 262.4 \| \| 86.51 \| \| 209.42 \| \| 112.85 \| \| 138.86 \| \| 8,567.61 \| \| 185.8 \| \| 73.22 \| \| 172.44 \| \| 200.27 \| \| 181.53  131.69 \| \| 95.9 \| \| 97.14 \| \| 395.43 \| \| 39.14 \| \| 32.43 \| \| 22.35 \| \| 43.23 \| \| 36.04 \| \| 43.25 \| \| 102.27 \| \| 70 \| \| 22.74 \| \| 0 \| \| 36.11 \| \| 20.07 \| \| 58.06 \| \| 529.97 \| \| 55.93 \| \| 81.17 \| \| 48.16 \| \| 40.45 \| \| 26.15 \| \| 60.36 \| \| 29.92 \| \| 35.23 \| \| 78.87 \| \| 80.73 \| \| 36.53 \| \| 75.35 \| \| 80.25 \| \| 30.64 \| \| 18.71 \| \| 28.26 \| \| 76.04 \| \| 158.86 \| \| 66.73 \| \| 43.12 \| \| 13.15 \| \| 61.29 \| \| 104.1 \| \| 45.17 \| \| 13.08 \| \| 18.8 \| \| 20.94 \| \| 18.35 \| \| 22.87 \| \| 7.45 \| \| 59.9 \| \| 39.41 \| \| 64.93 \| \| 54.01 \| \| 290.68 \| \| 22.15 \| \| 129.46 \| \| 28.39 \| \| 21.29 \| \| 27.25 \| \| 1.15 \| \| 27.28 \| \| 41.1 \| \| 33.42 \| \| 72.9 \| \| 28.14 \| \| 60.22 \| \| 36.61 \| \| 0 \| \| 42.05 \| \| 89.56 \| \| 57.12 \| \| 80.93 \| \| 24.47 \| \| 32.45 \| \| 184.47 \| \| 373.32 \| \| 46.91 \| \| 79.42 \| \| 202.66 \| \| 724.24 \| \| 345.98 \| \| 379.8 \| \| 50.06 \| \| 125.6 \| \| 1,271.18 \| \| 52.7 \| \| 62.8 \| \| 43.59 \| \| 4.11 \| \| 32.88 \| \| 119.52 \| \| 89.67 \| \| 146.85 \| \| 102.26 \| \| 24.4 \| \| 47.37 \| \| 87.96 \| \| 211.19 \| \| 191.84 \| \| 259 \| \| 114.12 \| \| 188.57 \| \| 254 \| \| 2,207.34 \| \| 1,939.09 \| \| 268.72 \| \| 0 \| \| 6.46 \| \| 5.62 \| \| 1.57 \| \| 3.99 \| \| 4.01 \| \| 8.77 \| \| 1.35 \| \| 4.82 \| \| 10.66 \| \| 5.79 \| \| 12.94 \| \| 0 \| \| 0 \| \| 0 \| \| 0 \| \| 0 \| \| 0 \| \| 0 \| \| 0 \| \| 0 \| \| 0 \| \| 0 \| \| 0 \| \| 0 \| \| 0 \| \| 6.96 \| \| 0 \| \| 6.42 \| \| 8.45 \| \| 5.6 \| \| 13.89 \| \| 7.54 \| \| 7.63 \| \| 2.76 \| \| 0 \| \| 0 \| \| 34.05 \| \| 24.48 \| \| 0 \| \| 0 \| \| 0 \| \| 3.91 \| \| 5.28 \| \| 4.9 \| \| 1.87 \| \| 0 \| \| 0 \| \| 0 \| \| 0 \| \| 0 \| \| 0 \| \| 54.2 \| \| 1,850.79 \| \| 59.82 \| \| 201.72 \| \| 270.62 \| \| 135.27 \| \| 263.7 \| \| 51.24 \| \| 29.44 \| \| 67.3 \| \| 73.05 \| \| 61.85 \| \| 121.77 \| \| 126.08 \| \| 162.2 \| \| 89.07 \| \| 0 \| \| 0 \| \| 0 \| \| 40.87 \| \| 89.8 \| \| 159.12 \| \| 43.96 \| \| 78.57 \| \| 1,178.59 \| \| 1,109.10 \| \| 877.54 \| \| 290.93 \| \| 36.49 \| \| 378.48 \| \| 148.39 \| \| 245.95 \| \| 95.41 \| \| 80.03 \| \| 311.86 \| \| 74.53 \| \| 96.45 \| \| 1,152.17 \| \| 274.71 \| \| 130.6 \| \| 1,728.89 \| \| 84.46 \| \| 109.69 \| \| 79.2 \| \| 48.61 \| \| 74.34 \| \| 84.65 \| \| 203.53 \| \| 169.53 \| \| 94.61 \| \| 0 \| \| 0 \| \| 4.48 \| \| 13.57 \| \| 0 \| \| 0 \| \| 0 \| \| 0 \| \| 0 \| \| 38.1 \| \| 305.73 \| \| 85.59 \| \| 66.01 \| \| 96.3 \| \| 101.76 \| \| 32.58 \| \| 22.85 \| \| 82.04 \| \| 53.83 \| \| 50.35 \| \| 48.48 \| \| 77.45 \| \| 192.2 \| \| 75.83 \| \| 34.13 \| \| 29.66 \| \| 40.07 \| \| 118.73 \| \| 39.67 \| \| 57.57 \| \| 54.33 \| \| 33.79 \| \| 37.09 \| \| 61.48 \| \| 383.58 \| \| 272.98 \| \| 60.83 \| \| 695.88 \| \| 201.37 \| \| 42.49 \| \| 190.23 \| \| 741.12 \| \| 112.95 \| \| 50.77 \| \| 46.08 \| \| 34.19 \| \| 26.1 \| \| 99.38 \| \| 519.79 \| \| 219.53 \| \| 91.59 \| \| 51.93 \| \| 110.68 \| \| 32.16 \| \| 30.38 \| \| 80.14 \| \| 207.27 \| \| 27.51 \| \| 36.09 \| \| 49.77 \| \| 32.64 \| \| 137.91 \| \| 113.81 \| \| 7.1 \| \| 66.39 \| \| 1,174.28 \| \| 96.64 \| \| 398.31 \| \| 43.64 \| \| 77.31 \| \| 158.6 \| \| 198.93 \| \| 184.79 \| \| 88.18 \| \| 35.23 \| \| 44.99 \| \| 101.24 \| \| 109.37 \| \| 68.4 \| \| 28.97 \| \| 221.03 \| \| 115.01 \| \| 120.01 \| \| 36.49 \| \| 36.01 \| \| 36.22 \| \| 0 \| \| 23.79 \| \| 101.44 \| \| 20.21 \| \| 62.39 \| \| 138.57 \| \| 38.18 \| \| 72.19 \| \| 503.58 \| \| 161.5 \| \| 164.7 \| \| 85.87 \| \| 185.82 \| \| 335.79 \| \| 361.02 \| \| 284.92 \| \| 588.76 \| \| 147.25 \| \| 127.33 \| \| 137.88 \| \| 305.83 \| \| 863.06 \| \| 325.74 \| \| 423.72 \| \| 1,349.49 \| \| 1,702.29 \| \| 29.63 \| \| 21.05 \| \| 22.09 \| \| 26.55 \| \| 20.63 \| \| 29.26 \| \| 632.18 \| \| 116.82 \| \| 286.64 \| \| 224.36 \| \| 283.64 \| \| 68.42 \| \| 94.53 \| \| 75.63 \| \| 931.14 \| \| 397.74 \| \| 154.43 \| \| 56.65 \| \| 68.33 \| \| 86.99 \| \| 93.23 \| \| 6.63 \| \| 0 \| \| 28.36 \| \| 20.05 \| \| 0 \| \| 0 \| \| 261.15 \| \| 45.18 \| \| 154.12 \| \| 53.76 \| \| 40.78 \| \| 38.33 \| \| 215.64 \| \| 91.03 \| \| 70.75 \| \| 263.07 \| \| 14.17 \| \| 18.59 \| \| 30.4 \| \| 48.06 \| \| 30.82 \| \| 34.49 \| \| 29.11 \| \| 29.01 \| \| 61.48 \| \| 25.82 \| \| 43.03 \| \| 53.61 \| \| 42.84 \| \| 140.77 \| \| 2,647.15 \| \| 140.77 \| \| 196.62 \| \| 78.93 \| \| 102.28 \| \| 100.3 \| \| 11.21 \| \| 47.42 \| \| 31.76 \| \| 58.05 \| \| 185.57 \| \| 54.13 \| \| 66.22 \| \| 79.38 \| \| 535.79 \| \| 1,398.68 \| \| 88.89 \| \| 65.54 \| \| 70.98 \| \| 53.25 \| \| 111.16 \| \| 152.13 \| \| 440.86 \| \| 75.12 \| \| 141.84 \| \| 54.31 \| \| 49.14 \| \| 145.79 \| \| 94.01 \| \| 96.09 \| \| 309.91 \| \| 60.08 \| \| 35.23 \| \| 88.07 \| \| 246.59 \| \| 235.35 \| \| 112.86 \| \| 72.86 \| \| 69.46 \| \| 168.39 \| \| 30.38 \| \| 103.45 \| \| 95.18 \| \| 312.7 \| \| 179.07 \| \| 157.32 \| \| 388.5 \| \| 273.54 \| \| 97.08 \| \| 78.63 \| \| 214.9 \| \| 282.21 \| \| 360.34 \| \| 267.51 \| \| 169.8 \| \| 166.74 \| \| 246.59 \| \| 147.1 \| \| 1,508.80 \| \| 306.77 \| \| 341.21 \| \| 229.61 \| \| 359.77 \| \| 187.07 \| \| 349.87 \| \| 308.24 \| \| 0 \| \| 53.8 \| \| 182.84 \| \| 0 \| \| 0 \| \| 49.89 \| \| 430.88 \| \| 90.85 \| \| 57.4 \| \| 64.89 \| \| 42.86 \| \| 61.65 \| \| 65.23 \| \| 53.48 \| \| 224.83 \| \| 58.59 \| \| 131.97 \| \| 91.38 \| \| 107.52 \| \| 52.95 \| \| 139.26 \| \| 82 \| \| 154.56 \| \| 0 \| \| 21.38 \| \| 0 \| \| 69.99 \| \| 60.35 \| \| 40.59 \| \| 76.71 \| \| 36.99 \| \| 0 \| \| 0 \| \| 0 \| \| 0 \| \| 2.39 \| \| 34.1 \| \| 14.23 \| \| 18.08 \| \| 0 \| \| 0 \| \| 0 \| \| 0 \| \| 0 \| \| 0 \| \| 0 \| \| 0 \| \| 0 \| \| 0 \| \| 0 \| \| 0 \| \| 278.2 \| \| 100.97 \| \| 70.17 \| \| 130.14 \| \| 69.99 \| \| 41.4 \| \| 34.29 \| \| 137.49 \| \| 80.94 \| \| 139.45 \| \| 40.51 \| \| 146.71 \| \| 247.3 \| \| 382.73 \| \| 270.24 \| \| 1,337.09 \| \| 0 \| \| 633.48 \| \| 206.07 \| \| 226.98 \| \| 93.01 \| \| 64.31 \| \| 79.08 \| \| 196.06 \| \| 214.8 \| \| 116.24 \| \| 31.2 \| \| 58.28 \| \| 199.86 \| \| 364.58 \| \| 299.01 \| \| 39.04 \| \| 39.61 \| \| 282.4 \| \| 926.8 \| \| 392.99 \| \| 518.11 \| \| 130.74 \| \| 81.09 \| \| 779.25 \| \| 370.93 \| \| 166.16 \| \| 104.73 \| \| 110.03 \| \| 61.65 \| \| 413.51 \| \| 183.85 \| \| 213.51 \| \| 431.08 \| \| 144.95 \| \| 55.24 \| \| 338.46 \| \| 253.02 \| \| 795.41 \| \| 51.7 \| \| 29.18 \| \| 0 \| \| 163.71 \| \| 63.97 \| \| 519.98 \| \| 362.27 \| \| 160.95 \| \| 83.74 \| \| 205.31 \| \| 2,097.44 \| \| 426.27 \| \| 469.72 \| \| 103.35 \| \| 0 \| \| 45.97 \| \| 82.51 \| \| 213.66 \| \| 49.26 \| \| 65.56 \| \| 20.23 \| \| 23.14 \| \| 31.11 \| \| 30.59 \| \| 66.27 \| \| 85.51 \| \| 711.44 \| \| 766.13 \| \| 34.71 \| \| 43.15 \| \| 30.82 \| \| 27.68 \| \| 27.93 \| \| 78.39 \| \| 0 \| \| 0 \| \| 0 \| \| 71.82 \| \| 145.21  51.42 \| \| 164.88 \| \| 297.28 \| \| 389.15 \| \| 305.24 \| \| 337.2 \| \| 981.34 \| \| 204.35 \| \| 71.64 \| \| 0 \| \| 202.82 \| \| 101.98 \| \| 301.89 \| \| 222.81 \| \| 37.2 \| \| 286.39 \| \| 279.79 \| \| 343 \| \| 116.96 \| \| 350.6 \| \| 97.65 \| \| 255.15 \| \| 204.56 \| \| 119.63 \| \| 91.06 \| \| 136.51 \| \| 173.71 \| \| 73.09 \| \| 143.14 \| \| 622.12 \| \| 393.78 \| \| 1,142.08 \| \| 1,063.07 \| \| 72.79 \| \| 31.85 \| \| 237.66 \| \| 175.1 \| \| 102.34 \| \| 126.03 \| \| 91.73 \| \| 223.56 \| \| 150.42 \| \| 787.25 \| \| 35.77 \| \| 213.23 \| \| 3,044.03 \| \| 305.61 \| \| 279.92 \| \| 240.34 \| \| 53.35 \| \| 91.44 \| \| 154.76 \| \| 218.54 \| \| 39.54 \| \| 30.74 \| \| 28.39 \| \| 30.07 \| \| 22.92 \| \| 50.46 \| \| 735.52 \| \| 114.69 \| \| 4.87 \| \| 26.87 \| \| 32.08 \| \| 20.53 \| \| 22.85 \| \| 22.42 \| \| 25.44 \| \| 50.69 \| \| 81.65 \| \| 0 \| \| 0 \| \| 45.65 \| \| 67.79 \| \| 80.31 \| \| 68.85 \| \| 100.83 \| \| 0 \| \| 0 \| \| 0 \| \| 0 \| \| 0 \| \| 0 \| \| 0 \| \| 0 \| \| 0 \| \| 0 \| \| 0 \| \| 0 \| \| 0 \| \| 0 \| \| 0 \| \| 0 \| \| 0 \| \| 0 \| \| 0 \| \| 0 \| \| 0 \| \| 0 \| \| 0 \| \| 0 \| \| 0 \| \| 0 \| \| 0 \| \| 0 \| \| 0 \| \| 0 \| \| 0 \| \| 0 \| \| 0 \| \| 0 \| \| 0 \| \| 0 \| \| 0 \| \| 0 \| \| 0 \| \| 0 \| \| 0 \| \| 0 \| \| 0 \| \| 0 \| \| 0 \| \| 0 \| \| 0 \| \| 0 \| \| 0 \| \| 0 \| \| 0 \| \| 0 \| \| 0 \| \| 0 \| \| 0 \| \| 0 \| \| 0 \| \| 0 \| \| 0 \| \| 0 \| \| 0 \| \| 0 \| \| 0 \| \| 0 \| \| 0 \| \| 0 \| \| 0 \| \| 0 \| \| 0 \| \| 0 \| \| 0 \| \| 0 \| \| 0 \| \| 0 \| \| 0 \| \| 0 \| \| 0 \| \| 0 \| \| 0 \| \| 0 \| \| 0 \| \| 0 \| \| 0 \| \| 0 \| \| 0 \| \| 0 \| \| 0 \| \| 0 \| \| 0 \| \| 0 \| \| 0 \| \| 0 \| \| 0 \| \| 0 \| \| 0 \| \| 0 \| \| 0 \| \| 0 \| \| 0 \| \| 0 \| \| 0 \| \| 0 \| \| 0 \| \| 0 \| \| 0 \| \| 0 \| \| 0 \| \| 0 \| \| 0 \| \| 0 \| \| 0 \| \| 0 \| \| 0 \| \| 0 \| \| 0 \| \| 0 \| \| 0 \| \| 0 \| \| 0 \| \| 0 \| \| 0 \| \| 0 \| \| 0 \| \| 0 \| \| 0 \| \| 0 \| \| 0 \| \| 0 \| \| 0 \| \| 0 \| \| 0 \| \| 72.3 \| \| 289.89 \| \| 192.76 \| \| 83.15 \| \| 872.48 \| \| 561.17 \| \| 25.42 \| \| 80.99 \| \| 218.55 \| \| 91 \| \| 821.49 \| \| 2,343.37 \| \| 291.96 \| \| 28.62 \| \| 21.58 \| \| 135.5 \| \| 88.77 \| \| 51.21 \| \| 68.22 \| \| 144.36 \| \| 36.01 \| \| 68.85 \| \| 66.59 \| \| 196.77 \| \| 1,009.34 \| \| 105.63 \| \| 138.67 \| \| 709.47 \| \| 534.87 \| \| 463.39 \| \| 554.53 \| \| 160.64 \| \| 70.86 \| \| 160.35 \| \| 34.16 \| \| 68.08 \| \| 660.51 \| \| 37.14 \| \| 103.45 \| \| 140.35 \| \| 122.49 \| \| 27.4 \| \| 46.87 \| \| 203.59 \| \| 238.58 \| \| 280.73 \| \| 41.42 \| \| 33.82 \| \| 34.39 \| \| 164.69 \| \| 114.94 \| \| 367.39 \| \| 284.72 \| \| 264.52 \| \| 61.65 \| \| 241.13 \| \| 3,880.94 \| \| 230.88 \| \| 167.42 \| \| 242.83 \| \| 103.49 \| \| 161.17 \| \| 350.53 \| \| 502.05 \| \| 471.05 \| \| 106.89 \| \| 733.69 \| \| 334 \| \| 131.77 \| \| 343.19 \| \| 772.76 \| \| 825.22 \| \| 253.47 \| \| 24.17 \| \| 18.03 \| \| 37.17 \| \| 92.06 \| \| 0 \| \| 52.87 \| \| 7.25 \| \| 177.16 \| \| 619.01 \| \| 251.79 \| \| 0 \| \| 0 \| \| 0 \| \| 0 \| \| 53.36 \| \| 55.2 \| \| 22.02 \| \| 30.71 \| \| 40.07 \| \| 124.24 \| \| 32.88 \| \| 18.08 \| \| 102.89 \| \| 0 \| \| 124.89 \| \| 69.98 \| \| 128.16 \| \| 76.8 \| \| 252.44 \| \| 86.81 \| \| 17.72 \| \| 1.58 \| \| 27.21 \| \| 16.61 \| \| 23.4 \| \| 15.25 \| \| 16.12 \| \| 23.45 \| \| 19.54 \| \| 23.02 \| \| 10.05 \| \| 25 \| \| 24.21 \| \| 25.01 \| \| 0 \| \| 0.74 \| \| 46.71 \| \| 157.14 \| \| 51.22 \| \| 0 \| \| 0 \| \| 39.21 \| \| 0 \| \| 0 \| \| 0 \| \| 16.1 \| \| 33.86 \| \| 43.81 \| \| 34.31 \| \| 148.52 \| \| 62.64 \| \| 213.85 \| \| 247.29 \| \| 137.69 \| \| 158.65 \| \| 188.02 \| \| 1,100.49 \| \| 0 \| \| 36.71 \| \| 19.89 \| \| 52.09 \| \| 133.25 \| \| 717.07 \| \| 119.34 \| \| 121.24 \| \| 131.1 \| \| 87.76 \| \| 53.8 \| \| 46.24 \| \| 73.06 \| \| 0.85 \| \| 28.81 \| \| 35.47 \| \| 48.25 \| \| 66.39 \| \| 114.39 \| \| 87.84 \| \| 89.46 \| \| 71.17 \| \| 54.17 \| \| 79.04 \| \| 205.82 \| \| 271.51 \| \| 308.8 \| \| 216.98 \| \| 109.88 \| \| 80.06 \| \| 78.87 \| \| 53.98 \| \| 26.67 \| \| 59.98 \| \| 108.4 \| \| 165.67 \| \| 62.3 \| \| 111.36 \| \| 152.17 \| \| 67.71 \| \| 102.16 \| \| 63.13 \| \| 38.12 \| \| 237.11 \| \| 263.77 \| \| 190.49 \| \| 96.8 \| \| 123.29 \| \| 110.3 \| \| 133.85 \| \| 51.13 \| \| 300.8 \| \| 299.62 \| \| 195.31 \| \| 101.14 \| \| 118.13 \| \| 76.67 \| \| 160.88 \| \| 90.22 \| \| 97.85 \| \| 164.3 \| \| 120.83 \| \| 68.95 \| \| 103.72 \| \| 178.09 \| \| 318.24 \| \| 168.5 \| \| 64.94 \| \| 1,227.61 \| \| 486 \| \| 365.09 \| \| 152.98 \| \| 103.05 \| \| 263 \| \| 111.69 \| \| 92.37 \| \| 43.52 \| \| 54.44 \| \| 36.23 \| \| 20.55 \| \| 52.57 \| \| 263.54 \| \| 416.2 \| \| 363.84 \| \| 522.65 \| \| 159.56 \| \| 100.2 \| \| 133.57 \| \| 44.7 \| \| 0 \| \| 6.34 \| \| 618.23 \| \| 93.06 \| \| 75.52 \| \| 95.35 \| \| 675.19 \| \| 128.49 \| \| 54.95 \| \| 101.19 \| \| 602.36 \| \| 208.13 \| \| 148.15 \| \| 152.37 \| \| 33.86 \| \| 506.07 \| \| 134.99 \| \| 182.48 \| \| 163.78 \| \| 944.12 \| \| 50.49 \| \| 27.95 \| \| 48.56 \| \| 34.08 \| \| 44.22 \| \| 78.02 \| \| 0 \| \| 154.86 \| \| 246.59 \| \| 130.81 \| \| 71.21 \| \| 41.59 \| \| 194.97 \| \| 222.34 \| \| 287.29 \| \| 98.94 \| \| 100.62 \| \| 51.28 \| \| 99.83 \| \| 117.23 \| \| 161.14 \| \| 163.77 \| \| 135.23 \| \| 172.86 \| \| 136.06 \| \| 41.39 \| \| 31.4 \| \| 67.46 \| \| 29.96 \| \| 21.98 \| \| 31.51 \| \| 69.23 \| \| 59.01 \| \| 133.2 \| \| 133.23 \| \| 166.3 \| \| 85.8 \| \| 75.08 \| \| 72.32 \| \| 80.08 \| \| 121.45 \| \| 124.87 \| \| 148.56 \| \| 0 \| \| 0 \| \| 19.73 \| \| 4.62 \| \| 340.17 \| \| 233.87 \| \| 275.71 \| \| 153.1 \| \| 61.97 \| \| 35.23 \| \| 103.78 \| \| 259.36 \| \| 1,013.32 \| \| 250.71 \| \| 220.84 \| \| 116.53 \| \| 138.83 \| \| 88.36 \| \| 82.2 \| \| 153.72 \| \| 248.94 \| \| 152.89 \| \| 289.5 \| \| 165.47 \| \| 67.99 \| \| 118.62 \| \| 63.8 \| \| 83.32 \| \| 835.26 \| \| 198.58 \| \| 195.87 \| \| 104.52 \| \| 67.01 \| \| 56.69 \| \| 44.5 \| \| 3.64 \| \| 74.22 \| \| 169.88 \| \| 190.05 \| \| 200.31 \| \| 245.79 \| \| 669.09 \| \| 143.66 \| \| 65.97 \| \| 442.53 \| \| 26.01 \| \| 214.5 \| \| 61.9 \| \| 56.84 \| \| 62.14 \| \| 552.92 \| \| 313.33 \| \| 297.39 \| \| 249.48 \| \| 210.47 \| \| 51.45 \| \| 65.99 \| \| 1.62 \| \| 0 \| \| 23.36 \| \| 21.76 \| \| 32.93 \| \| 17.01 \| \| 14.08 \| \| 145.98 \| \| 8.61 \| \| 124.02 \| \| 166.65 \| \| 480.95 \| \| 0 \| \| 0 \| \| 22.86 \| \| 24.21 \| \| 31.06 \| \| 11.52 \| \| 60.47 \| \| 31.31 \| \| 17.94 \| \| 21.78 \| \| 21.19 \| \| 56.31 \| \| 29.04 \| \| 51.24 \| \| 96.56 \| \| 158.65 \| \| 102.64  41.52 \| \| 32.64 \| \| 46.99 \| \| 184.17 \| \| 0 \| \| 0 \| \| 0 \| \| 0 \| \| 0 \| \| 0 \| \| 0 \| \| 0 \| \| 0 \| \| 0 \| \| 0 \| \| 0 \| \| 0 \| \| 0 \| \| 0 \| \| 0 \| \| 0 \| \| 0 \| \| 0 \| \| 0 \| \| 0 \| \| 2.72 \| \| 2.11 \| \| 0 \| \| 8.73 \| \| 0 \| \| 0 \| \| 0 \| \| 0 \| \| 0 \| \| 0 \| \| 0 \| \| 0 \| \| 0 \| \| 0 \| \| 0 \| \| 0 \| \| 0 \| \| 0 \| \| 0 \| \| 0 \| \| 0 \| \| 0 \| \| 0 \| \| 0 \| \| 0 \| \| 0 \| \| 0 \| \| 0 \| \| 0 \| \| 0 \| \| 0 \| \| 0 \| \| 0 \| \| 0 \| \| 0 \| \| 0 \| \| 0 \| \| 0 \| \| 0 \| \| 0 \| \| 0 \| \| 0 \| \| 0 \| \| 0 \| \| 0 \| \| 0 \| \| 0 \| \| 0 \| \| 0 \| \| 0 \| \| 0 \| \| 0 \| \| 0 \| \| 0 \| \| 0 \| \| 0 \| \| 0 \| \| 0 \| \| 0 \| \| 0 \| \| 0 \| \| 0 \| \| 0 \| \| 0 \| \| 0 \| \| 0 \| \| 0 \| \| 0 \| \| 0 \| \| 0 \| \| 0 \| \| 0 \| \| 0 \| \| 0 \| \| 0 \| \| 0 \| \| 0 \| \| 0 \| \| 0 \| \| 0 \| \| 0 \| \| 8.55 \| \| 2.62 \| \| 30.82 \| \| 37.34 \| \| 18.09 \| \| 7.85 \| \| 12.98 \| \| 27.82 \| \| 68.27 \| \| 53.29 \| \| 77.96 \| \| 0 \| \| 6.45 \| \| 20.23 \| \| 16.49 \| \| 3.59 \| \| 14.64 \| \| 9.13 \| \| 36.07 \| \| 22.89 \| \| 28.3 \| \| 0 \| \| 0 \| \| 0 \| \| 0 \| \| 0 \| \| 0 \| \| 23.21 \| \| 25.36 \| \| 19.74 \| \| 86.31 \| \| 39.23 \| \| 29.45 \| \| 8.36 \| \| 26.74 \| \| 11.57 \| \| 47.2 \| \| 18.18 \| \| 35.39 \| \| 17.9 \| \| 29.76 \| \| 20.71 \| \| 8.34 \| \| 17.77 \| \| 17.61 \| \| 19.49 \| \| 20.03 \| \| 12.48 \| \| 37.5 \| \| 0 \| \| 15.28 \| \| 24.37 \| \| 21.17 \| \| 12.65 \| \| 15 \| \| 62.78 \| \| 50.1 \| \| 38.26 \| \| 35.38 \| \| 43.95 \| \| 68.32 \| \| 73.98 \| \| 52.4 \| \| 50.26 \| \| 148.95 \| \| 62.03 \| \| 134.71 \| \| 419.75 \| \| 52.34 \| \| 28.26 \| \| 29.99 \| \| 31.93 \| \| 36.6 \| \| 19.45 \| \| 49.56 \| \| 47.06 \| \| 33.5 \| \| 84.28 \| \| 21.05 \| \| 14.68 \| \| 25.93 \| \| 76 \| \| 28.94 \| \| 59.76 \| \| 64.33 \| \| 106.41 \| \| 61.42 \| \| 28.65 \| \| 20.98 \| \| 33.3 \| \| 76.17 \| \| 55.06 \| \| 39.43 \| \| 106.31 \| \| 46.26 \| \| 8.63 \| \| 160.14 \| \| 201.35 \| \| 36.73 \| \| 42.72 \| \| 34.52 \| \| 27.49 \| \| 40.28 \| \| 34.25 \| \| 29.74 \| \| 77.16 \| \| 37.34 \| \| 39.22 \| \| 0 \| \| 27.4 \| \| 157.84 \| \| 24.02 \| \| 61.65 \| \| 46.44 \| \| 87.35 \| \| 21.32 \| \| 28.94 \| \| 28.24 \| \| 14.36 \| \| 64.92 \| \| 2.57 \| \| 20.35 \| \| 21.06 \| \| 23.09 \| \| 15.66 \| \| 62.65 \| \| 485.72 \| \| 250.69 \| \| 144.8 \| \| 0 \| \| 30.82 \| \| 26.91 \| \| 13.08 \| \| 32.71 \| \| 18.21 \| \| 19.34 \| \| 17.99 \| \| 13.49 \| \| 18.34 \| \| 28.64 \| \| 31.58 \| \| 44.68 \| \| 47.49 \| \| 57.31 \| \| 53.92 \| \| 59.05 \| \| 217.72 \| \| 85.4 \| \| 43.35 \| \| 89.97 \| \| 107.83 \| \| 12.33 \| \| 22.92 \| \| 21.55 \| \| 58.5 \| \| 33.99 \| \| 16.49 \| \| 0 \| \| 0 \| \| 46.98 \| \| 15.59 \| \| 30.3 \| \| 28.22 \| \| 36.38 \| \| 63.12 \| \| 38.57 \| \| 33.53 \| \| 104.02 \| \| 60.59 \| \| 77.5 \| \| 34.44 \| \| 54.94 \| \| 148.48 \| \| 22.44 \| \| 11.93 \| \| 14.89 \| \| 15.41 \| \| 20.5 \| \| 16.47 \| \| 20.78 \| \| 9.05 \| \| 83.56 \| \| 17.94 \| \| 21.46 \| \| 33.09 \| \| 7.99 \| \| 0 \| \| 0 \| \| 0 \| \| 0 \| \| 82.07 \| \| 12.43 \| \| 28.84 \| \| 17.66 \| \| 20.75 \| \| 70.14 \| \| 72.21 \| \| 210.73 \| \| 38.82 \| \| 69.19 \| \| 79.05 \| \| 94.43 \| \| 90.85 \| \| 279.04 \| \| 45.4 \| \| 85.85 \| \| 21.8 \| \| 16.92 \| \| 29.61 \| \| 32.62 \| \| 28.77 \| \| 90.47 \| \| 23.46 \| \| 57.03 \| \| 99.67 \| \| 98.1 \| \| 31.38 \| \| 21.88 \| \| 26.35 \| \| 16.08 \| \| 22.17 \| \| 20.25 \| \| 9.22 \| \| 17.61 \| \| 15.72 \| \| 8.15 \| \| 18.04 \| \| 1.21 \| \| 4.63 \| \| 3.77 \| \| 31.13 \| \| 18.57 \| \| 22.39 \| \| 22.42 \| \| 23.52 \| \| 19.6 \| \| 25.33 \| \| 32.88 \| \| 9.38 \| \| 19.61 \| \| 18.81 \| \| 18.47 \| \| 63.64 \| \| 31.33 \| \| 15.27 \| \| 28.08 \| \| 22.42 \| \| 25.06 \| \| 26.94 \| \| 32.39 \| \| 23.74 \| \| 35.35 \| \| 33.36 \| \| 42.02 \| \| 70.3 \| \| 124.29 \| \| 73.79 \| \| 0 \| \| 0 \| \| 0 \| \| 0 \| \| 0 \| \| 0 \| \| 0 \| \| 0 \| \| 0 \| \| 2.72 \| \| 88.6 \| \| 68.65 \| \| 172.55 \| \| 133.63 \| \| 118.02 \| \| 0 \| \| 0 \| \| 19.91 \| \| 25.16 \| \| 0 \| \| 0 \| \| 0 \| \| 0 \| \| 0 \| \| 12.41 \| \| 0 \| \| 71.55 \| \| 67.93 \| \| 21.39 \| \| 23.82 \| \| 32.69 \| \| 141.36 \| \| 96.82 \| \| 124.94 \| \| 36 \| \| 21.03 \| \| 44.09 \| \| 147.39 \| \| 67.15 \| \| 66.79 \| \| 51.52 \| \| 20.05 \| \| 17.39 \| \| 19.01 \| \| 29.85 \| \| 7.09 \| \| 18.11 \| \| 30.66 \| \| 30.98 \| \| 42.33 \| \| 24.9 \| \| 95.63 \| \| 27.12 \| \| 64.09 \| \| 68.69 \| \| 50.09 \| \| 113.39 \| \| 1,054.28 \| \| 623.14 \| \| 110.35 \| \| 0 \| \| 46.32 \| \| 35.71 \| \| 140.57 \| \| 86.08 \| \| 58.82 \| \| 182.57 \| \| 305.57 \| \| 341.48 \| \| 114.22 \| \| 17.37 \| \| 49.68 \| \| 164.77 \| \| 150.8 \| \| 0 \| \| 0 \| \| 0 \| \| 83.34 \| \| 294.65 \| \| 77.71 \| \| 185.54 \| \| 36.64 \| \| 43.6 \| \| 46.43 \| \| 74.17 \| \| 90.68 \| \| 23.19 \| \| 22.12 \| \| 41.85 \| \| 35.51 \| \| 12.51 \| \| 24.25 \| \| 8.53 \| \| 22.45 \| \| 33.82 \| \| 24.13 \| \| 26.85 \| \| 38.87 \| \| 23.71 \| \| 26.15 \| \| 27.12 \| \| 23.54 \| \| 550.16 \| \| 20.18 \| \| 19.69 \| \| 25.06 \| \| 19.41 \| \| 13.02 \| \| 21.96 \| \| 16.66 \| \| 6.66 \| \| 29.54 \| \| 15.84 \| \| 40.99 \| \| 26.79 \| \| 123.05 \| \| 45.84 \| \| 108.2 \| \| 40.54 \| \| 35.68 \| \| 126.18 \| \| 0 \| \| 26.59 \| \| 26.66 \| \| 57.13 \| \| 179.53 \| \| 604.54 \| \| 154.22 \| \| 55.67 \| \| 85.66 \| \| 55.12 \| \| 43.48 \| \| 11.43 \| \| 46.78 \| \| 47.3 \| \| 62.05 \| \| 20.07 \| \| 15.51 \| \| 32.16 \| \| 95.64 \| \| 34.01 \| \| 31.5 \| \| 29.36 \| \| 44.5 \| \| 20.45 \| \| 40.73 \| \| 35.48 \| \| 36.2 \| \| 26.87 \| \| 117.46 \| \| 52.02 \| \| 167.26 \| \| 80.09 \| \| 52.61 \| \| 34.08 \| \| 13.05 \| \| 21.95 \| \| 42.79 \| \| 40.64 \| \| 158.31 \| \| 368.18 \| \| 2,087.41 \| \| 9,365.40 \| \| 1,589.37 \| \| 1,089.79 \| \| 422.19 \| \| 164.7 \| \| 50.53 \| \| 35.1 \| \| 0 \| \| 0 \| \| 0 \| \| 0 \| \| 0 \| \| 0 \| \| 455.93 \| \| 65.32 \| \| 131.06 \| \| 0 \| \| 37.09 \| \| 33.61 \| \| 15.04 \| \| 68.97 \| \| 0 \| \| 8.55 \| \| 144.58 \| \| 331.16 \| \| 143.84 \| \| 23.75 \| \| 56.13 \| \| 103.21 \| \| 129.2 \| \| 111.27 \| \| 123.29 \| \| 39.56 \| \| 128.58 \| \| 148.55 \| \| 139.25 \| \| 202.04 \| \| 889.82 \| \| 67.68 \| \| 34.14 \| \| 115.69 \| \| 141.37 \| \| 70.45 \| \| 29.8  32.75 \| \| 363.13 \| \| 75.05 \| \| 70.04 \| \| 136.81 \| \| 360.84 \| \| 345.95 \| \| 90.71 \| \| 154.21 \| \| 304.3 \| \| 237.88 \| \| 35.92 \| \| 0 \| \| 352.64 \| \| 662.07 \| \| 430.63 \| \| 894.19 \| \| 1,434.62 \| \| 295.22 \| \| 478.3 \| \| 625.62 \| \| 687.13 \| \| 586.8 \| \| 517.18 \| \| 231.4 \| \| 90.32 \| \| 83.66 \| \| 66.53 \| \| 78.6 \| \| 22.69 \| \| 97.41 \| \| 0 \| \| 23.05 \| \| 4.65 \| \| 34.17 \| \| 51.01 \| \| 54.37 \| \| 71.68 \| \| 37.47 \| \| 633.29 \| \| 128.89 \| \| 112.96 \| \| 42.52 \| \| 89.3 \| \| 161.32 \| \| 0 \| \| 58.16 \| \| 0 \| \| 0 \| \| 0 \| \| 0 \| \| 0 \| \| 0 \| \| 0 \| \| 0 \| \| 0 \| \| 0 \| \| 0 \| \| 0 \| \| 0 \| \| 0 \| \| 0 \| \| 0 \| \| 0 \| \| 0 \| \| 0 \| \| 0 \| \| 0 \| \| 0 \| \| 0 \| \| 0 \| \| 0 \| \| 0 \| \| 0 \| \| 0 \| \| 0 \| \| 0 \| \| 0 \| \| 0 \| \| 0 \| \| 0 \| \| 0 \| \| 0 \| \| 0 \| \| 0 \| \| 0 \| \| 0 \| \| 0 \| \| 0 \| \| 0 \| \| 0 \| \| 0 \| \| 0 \| \| 0 \| \| 0 \| \| 0 \| \| 0 \| \| 0 \| \| 0 \| \| 0 \| \| 0 \| \| 0 \| \| 0 \| \| 83.36 \| \| 236.91 \| \| 71.08 \| \| 64.28 \| \| 348.81 \| \| 116.16 \| \| 152.34 \| \| 0 \| \| 104.35 \| \| 140.7 \| \| 172 \| \| 211.76 \| \| 79.07 \| \| 214.26 \| \| 76.46 \| \| 0 \| \| 66.47 \| \| 74.19 \| \| 97.5 \| \| 149.2 \| \| 100.37 \| \| 264.33 \| \| 0 \| \| 0 \| \| 66.15 \| \| 85.12 \| \| 230.91 \| \| 0 \| \| 0 \| \| 0 \| \| 15.41 \| \| 52.02 \| \| 44.71 \| \| 118.16 \| \| 48.29 \| \| 88.98 \| \| 87.57 \| \| 124.35 \| \| 84.48 \| \| 130.91 \| \| 49.88 \| \| 0 \| \| 0 \| \| 0 \| \| 0 \| \| 36.27 \| \| 652.85 \| \| 283.75 \| \| 191.28 \| \| 84.26 \| \| 101.41 \| \| 133.34 \| \| 70.69 \| \| 354.41 \| \| 196.73 \| \| 0 \| \| 0 \| \| 1,736.81 \| \| 744.57 \| \| 1,685.63 \| \| 120.57 \| \| 232.41 \| \| 565.04 \| \| 130.69 \| \| 117.42 \| \| 76.99 \| \| 181.32 \| \| 104.87 \| \| 223.67 \| \| 108.42 \| \| 134.08 \| \| 152.91 \| \| 91.13 \| \| 136.99 \| \| 109.91 \| \| 1,526.20 \| \| 102.66 \| \| 302.19 \| \| 309.29 \| \| 24.84 \| \| 80.36 \| \| 603.28 \| \| 390.96 \| \| 171.05 \| \| 93.75 \| \| 37.67 \| \| 244.96 \| \| 89.81 \| \| 526.33 \| \| 889.88 \| \| 118.89 \| \| 99.29 \| \| 71.92 \| \| 128.78 \| \| 103.17 \| \| 97.47 \| \| 102.75 \| \| 128.13 \| \| 241.15 \| \| 303.91 \| \| 274.28 \| \| 91.13 \| \| 1,242.22 \| \| 1,867.26 \| \| 1,645.05 \| \| 0 \| \| 13.89 \| \| 23.03 \| \| 34.78 \| \| 223.84 \| \| 87.03 \| \| 133.19 \| \| 158.34 \| \| 255.03 \| \| 301.08 \| \| 1,501.92 \| \| 99.65 \| \| 104.18 \| \| 230.9 \| \| 57.82 \| \| 644.12 \| \| 1,313.97 \| \| 40.34 \| \| 196.95 \| \| 97.34 \| \| 107.01 \| \| 68.23 \| \| 470.28 \| \| 28.45 \| \| 28.58 \| \| 37.34 \| \| 395.97 \| \| 124.07 \| \| 256.1 \| \| 0 \| \| 127.94 \| \| 324.89 \| \| 223.4 \| \| 87.33 \| \| 225.35 \| \| 189.67 \| \| 131.99 \| \| 5,879.47 \| \| 94.72 \| \| 55.39 \| \| 101.73 \| \| 466.67 \| \| 299.28 \| \| 232.4 \| \| 53.43 \| \| 90.29 \| \| 446.8 \| \| 53.5 \| \| 79.86 \| \| 60.54 \| \| 42.78 \| \| 63.72 \| \| 81.67 \| \| 170.36 \| \| 135.05 \| \| 93.81 \| \| 235.49 \| \| 140.03 \| \| 152.85 \| \| 327.2 \| \| 46.88 \| \| 27.25 \| \| 38.48 \| \| 223.63 \| \| 118.25 \| \| 131.34 \| \| 134.45 \| \| 73.87 \| \| 118.5 \| \| 21.53 \| \| 95.27 \| \| 67.23 \| \| 290.88 \| \| 116.72 \| \| 92.66 \| \| 132.93 \| \| 35.38 \| \| 271.56 \| \| 194.9 \| \| 5,325.41 \| \| 4,394.31 \| \| 139.1 \| \| 0 \| \| 0 \| \| 82.81 \| \| 35.38 \| \| 23.91 \| \| 23.14 \| \| 29.84 \| \| 88.11 \| \| 48.51 \| \| 39.23 \| \| 93.61 \| \| 67.08 \| \| 42.94 \| \| 78.49 \| \| 86.27 \| \| 186.02 \| \| 110.86 \| \| 18.84 \| \| 594.34 \| \| 206.83 \| \| 136.36 \| \| 57.2 \| \| 1,259.26 \| \| 66.72 \| \| 309.75 \| \| 139.41 \| \| 124.87 \| \| 70.15 \| \| 135.68 \| \| 154.12 \| \| 983.75 \| \| 114.77 \| \| 80.17 \| \| 168.49 \| \| 143.44 \| \| 262.23 \| \| 362.63 \| \| 51.83 \| \| 164.73 \| \| 358.68 \| \| 150.74 \| \| 98.64 \| \| 255.13 \| \| 363.5 \| \| 150.1 \| \| 126.28 \| \| 71.49 \| \| 84.97 \| \| 20.88 \| \| 69.28 \| \| 122.51 \| \| 148.98 \| \| 26.59 \| \| 126.15 \| \| 0 \| \| 101.45 \| \| 57.08 \| \| 50.97 \| \| 43.88 \| \| 33.17 \| \| 0 \| \| 0 \| \| 39.75 \| \| 86.07 \| \| 131.56 \| \| 403.21 \| \| 342.74 \| \| 109.4 \| \| 131.41 \| \| 22.06 \| \| 90.85 \| \| 33.19 \| \| 213.02 \| \| 44.43 \| \| 23.38 \| \| 78.93 \| \| 159.06 \| \| 116.48 \| \| 21.76 \| \| 35.53 \| \| 23.48 \| \| 0 \| \| 336.73 \| \| 747.17 \| \| 214.29 \| \| 199.43 \| \| 2,437.13 \| \| 3,217.30 \| \| 1,678.45 \| \| 367.94 \| \| 28.64 \| \| 14.01 \| \| 63.78 \| \| 49.88 \| \| 67.7 \| \| 0 \| \| 1,649.14 \| \| 759.61 \| \| 68.71 \| \| 251.41 \| \| 834.69 \| \| 674.94 \| \| 525.1 \| \| 0 \| \| 0 \| \| 406.09 \| \| 421.85 \| \| 503.45 \| \| 296.15 \| \| 370.45 \| \| 406.72 \| \| 532.36 \| \| 719.08 \| \| 698.12 \| \| 802.82 \| \| 656.06 \| \| 677.63 \| \| 870.4 \| \| 635.65 \| \| 0 \| \| 318.37 \| \| 493.18 \| \| 558.23 \| \| 81.41 \| \| 161.08 \| \| 324.19 \| \| 163.8 \| \| 269.67 \| \| 506.51 \| \| 90.87 \| \| 5.97 \| \| 108.72 \| \| 48.73 \| \| 0 \| \| 0 \| \| 138.5 \| \| 80.12 \| \| 136.54 \| \| 160.75 \| \| 526.61 \| \| 147.17 \| \| 212.75 \| \| 243.01 \| \| 110.79 \| \| 159.27 \| \| 206.2 \| \| 254.84 \| \| 209.75 \| \| 110.16 \| \| 22.54 \| \| 127.91 \| \| 80.36 \| \| 268.45 \| \| 72.66 \| \| 423.46 \| \| 296.68 \| \| 756.74 \| \| 892.41 \| \| 814.75 \| \| 0 \| \| 333.45 \| \| 71.74 \| \| 167.96 \| \| 169.87 \| \| 56.15 \| \| 66.27 \| \| 94.72 \| \| 30.45 \| \| 63.88 \| \| 443.4 \| \| 68.36 \| \| 166.57 \| \| 105.56 \| \| 170.32 \| \| 69.78 \| \| 50.93 \| \| 90.7 \| \| 168.32 \| \| 644.71 \| \| 111.94 \| \| 160.41 \| \| 235.08 \| \| 61.5 \| \| 208.81 \| \| 125.72 \| \| 233.56 \| \| 327.27 \| \| 241.89 \| \| 37.46 \| \| 0 \| \| 49.48 \| \| 308.69 \| \| 77.4 \| \| 198.48 \| \| 274.41 \| \| 308.73 \| \| 61.95 \| \| 262.97 \| \| 120.84 \| \| 77.34 \| \| 54.71 \| \| 89.31 \| \| 90.35 \| \| 27.76 \| \| 148.83 \| \| 39.37 \| \| 53.88 \| \| 204.94 \| \| 212.25 \| \| 97.01 \| \| 57.18 \| \| 396.46 \| \| 33.46 \| \| 80.18 \| \| 71.82 \| \| 19.57 \| \| 131.88 \| \| 118.8 \| \| 595.56 \| \| 43.18 \| \| 82.2 \| \| 95 \| \| 108.62 \| \| 45.02 \| \| 56.86 \| \| 101.39 \| \| 85.89 \| \| 222.06 \| \| 53.9 \| \| 112.71 \| \| 44.35 \| \| 29.88 \| \| 179.89 \| \| 168.64 \| \| 516.14 \| \| 10,514.68 \| \| 199.79 \| \| 58.49 \| \| 32.21 \| \| 36.11 \| \| 29.17 \| \| 67.99 \| \| 228.98 \| \| 75.2 \| \| 784.44 \| \| 144.33 \| \| 279.91 \| \| 138.34 \| \| 153.06 \| \| 115.68 \| \| 164.3 \| \| 314.07 \| \| 0 \| \| 0 \| \| 6.16 \| \| 77.57 \| \| 329.47 \| \| 214 \| \| 298.6 \| \| 187.97 \| \| 305.89 \| \| 1,006.11 \| \| 343.88 \| \| 540.83 \| \| 169.17 \| \| 90.76 \| \| 2,608.20 \| \| 398.9 \| \| 227.92 \| \| 283.9 \| \| 90.42 \| \| 99.05 \| \| 42.68 \| \| 58.92 \| \| 123.29 \| \| 48.51 \| \| 40.28 \| \| 31.83 \| \| 0 \| \| 217.79 \| \| 99.77 \| \| 40.44 \| \| 58.48 \| \| 664.97 \| \| 53.1 \| \| 101.93 \| \| 172.41  23.53 \| \| 40.6 \| \| 49.92 \| \| 88.26 \| \| 27.25 \| \| 58.97 \| \| 28.42 \| \| 28.37 \| \| 48.14 \| \| 736.29 \| \| 185.85 \| \| 1.99 \| \| 519.29 \| \| 491.87 \| \| 76.87 \| \| 302.27 \| \| 296.65 \| \| 395.92 \| \| 367.32 \| \| 36.42 \| \| 151.63 \| \| 125.57 \| \| 244.08 \| \| 0 \| \| 0 \| \| 312.4 \| \| 1,244.45 \| \| 326.86 \| \| 91.54 \| \| 0 \| \| 44.52 \| \| 26.35 \| \| 56.04 \| \| 251.52 \| \| 90.87 \| \| 16.32 \| \| 113.08 \| \| 168.16 \| \| 23.12 \| \| 177.26 \| \| 47.16 \| \| 59.8 \| \| 94.93 \| \| 64.09 \| \| 71.65 \| \| 351.81 \| \| 30.42 \| \| 337.79 \| \| 96.18 \| \| 73.08 \| \| 115.81 \| \| 191.75 \| \| 42.78 \| \| 202.11 \| \| 141.24 \| \| 60.9 \| \| 61.14 \| \| 118.94 \| \| 98.14 \| \| 201.91 \| \| 341.78 \| \| 3,009.59 \| \| 265.08 \| \| 62.86 \| \| 129.71 \| \| 22.92 \| \| 71.66 \| \| 49.74 \| \| 533.84 \| \| 42.4 \| \| 43.44 \| \| 3,087.90 \| \| 16.44 \| \| 2,025.96 \| \| 494.18 \| \| 188.72 \| \| 494.72 \| \| 42.89 \| \| 33.91 \| \| 157.2 \| \| 127.44 \| \| 277.41 \| \| 90.33 \| \| 139.33 \| \| 287.95 \| \| 390.81 \| \| 359.31 \| \| 467.58 \| \| 329.48 \| \| 454.46 \| \| 1,302.88 \| \| 177.14 \| \| 86.1 \| \| 147.13 \| \| 125.28 \| \| 216.12 \| \| 198.94 \| \| 132.08 \| \| 96.9 \| \| 54.59 \| \| 267.68 \| \| 252.1 \| \| 139.14 \| \| 1,563.08 \| \| 113.05 \| \| 88.99 \| \| 36.8 \| \| 22.35 \| \| 43.98 \| \| 18.49 \| \| 53.34 \| \| 71.24 \| \| 39.84 \| \| 118.93 \| \| 0 \| \| 32.9 \| \| 103.86 \| \| 73.77 \| \| 887.9 \| \| 5,720.74 \| \| 696.34 \| \| 280.22 \| \| 248.31 \| \| 424.42 \| \| 170.39 \| \| 213.19 \| \| 72.82 \| \| 97.14 \| \| 118.33 \| \| 416.88 \| \| 80.05 \| \| 213.29 \| \| 309.93 \| \| 70.1 \| \| 211.45 \| \| 84.02 \| \| 68.14 \| \| 52.05 \| \| 247.75 \| \| 91.85 \| \| 217.4 \| \| 52.31 \| \| 84.68 \| \| 52.47 \| \| 68.34 \| \| 807.28 \| \| 81.01 \| \| 28.61 \| \| 46.55 \| \| 63.57 \| \| 109.36 \| \| 80.41 \| \| 94.75 \| \| 102.83 \| \| 112.29 \| \| 95.66 \| \| 25.33 \| \| 42.41 \| \| 78.68 \| \| 161.03 \| \| 368.22 \| \| 54.65 \| \| 235.5 \| \| 2,124.73 \| \| 2,278.11 \| \| 98.32 \| \| 231.51 \| \| 265.15 \| \| 37.17 \| \| 1,103.53 \| \| 522.54 \| \| 305.67 \| \| 171.65 \| \| 44.28 \| \| 14.23 \| \| 114.37 \| \| 469.72 \| \| 301.39 \| \| 159.16 \| \| 86.26 \| \| 146.94 \| \| 253.87 \| \| 45.8 \| \| 0 \| \| 1,135.44 \| \| 49.69 \| \| 235.42 \| \| 165.06 \| \| 80.26 \| \| 4.6 \| \| 59.45 \| \| 279.38 \| \| 99.05 \| \| 414.64 \| \| 144.36 \| \| 209.34 \| \| 141.78 \| \| 29.72 \| \| 34.55 \| \| 52.46 \| \| 40.44 \| \| 81.34 \| \| 70.29 \| \| 121.37 \| \| 334.75 \| \| 383.44 \| \| 1,823.29 \| \| 172.97 \| \| 234.71 \| \| 419.45 \| \| 129.47 \| \| 75.56 \| \| 2,025.72 \| \| 18,487.78 \| \| 232.59 \| \| 319.55 \| \| 118.7 \| \| 135.96 \| \| 179.89 \| \| 56.34 \| \| 93.37 \| \| 549.46 \| \| 137.03 \| \| 49.15 \| \| 97.43 \| \| 55.94 \| \| 73.15 \| \| 62.44 \| \| 171.6 \| \| 870.39 \| \| 631.71 \| \| 526.4 \| \| 440.04 \| \| 947.42 \| \| 162.82 \| \| 209.89 \| \| 38.36 \| \| 2.29 \| \| 0 \| \| 0.32 \| \| 2.71 \| \| 0 \| \| 17.66 \| \| 1,545.82 \| \| 1,850.95 \| \| 289.13 \| \| 199.83 \| \| 912.76 \| \| 149.8 \| \| 609.08 \| \| 127.18 \| \| 247.92 \| \| 170.44 \| \| 123.29 \| \| 211.26 \| \| 111.85 \| \| 125.4 \| \| 0 \| \| 313.72 \| \| 0 \| \| 0 \| \| 202.77 \| \| 144.69 \| \| 73.03 \| \| 102.85 \| \| 72.95 \| \| 177.8 \| \| 103.57 \| \| 517.34 \| \| 83.8 \| \| 208.18 \| \| 278.26 \| \| 92.16 \| \| 87.8 \| \| 104.03 \| \| 274.56 \| \| 229.1 \| \| 233.57 \| \| 73.21 \| \| 95.57 \| \| 318.85 \| \| 722.51 \| \| 55.21 \| \| 368.98 \| \| 63.13 \| \| 81.72 \| \| 150.39 \| \| 219.55 \| \| 48.41 \| \| 103.77 \| \| 142.18 \| \| 86.24 \| \| 169.9 \| \| 1,432.32 \| \| 233.75 \| \| 97.69 \| \| 235.58 \| \| 97.25 \| \| 32.36 \| \| 26.05 \| \| 34.3 \| \| 22.13 \| \| 41.52 \| \| 361.28 \| \| 39.47 \| \| 55.87 \| \| 16.98 \| \| 16.54 \| \| 929.88 \| \| 154.61 \| \| 14.9 \| \| 4,321.07 \| \| 194.39 \| \| 282.77 \| \| 39.63 \| \| 77.79 \| \| 43.5 \| \| 0 \| \| 46.78 \| \| 63.73 \| \| 41.1 \| \| 10.27 \| \| 22.69 \| \| 55.94 \| \| 22.29 \| \| 89.41 \| \| 65.16 \| \| 122.25 \| \| 44.44 \| \| 97.57 \| \| 100.95 \| \| 144.88 \| \| 309.05 \| \| 617.62 \| \| 747.66 \| \| 40.39 \| \| 85.14 \| \| 153.92 \| \| 71.92 \| \| 142.52 \| \| 351.2 \| \| 72.58 \| \| 198.37 \| \| 8.61 \| \| 800.29 \| \| 52.84 \| \| 235.52 \| \| 44.36 \| \| 88.07 \| \| 33.63 \| \| 33.48 \| \| 63.79 \| \| 21.07 \| \| 28.11 \| \| 16.97 \| \| 22.95 \| \| 26.55 \| \| 79.05 \| \| 189.48 \| \| 241.95 \| \| 101.75 \| \| 56 \| \| 95.32 \| \| 657.97 \| \| 172.34 \| \| 164.71 \| \| 109.13 \| \| 95 \| \| 77.13 \| \| 275.25 \| \| 648.12 \| \| 38.48 \| \| 19.81 \| \| 24.42 \| \| 77.36 \| \| 24 \| \| 11.78 \| \| 36.03 \| \| 96.59 \| \| 47.26 \| \| 189.61 \| \| 53.61 \| \| 77.57 \| \| 41.55 \| \| 92.74 \| \| 39.03 \| \| 21.26 \| \| 73.52 \| \| 53.08 \| \| 83.48 \| \| 103.24 \| \| 93.39 \| \| 39.96 \| \| 45.28 \| \| 246.59 \| \| 330.17 \| \| 234.09 \| \| 491.41 \| \| 213.36 \| \| 36.14 \| \| 52.84 \| \| 24.12 \| \| 42.11 \| \| 0 \| \| 46.14 \| \| 0 \| \| 62.53 \| \| 183.08 \| \| 128.44 \| \| 210.5 \| \| 0 \| \| 0 \| \| 0 \| \| 29.72 \| \| 86 \| \| 16.32 \| \| 0 \| \| 35.52 \| \| 61.65 \| \| 78.87 \| \| 101.01 \| \| 58.53 \| \| 64.23 \| \| 50.77 \| \| 76.87 \| \| 123.14 \| \| 58.35 \| \| 132.35 \| \| 89.21 \| \| 96.66 \| \| 46.73 \| \| 24.19 \| \| 175.65 \| \| 329.63 \| \| 196.13 \| \| 183.98 \| \| 102.04 \| \| 279.66 \| \| 51.47 \| \| 88.25 \| \| 386.19 \| \| 410.5 \| \| 307.98 \| \| 567.42 \| \| 49.25 \| \| 577.2 \| \| 289.44 \| \| 620.99 \| \| 22.63 \| \| 45.86 \| \| 59.69 \| \| 31.97 \| \| 68.08 \| \| 414.4 \| \| 252.1 \| \| 26.08 \| \| 168.08 \| \| 330.98 \| \| 708.85 \| \| 551.91 \| \| 116.76 \| \| 12.75 \| \| 61.65 \| \| 38.48 \| \| 102.44 \| \| 159.42 \| \| 212.25 \| \| 168.69 \| \| 63.32 \| \| 522.52 \| \| 479.11 \| \| 97.73 \| \| 380.3 \| \| 453.67 \| \| 249.3 \| \| 67.73 \| \| 229.08 \| \| 85.48 \| \| 325.96 \| \| 112 \| \| 173.38 \| \| 62.18 \| \| 92.84 \| \| 117.93 \| \| 354.57 \| \| 141.56 \| \| 64.87 \| \| 181.74 \| \| 60.61 \| \| 190.43 \| \| 319.28 \| \| 385.4 \| \| 687.54 \| \| 342.93 \| \| 24.89 \| \| 28.26 \| \| 9.11 \| \| 13.36 \| \| 25.41 \| \| 146.64 \| \| 98.42 \| \| 96.97 \| \| 214.14 \| \| 69.69 \| \| 602.48 \| \| 1,630.44 \| \| 1,099.81 \| \| 1,804.74 \| \| 493.88 \| \| 1,214.89 \| \| 2,075.46 \| \| 878.3 \| \| 251.03 \| \| 141.72 \| \| 550.64 \| \| 772.91 \| \| 31.65 \| \| 2,153.01 \| \| 106.84 \| \| 90.01 \| \| 431.76 \| \| 175.89 \| \| 337.99 \| \| 183.73 \| \| 107.63 \| \| 96.01 \| \| 208.48  66.83 \| \| 92.47 \| \| 119.84 \| \| 79.04 \| \| 72.64 \| \| 50.49 \| \| 172.54 \| \| 13.3 \| \| 32.14 \| \| 45.8 \| \| 44.26 \| \| 36.99 \| \| 138.61 \| \| 171.59 \| \| 167.02 \| \| 163.37 \| \| 94.72 \| \| 18.3 \| \| 53.46 \| \| 35.23 \| \| 36.17 \| \| 48.93 \| \| 57.04 \| \| 132.36 \| \| 260.68 \| \| 46.12 \| \| 66.06 \| \| 66.83 \| \| 57.74 \| \| 13.7 \| \| 0 \| \| 18.71 \| \| 0 \| \| 771.09 \| \| 43.61 \| \| 138.81 \| \| 193.24 \| \| 53.23 \| \| 50.71 \| \| 129.23 \| \| 126.46 \| \| 129.56 \| \| 0 \| \| 48.54 \| \| 74.42 \| \| 141.31 \| \| 501.11 \| \| 471.65 \| \| 41.64 \| \| 515.81 \| \| 251.95 \| \| 243.42 \| \| 201.93 \| \| 92.97 \| \| 47.17 \| \| 95.57 \| \| 104.73 \| \| 55.57 \| \| 225.43 \| \| 111.37 \| \| 109.38 \| \| 160.1 \| \| 89.84 \| \| 273.26 \| \| 201.07 \| \| 110.88 \| \| 53.54 \| \| 81.93 \| \| 0 \| \| 17.42 \| \| 73.26 \| \| 189.08 \| \| 47.31 \| \| 58.3 \| \| 49.92 \| \| 54.39 \| \| 58.66 \| \| 56.95 \| \| 24.31 \| \| 62.28 \| \| 165.6 \| \| 8,033.22 \| \| 2,961.65 \| \| 53.49 \| \| 78.56 \| \| 139.97 \| \| 68.86 \| \| 23.56 \| \| 26.96 \| \| 46.92 \| \| 47.95 \| \| 34.73 \| \| 36.95 \| \| 20.11 \| \| 29.85 \| \| 40.24 \| \| 32.06 \| \| 39.14 \| \| 30.94 \| \| 62.81 \| \| 207.36 \| \| 39.65 \| \| 0 \| \| 40.65 \| \| 26.89 \| \| 79.98 \| \| 469.29 \| \| 536.71 \| \| 214.04 \| \| 148.68 \| \| 112.85 \| \| 78.5 \| \| 154.12 \| \| 66.48 \| \| 56.36 \| \| 35.47 \| \| 55.82 \| \| 94.79 \| \| 113.72 \| \| 191.48 \| \| 123.01 \| \| 138.85 \| \| 194.43 \| \| 781.15 \| \| 222.76 \| \| 110.13 \| \| 131.49 \| \| 267.05 \| \| 337.08 \| \| 379.97 \| \| 377.39 \| \| 60.36 \| \| 360.04 \| \| 55.77 \| \| 52.4 \| \| 19.51 \| \| 22.23 \| \| 23.81 \| \| 779.37 \| \| 317.97 \| \| 124.55 \| \| 156.72 \| \| 182.98 \| \| 113.19 \| \| 54.21 \| \| 49.15 \| \| 129.58 \| \| 70.72 \| \| 120.36 \| \| 87.33 \| \| 924.05 \| \| 118.09 \| \| 220.76 \| \| 336 \| \| 379.3 \| \| 175 \| \| 125.78 \| \| 106.54 \| \| 207.19 \| \| 102.88 \| \| 813.59 \| \| 73.98 \| \| 181.07 \| \| 0 \| \| 0 \| \| 1,896.48 \| \| 1,729.58 \| \| 278.7 \| \| 58.77 \| \| 765.82 \| \| 59.5 \| \| 84.95 \| \| 115.04 \| \| 152.61 \| \| 107.52 \| \| 100.65 \| \| 141.79 \| \| 155.37 \| \| 94.66 \| \| 112.57 \| \| 292.07 \| \| 80.85 \| \| 383.36 \| \| 89.81 \| \| 70.79 \| \| 60.24 \| \| 52.74 \| \| 56.91 \| \| 37.64 \| \| 80.62 \| \| 80.41 \| \| 96.83 \| \| 46.15 \| \| 724.39 \| \| 1,729.24 \| \| 110.42 \| \| 50.89 \| \| 62.22 \| \| 190.44 \| \| 380.49 \| \| 84.03 \| \| 1,654.41 \| \| 1,424.47 \| \| 733.5 \| \| 1,317.09 \| \| 18.16 \| \| 32.39 \| \| 71.53 \| \| 47.59 \| \| 125.29 \| \| 48.53 \| \| 61.12 \| \| 85.48 \| \| 242.34 \| \| 53.8 \| \| 29.06 \| \| 258.84 \| \| 46.77 \| \| 29.23 \| \| 53.8 \| \| 43.48 \| \| 1.88 \| \| 31.11 \| \| 194.53 \| \| 239.21 \| \| 162.77 \| \| 171.49 \| \| 733.12 \| \| 260.77 \| \| 145.31 \| \| 92.89 \| \| 62.06 \| \| 0 \| \| 0 \| \| 0 \| \| 24.54 \| \| 41.96 \| \| 29.12 \| \| 197.16 \| \| 87.27 \| \| 0 \| \| 57.95 \| \| 28.67 \| \| 33.65 \| \| 0 \| \| 58.97 \| \| 117.61 \| \| 79.28 \| \| 54.15 \| \| 26.11 \| \| 64.26 \| \| 56.51 \| \| 22.81 \| \| 23.33 \| \| 24.43 \| \| 50.33 \| \| 29.08 \| \| 88.03 \| \| 25.19 \| \| 18.49 \| \| 26.69 \| \| 57.67 \| \| 32.32 \| \| 0 \| \| 22.34 \| \| 25.34 \| \| 54.49 \| \| 51 \| \| 67.58 \| \| 75.71 \| \| 50.73 \| \| 78.13 \| \| 88.54 \| \| 0 \| \| 107.68 \| \| 68.33 \| \| 95.48 \| \| 51.7 \| \| 22.74 \| \| 57.98 \| \| 101.07 \| \| 42.26 \| \| 31.43 \| \| 46.12 \| \| 35.35 \| \| 42.11 \| \| 84.74 \| \| 68.21 \| \| 54.43 \| \| 60.47 \| \| 134.69 \| \| 58.91 \| \| 248.44 \| \| 82.23 \| \| 87.43 \| \| 90.42 \| \| 28.18 \| \| 162.81 \| \| 60.42 \| \| 96.69 \| \| 21.98 \| \| 26.56 \| \| 33.45 \| \| 34.72 \| \| 73.13 \| \| 53.59 \| \| 52.64 \| \| 78.99 \| \| 45.58 \| \| 137.89 \| \| 128.69 \| \| 25.64 \| \| 101.57 \| \| 35 \| \| 27.24 \| \| 33.68 \| \| 44.79 \| \| 38.6 \| \| 66.6 \| \| 131.65 \| \| 83.63 \| \| 59.97 \| \| 51.28 \| \| 51.19 \| \| 82.07 \| \| 50.4 \| \| 0 \| \| 81.63 \| \| 356.71 \| \| 733.96 \| \| 67.38 \| \| 144.75 \| \| 53.29 \| \| 261.46 \| \| 93.76 \| \| 132.21 \| \| 221.93 \| \| 134.58 \| \| 245.14 \| \| 235.31 \| \| 165.22 \| \| 140.5 \| \| 247.59 \| \| 180.94 \| \| 130.44 \| \| 84.28 \| \| 11.6 \| \| 24.1 \| \| 41.8 \| \| 74.6 \| \| 86.65 \| \| 82.12 \| \| 31.49 \| \| 18.91 \| \| 48.88 \| \| 38.23 \| \| 228.34 \| \| 171.39 \| \| 99.94 \| \| 63.57 \| \| 117.78 \| \| 19.15 \| \| 104.89 \| \| 115.17 \| \| 106.53 \| \| 91.79 \| \| 0 \| \| 139.25 \| \| 166.97 \| \| 0 \| \| 309.72 \| \| 257.19 \| \| 131.05 \| \| 60.32 \| \| 213.11 \| \| 74.14 \| \| 38.36 \| \| 111.51 \| \| 0 \| \| 0 \| \| 0 \| \| 102.22 \| \| 0 \| \| 0 \| \| 0 \| \| 0 \| \| 0 \| \| 101.19 \| \| 68.53 \| \| 72.36 \| \| 159.19 \| \| 205.49 \| \| 439.5 \| \| 105.98 \| \| 116.22 \| \| 53.54 \| \| 81.78 \| \| 50.38 \| \| 100.23 \| \| 69.43 \| \| 87.48 \| \| 52.23 \| \| 84.42 \| \| 41.23 \| \| 42.7 \| \| 132.47 \| \| 119.27 \| \| 140.5 \| \| 97.49 \| \| 68.14 \| \| 81.53 \| \| 46.69 \| \| 64.82 \| \| 0 \| \| 0 \| \| 13.7 \| \| 35.07 \| \| 29.14 \| \| 125.79 \| \| 45.8 \| \| 55.38 \| \| 50.11 \| \| 74.94 \| \| 64.33 \| \| 57.76 \| \| 110.19 \| \| 181.14 \| \| 395.84 \| \| 2,687.66 \| \| 198.6 \| \| 0 \| \| 31.67 \| \| 216.74 \| \| 151.48 \| \| 63.34 \| \| 82.94 \| \| 1,407.75 \| \| 64.24 \| \| 92.83 \| \| 115.02 \| \| 30.59 \| \| 255.48 \| \| 68.43 \| \| 67.47 \| \| 62.91 \| \| 100.97 \| \| 72.28 \| \| 119.2 \| \| 96.49 \| \| 142.42 \| \| 198.55 \| \| 255.86 \| \| 135.45 \| \| 45.62 \| \| 126.01 \| \| 171.46 \| \| 44.6 \| \| 84.35 \| \| 119.41 \| \| 707.23 \| \| 273.25 \| \| 56.51 \| \| 62.67 \| \| 218 \| \| 7.01 \| \| 0 \| \| 0 \| \| 134.61 \| \| 210.15 \| \| 237.68 \| \| 204.82 \| \| 83.41 \| \| 106.46 \| \| 70.91 \| \| 143.52 \| \| 208.96 \| \| 292.83 \| \| 489.04 \| \| 121.37 \| \| 70.52 \| \| 113.11 \| \| 0 \| \| 5,224.83 \| \| 0 \| \| 0 \| \| 314.43 \| \| 2.89 \| \| 5,220.21 \| \| 0 \| \| 0 \| \| 310.62 \| \| 65.78 \| \| 82.2 \| \| 135.67 \| \| 118.92 \| \| 33.38 \| \| 42.46 \| \| 66.27 \| \| 105.41 \| \| 55.32 \| \| 130.41 \| \| 92.21 \| \| 119.02 \| \| 342.69 \| \| 459.92 \| \| 56.36 \| \| 77.36 \| \| 48.31 \| \| 39.71 \| \| 35.23 \| \| 44.34 \| \| 79.05 \| \| 86.82 \| \| 90.7 \| \| 355.35 \| \| 160.28 \| \| 210.66 \| \| 547.3 \| \| 121.86 \| \| 373.16 \| \| 275.72 \| \| 67.67 \| \| 214.34 \| \| 31.13 \| \| 37.06 \| \| 42.89 \| \| 49.91 \| \| 21.86 \| \| 0 \| \| 271.97 \| \| 162.9 \| \| 373.68 \| \| 167.08 \| \| 141.53 \| \| 127.27 \| \| 66.31 \| \| 55.07 \| \| 281.29 \| \| 296.66 \| \| 275.45 \| | \| 273.14 \| \| --- \| \| 401.82 \| \| 33.56 \| \| 151.51 \| \| 617.74 \| \| 145.26 \| \| 316.58 \| \| 1,392.95 \| \| 716.4 \| \| 1,616.13 \| \| 1,079.51 \| \| 895.2 \| \| 37.41 \| \| 61.09 \| \| 154.31 \| \| 66.73 \| \| 0 \| \| 0 \| \| 0 \| \| 0 \| \| 0 \| \| 0 \| \| 0 \| \| 0 \| \| 0 \| \| 0 \| \| 0 \| \| 0 \| \| 0 \| \| 0 \| \| 0 \| \| 0 \| \| 0.31 \| \| 0 \| \| 0 \| \| 0 \| \| 0 \| \| 239.26 \| \| 0 \| \| 0 \| \| 0 \| \| 0 \| \| 235.61 \| \| 378.73 \| \| 598.4 \| \| 40.15 \| \| 20.16 \| \| 23.51 \| \| 31.95 \| \| 84.78 \| \| 286.19 \| \| 0 \| \| 0 \| \| 0 \| \| 0 \| \| 0 \| \| 0 \| \| 0 \| \| 0 \| \| 0 \| \| 0 \| \| 0 \| \| 0 \| \| 0 \| \| 0 \| \| 44.03 \| \| 181.63 \| \| 241.32 \| \| 0 \| \| 990.45 \| \| 133.75 \| \| 97.11 \| \| 115.94 \| \| 128.47 \| \| 98.83 \| \| 45.04 \| \| 13.61 \| \| 16.94 \| \| 75.14 \| \| 59.53 \| \| 21.37 \| \| 103.42 \| \| 184.16 \| \| 173.94 \| \| 110.45 \| \| 148.69 \| \| 252.73 \| \| 162.01 \| \| 155.76 \| \| 248.85 \| \| 205.8 \| \| 89.83 \| \| 167.36 \| \| 212.47 \| \| 424.06 \| \| 160.45 \| \| 73.86 \| \| 20.86 \| \| 37.35 \| \| 85.88 \| \| 20.21 \| \| 32.84 \| \| 16.67 \| \| 60.75 \| \| 56.88 \| \| 31.16 \| \| 55.99 \| \| 56.17 \| \| 18.48 \| \| 69.3 \| \| 128.71 \| \| 122.3 \| \| 108.76 \| \| 44.16 \| \| 30.61 \| \| 7.78 \| \| 18.39 \| \| 33.19 \| \| 23.3 \| \| 51.6 \| \| 64.43 \| \| 40.42 \| \| 55.21 \| \| 0 \| \| 0 \| \| 103.36 \| \| 191.19 \| \| 72.74 \| \| 198.14 \| \| 278.65 \| \| 75.35 \| \| 80.49 \| \| 65.42 \| \| 217.97 \| \| 119.67 \| \| 267.31 \| \| 197.1 \| \| 40.42 \| \| 538.99 \| \| 70.78 \| \| 382.8 \| \| 40.07 \| \| 50.61 \| \| 70.57 \| \| 111.68 \| \| 167.34 \| \| 25.29 \| \| 68.82 \| \| 58.92 \| \| 274.04 \| \| 629.26 \| \| 94.58 \| \| 242.55 \| \| 296.2 \| \| 96.07 \| \| 148.47 \| \| 75.9 \| \| 135.44 \| \| 117.67 \| \| 277.62 \| \| 539.62 \| \| 291.56 \| \| 31.44 \| \| 70.34 \| \| 20.08 \| \| 62.26 \| \| 55.82 \| \| 44.1 \| \| 58.56 \| \| 0 \| \| 280.76 \| \| 689.44 \| \| 65.63 \| \| 2,744.94 \| \| 96.82 \| \| 104.29 \| \| 249.1 \| \| 49.63 \| \| 33.34 \| \| 73.1 \| \| 48.5 \| \| 258.4 \| \| 265.28 \| \| 535.95 \| \| 144.74 \| \| 52.93 \| \| 63.55 \| \| 250.32 \| \| 170.66 \| \| 187.3 \| \| 194.58 \| \| 213.9 \| \| 157.63 \| \| 399.71 \| \| 58.44 \| \| 47.91 \| \| 147.57 \| \| 74.25 \| \| 14.51 \| \| 95.56 \| \| 195.77 \| \| 178.3 \| \| 0 \| \| 0 \| \| 0 \| \| 0 \| \| 94.26 \| \| 112.6 \| \| 93.07 \| \| 139.78 \| \| 185.05 \| \| 379.47 \| \| 29.66 \| \| 26.55 \| \| 161.44 \| \| 252.39 \| \| 115.55 \| \| 394.15 \| \| 39.85 \| \| 63.59 \| \| 109.04 \| \| 29.31 \| \| 2.83 \| \| 374.57 \| \| 330.42 \| \| 455.37 \| \| 72.75 \| \| 191.3 \| \| 73.25 \| \| 30.8 \| \| 240.14 \| \| 148.08 \| \| 1,304.14 \| \| 1,088.13 \| \| 67.06 \| \| 48.31 \| \| 61.14 \| \| 74.51 \| \| 116.9 \| \| 82.98 \| \| 168.15 \| \| 102.64 \| \| 141.69 \| \| 14.97 \| \| 185.09 \| \| 454.6 \| \| 264.46 \| \| 67.37 \| \| 2,554.53 \| \| 6,773.04 \| \| 2,679.57 \| \| 1,712.68 \| \| 2,014.42 \| \| 88.82 \| \| 46.63 \| \| 11.1 \| \| 7.81 \| \| 106.53 \| \| 107.8 \| \| 267.41 \| \| 487.25 \| \| 1,169.71 \| \| 252.97 \| \| 14.78 \| \| 84.95 \| \| 419.97 \| \| 202.25 \| \| 64.06 \| \| 39.62 \| \| 0 \| \| 0 \| \| 0 \| \| 0 \| \| 0 \| \| 0 \| \| 0 \| \| 0 \| \| 0 \| \| 0 \| \| 0 \| \| 0 \| \| 0 \| \| 0 \| \| 0 \| \| 0 \| \| 0 \| \| 0 \| \| 0 \| \| 0 \| \| 0 \| \| 0 \| \| 0 \| \| 0 \| \| 0 \| \| 0 \| \| 0 \| \| 0 \| \| 0 \| \| 0 \| \| 0 \| \| 0 \| \| 0 \| \| 0 \| \| 0 \| \| 0 \| \| 0 \| \| 0 \| \| 0 \| \| 0 \| \| 0 \| \| 0 \| \| 0 \| \| 0 \| \| 0 \| \| 0 \| \| 0 \| \| 0 \| \| 503.06 \| \| 171.84 \| \| 108.8 \| \| 137.43 \| \| 61.24 \| \| 0 \| \| 696.65 \| \| 164.04 \| \| 61.18 \| \| 28.35 \| \| 292.13 \| \| 51.51 \| \| 98.09 \| \| 319.88 \| \| 35.28 \| \| 132.99 \| \| 0 \| \| 0 \| \| 0 \| \| 0 \| \| 0 \| \| 70.32 \| \| 64.9 \| \| 299.98 \| \| 42.29 \| \| 37.42 \| \| 34.68 \| \| 47.19 \| \| 16.22 \| \| 8.67 \| \| 48.44 \| \| 43.98 \| \| 0 \| \| 1.39 \| \| 27.18 \| \| 45.77 \| \| 99.3 \| \| 544.2 \| \| 339.57 \| \| 0 \| \| 0 \| \| 55.65 \| \| 195.3 \| \| 323.4 \| \| 740.45 \| \| 293.4 \| \| 15.16 \| \| 576.98 \| \| 26.39 \| \| 26.05 \| \| 42.34 \| \| 7,386.20 \| \| 154 \| \| 311.79 \| \| 67.61 \| \| 83.96 \| \| 136.21 \| \| 205.23 \| \| 19.02 \| \| 255.97 \| \| 264.83 \| \| 307.5 \| \| 15.14 \| \| 24.1 \| \| 117.27 \| \| 98.93 \| \| 129.75 \| \| 77.69 \| \| 43.14 \| \| 19.37 \| \| 31.59 \| \| 30.64 \| \| 261.73 \| \| 316.05 \| \| 198.24 \| \| 113.92 \| \| 65.8 \| \| 110.93 \| \| 367.31 \| \| 401.58 \| \| 3,835.60 \| \| 0 \| \| 0 \| \| 3,521.02 \| \| 0 \| \| 190.63 \| \| 0 \| \| 0 \| \| 3,495.78 \| \| 0 \| \| 154.35 \| \| 46.34 \| \| 219.08 \| \| 266.93 \| \| 58.8 \| \| 664.91 \| \| 30.18 \| \| 25.59 \| \| 246.34 \| \| 349.78 \| \| 135.59 \| \| 76.42 \| \| 38.21 \| \| 28.45 \| \| 81.22 \| \| 36.92 \| \| 18.12 \| \| 108.36 \| \| 170.59 \| \| 153.02 \| \| 84.16 \| \| 76.19 \| \| 76.17 \| \| 180.84 \| \| 54.43 \| \| 191.56 \| \| 1,022.59 \| \| 281.79 \| \| 68.24 \| \| 112.37 \| \| 143.9 \| \| 69.59 \| \| 70.61 \| \| 98.68 \| \| 59.25 \| \| 14.97 \| \| 29.81 \| \| 53.9 \| \| 42.28 \| \| 39.28 \| \| 51.09 \| \| 60.07 \| \| 31.87 \| \| 32.67 \| \| 39.96 \| \| 112.75 \| \| 73.03 \| \| 139.67 \| \| 370.21 \| \| 149.46 \| \| 416.98 \| \| 308.67 \| \| 58.99 \| \| 106.07 \| \| 87.63 \| \| 55.53 \| \| 77.43 \| \| 8.4 \| \| 36.05 \| \| 80.99 \| \| 240.26 \| \| 21.72 \| \| 61.37 \| \| 103.34 \| \| 115.29 \| \| 1,295.15 \| \| 0 \| \| 0 \| \| 0 \| \| 0 \| \| 86.76 \| \| 11.65 \| \| 0 \| \| 103.16 \| \| 193.67 \| \| 50.59 \| \| 65.69 \| \| 0 \| \| 0 \| \| 40.24 \| \| 29.04 \| \| 7.43 \| \| 41.31 \| \| 64.39 \| \| 0 \| \| 0 \| \| 87.77 \| \| 139.1 \| \| 0 \| \| 110.9 \| \| 71.49 \| \| 77.62 \| \| 57.83 \| \| 30.18 \| \| 37.56 \| \| 61.94 \| \| 35.57 \| \| 14.89 \| \| 40.94 \| \| 24 \| \| 46.37 \| \| 55.77 \| \| 78.64 \| \| 51.52 \| \| 9.37 \| \| 13.76 \| \| 34.83 \| \| 10.87 \| \| 56.08 \| \| 113.87 \| \| 174.48 \| \| 257.57 \| \| 201.08 \| \| 475.88 \| \| 65.99 \| \| 33.38 \| \| 14.06 \| \| 71.08 \| \| 75.46 \| \| 72.6 \| \| 21.03 \| \| 13.05 \| \| 269.19 \| \| 87.55 \| \| 45.47 \| \| 14.95 \| \| 20.9 \| \| 117.21 \| \| 98.71 \| \| 113.16 \| \| 7.87 \| \| 67.96 \| \| 457.65 \| \| 43.4 \| \| 76.78 \| \| 132.68 \| \| 164.92 \| \| 91.63 \| \| 426.24 \| \| 350.04 \| \| 407.11 \| \| 144.97 \| \| 190.04 \| \| 89.42 \| \| 146.36 \| \| 145.57 \| \| 219.38 \| \| 179.22 \| \| 209.96 \| \| 38.1 \| \| 38.65 \| \| 34.41 \| \| 78.24 \| \| 261.33 \| \| 336.69 \| \| 148.53 \| \| 168.68 \| \| 167.49 \| \| 366.25 \| \| 70.45 \| \| 56.11 \| \| 14.46 \| \| 15.16 \| \| 5.61 \| \| 41.77 \| \| 36.52  40.58 \| \| 131.7 \| \| 41.49 \| \| 1.27 \| \| 48.32 \| \| 98.89 \| \| 133.03 \| \| 32.02 \| \| 648.85 \| \| 702.65 \| \| 355.78 \| \| 76.15 \| \| 149.36 \| \| 186.93 \| \| 354.08 \| \| 225.93 \| \| 2,299.25 \| \| 587.35 \| \| 287.92 \| \| 0 \| \| 1.39 \| \| 0 \| \| 0 \| \| 0 \| \| 0 \| \| 0 \| \| 0 \| \| 0 \| \| 0 \| \| 0 \| \| 0 \| \| 0 \| \| 0 \| \| 0 \| \| 0 \| \| 0 \| \| 0 \| \| 0 \| \| 0 \| \| 0 \| \| 0 \| \| 0 \| \| 0 \| \| 0 \| \| 0 \| \| 943.67 \| \| 808.71 \| \| 572.38 \| \| 448.68 \| \| 408.01 \| \| 210.6 \| \| 0 \| \| 362.32 \| \| 498.9 \| \| 0 \| \| 561.08 \| \| 634.02 \| \| 220.47 \| \| 48.4 \| \| 130.47 \| \| 95.24 \| \| 75.77 \| \| 111.55 \| \| 81.36 \| \| 469.94 \| \| 472 \| \| 0 \| \| 162.09 \| \| 330.89 \| \| 54.53 \| \| 101.3 \| \| 70.82 \| \| 257.44 \| \| 125.26 \| \| 445.88 \| \| 131.69 \| \| 119.48 \| \| 0 \| \| 67.05 \| \| 108.13 \| \| 151.58 \| \| 23.41 \| \| 282.44 \| \| 51.33 \| \| 88.32 \| \| 118.32 \| \| 85.55 \| \| 160.24 \| \| 81.51 \| \| 49.34 \| \| 57.73 \| \| 84.59 \| \| 113.16 \| \| 52.53 \| \| 66.31 \| \| 127.19 \| \| 0 \| \| 19.46 \| \| 71.36 \| \| 202.89 \| \| 144.28 \| \| 27.42 \| \| 12,195.22 \| \| 2,327.21 \| \| 1,682.14 \| \| 550.94 \| \| 0 \| \| 0 \| \| 0 \| \| 0 \| \| 0 \| \| 105.34 \| \| 709.92 \| \| 144.64 \| \| 1,080.57 \| \| 164.43 \| \| 29.48 \| \| 0 \| \| 91.07 \| \| 184.03 \| \| 0 \| \| 235.7 \| \| 41.98 \| \| 246.03 \| \| 273.17 \| \| 335.83 \| \| 346.3 \| \| 104.44 \| \| 0 \| \| 0 \| \| 0 \| \| 0 \| \| 0 \| \| 14.06 \| \| 56.93 \| \| 15.64 \| \| 43.82 \| \| 267.24 \| \| 116.88 \| \| 164.39 \| \| 116.82 \| \| 921.91 \| \| 630.11 \| \| 118.82 \| \| 304.29 \| \| 255.25 \| \| 220.47 \| \| 171.23 \| \| 171.25 \| \| 234.9 \| \| 403.29 \| \| 358.66 \| \| 206.11 \| \| 524.87 \| \| 1,263.50 \| \| 510.27 \| \| 1,106.74 \| \| 1,187.48 \| \| 514.62 \| \| 121.65 \| \| 90.66 \| \| 108.18 \| \| 14.09 \| \| 31.63 \| \| 32.69 \| \| 30.25 \| \| 120.55 \| \| 75.19 \| \| 133.04 \| \| 2,060.48 \| \| 79.02 \| \| 60.46 \| \| 124.38 \| \| 622.25 \| \| 14.32 \| \| 9.69 \| \| 49.47 \| \| 0 \| \| 0 \| \| 66.01 \| \| 122.41 \| \| 113.04 \| \| 0 \| \| 0 \| \| 172.41 \| \| 133.19 \| \| 245.4 \| \| 32.18 \| \| 25.63 \| \| 10.78 \| \| 24.5 \| \| 24.41 \| \| 53.4 \| \| 72.2 \| \| 1,004.39 \| \| 110.34 \| \| 106.04 \| \| 87.38 \| \| 222.5 \| \| 16.16 \| \| 0 \| \| 0 \| \| 65.1 \| \| 51.37 \| \| 42.47 \| \| 237.08 \| \| 295.33 \| \| 91.37 \| \| 37.56 \| \| 190.23 \| \| 186.96 \| \| 148.83 \| \| 93.95 \| \| 852.53 \| \| 230.2 \| \| 101.6 \| \| 98.48 \| \| 212.28 \| \| 338.91 \| \| 241.04 \| \| 58.8 \| \| 75.4 \| \| 88.2 \| \| 39.28 \| \| 62.31 \| \| 261.45 \| \| 79.52 \| \| 293.74 \| \| 0 \| \| 0 \| \| 301.09 \| \| 105.4 \| \| 231.38 \| \| 307.97 \| \| 1,731.66 \| \| 96.49 \| \| 445.96 \| \| 65.76 \| \| 25.98 \| \| 68.89 \| \| 215.02 \| \| 133.37 \| \| 212.82 \| \| 251.53 \| \| 224.28 \| \| 85.4 \| \| 45.49 \| \| 41.59 \| \| 98.73 \| \| 142.11 \| \| 175.56 \| \| 333.94 \| \| 473.13 \| \| 95.37 \| \| 318.74 \| \| 26.95 \| \| 121.95 \| \| 82.18 \| \| 269.5 \| \| 108.21 \| \| 87.03 \| \| 155.58 \| \| 203.51 \| \| 215.98 \| \| 255.29 \| \| 342.7 \| \| 0 \| \| 508.68 \| \| 2,011.55 \| \| 202.75 \| \| 256.76 \| \| 0 \| \| 0 \| \| 0 \| \| 0 \| \| 0 \| \| 66.24 \| \| 118.9 \| \| 88.23 \| \| 0 \| \| 0 \| \| 0 \| \| 7,887.51 \| \| 0 \| \| 667.1 \| \| 1,208.85 \| \| 2,346.70 \| \| 2,585.79 \| \| 2,604.64 \| \| 4,412.74 \| \| 1,053.15 \| \| 1,208.39 \| \| 2,287.06 \| \| 3,255.80 \| \| 2,614.20 \| \| 3,045.79 \| \| 3,054.65 \| \| 3,405.85 \| \| 3,132.57 \| \| 1,431.57 \| \| 3,405.43 \| \| 1,633.75 \| \| 1,144.18 \| \| 2,947.53 \| \| 1,577.14 \| \| 820.66 \| \| 1,800.24 \| \| 1,688.85 \| \| 1,483.76 \| \| 3,098.18 \| \| 1,446.83 \| \| 1,999.55 \| \| 2,268.70 \| \| 1,320.08 \| \| 2,252.46 \| \| 559.21 \| \| 1,785.24 \| \| 512.04 \| \| 2,035.50 \| \| 1,778.68 \| \| 3,405.42 \| \| 2,144.32 \| \| 854.81 \| \| 169.03 \| \| 46.83 \| \| 94.79 \| \| 34.06 \| \| 160.2 \| \| 116.09 \| \| 834.39 \| \| 1,678.11 \| \| 2,761.49 \| \| 108.24 \| \| 474.52 \| \| 250.25 \| \| 216.46 \| \| 431.72 \| \| 245.54 \| \| 53.82 \| \| 49.79 \| \| 145.85 \| \| 21,112.13 \| \| 378.89 \| \| 182.64 \| \| 385.96 \| \| 277.12 \| \| 157.05 \| \| 391.05 \| \| 221.13 \| \| 69.19 \| \| 121.78 \| \| 217.46 \| \| 158.19 \| \| 396.14 \| \| 137.59 \| \| 83.02 \| \| 93.61 \| \| 296.12 \| \| 7.19 \| \| 30.48 \| \| 19.68 \| \| 126.22 \| \| 81.16 \| \| 82.55 \| \| 83.43 \| \| 176.24 \| \| 157.96 \| \| 227.94 \| \| 1,530.10 \| \| 18.81 \| \| 49.81 \| \| 0 \| \| 381.34 \| \| 0 \| \| 0 \| \| 0 \| \| 0 \| \| 0 \| \| 0 \| \| 0 \| \| 997.89 \| \| 609.14 \| \| 1,062.95 \| \| 443.35 \| \| 2,038.70 \| \| 0 \| \| 0 \| \| 0 \| \| 0 \| \| 0 \| \| 388.37 \| \| 219.38 \| \| 350.87 \| \| 289.62 \| \| 136.35 \| \| 257.13 \| \| 110.55 \| \| 0 \| \| 189.17 \| \| 511.05 \| \| 262.24 \| \| 476.47 \| \| 283.39 \| \| 122.72 \| \| 19.14 \| \| 34.31 \| \| 27.68 \| \| 0 \| \| 0 \| \| 0 \| \| 0 \| \| 396.22 \| \| 523.88 \| \| 136.9 \| \| 313.52 \| \| 658.43 \| \| 37.48 \| \| 34.35 \| \| 138.31 \| \| 138.7 \| \| 69.79 \| \| 736.84 \| \| 734.69 \| \| 607.44 \| \| 390.72 \| \| 332.98 \| \| 289.49 \| \| 1,754.43 \| \| 487.78 \| \| 96.9 \| \| 119.98 \| \| 128.19 \| \| 129.43 \| \| 97.75 \| \| 0 \| \| 132.41 \| \| 86.6 \| \| 25.45 \| \| 66.34 \| \| 59.32 \| \| 82.24 \| \| 171.96 \| \| 162.91 \| \| 6.37 \| \| 47.98 \| \| 34.41 \| \| 35.33 \| \| 32.44 \| \| 14.23 \| \| 29.13 \| \| 14.54 \| \| 31.94 \| \| 19.35 \| \| 40.42 \| \| 214.76 \| \| 206.49 \| \| 102.13 \| \| 22.26 \| \| 150.14 \| \| 524.07 \| \| 268.86 \| \| 296.82 \| \| 77.86 \| \| 57.55 \| \| 44.49 \| \| 91.57 \| \| 91.14 \| \| 42.16 \| \| 20.21 \| \| 108.14 \| \| 80.38 \| \| 50.91 \| \| 69.45 \| \| 30.55 \| \| 43.6 \| \| 57.31 \| \| 49.26 \| \| 41.16 \| \| 152.41 \| \| 79.37 \| \| 72.73 \| \| 242.74 \| \| 92.54 \| \| 400.97 \| \| 393 \| \| 142.64 \| \| 47.07 \| \| 0 \| \| 7.59 \| \| 100.53 \| \| 56.86 \| \| 74.12 \| \| 519.03 \| \| 147.65 \| \| 371.69 \| \| 0 \| \| 109.55 \| \| 70.76 \| \| 156.77 \| \| 203.62 \| \| 242.75 \| \| 259.45 \| \| 131.26 \| \| 334.82 \| \| 3,522.79 \| \| 170.34 \| \| 180.11 \| \| 269.5 \| \| 110.61 \| \| 270.38 \| \| 203.33 \| \| 197.4 \| \| 345.52 \| \| 131.19 \| \| 218.43 \| \| 158.16 \| \| 53.41 \| \| 37.07 \| \| 70.76 \| \| 335.92 \| \| 201.21 \| \| 151.19 \| \| 130.54 \| \| 32.29 \| \| 65.88 \| \| 104.21 \| \| 661.3 \| \| 15,862.51 \| \| 114.05 \| \| 133.42 \| \| 115.23 \| \| 36.27 \| \| 54.7 \| \| 71.2 \| \| 53.59 \| \| 32.08 \| \| 14.69 \| \| 18.21 \| \| 33.39 \| \| 50.76 \| \| 73.32 \| \| 84.64 \| \| 101.97 \| \| 161.7 \| \| 19.37 \| \| 75.64 \| \| 71.7 \| \| 111.18 \| \| 55.9 \| \| 161.26 \| \| 165.95 \| \| 208 \| \| 104.82 \| \| 216.14 \| \| 131.55 \| \| 195.71 \| \| 6,348.79 \| \| 219.34 \| \| 92.35 \| \| 191.15 \| \| 235.79 \| \| 176.16  135.09 \| \| 112.15 \| \| 96.77 \| \| 578.81 \| \| 31.45 \| \| 19.72 \| \| 10.09 \| \| 34.93 \| \| 33.47 \| \| 32.55 \| \| 86.07 \| \| 51.47 \| \| 14.65 \| \| 0 \| \| 18.49 \| \| 13.25 \| \| 43.24 \| \| 566.09 \| \| 53.54 \| \| 66.18 \| \| 34.81 \| \| 33.23 \| \| 17.42 \| \| 49.03 \| \| 37.95 \| \| 28.41 \| \| 107.5 \| \| 82.77 \| \| 28.87 \| \| 70.29 \| \| 74.73 \| \| 21.69 \| \| 14.13 \| \| 27.7 \| \| 77.33 \| \| 152.37 \| \| 77.24 \| \| 54.78 \| \| 11.5 \| \| 61.99 \| \| 131.98 \| \| 31.6 \| \| 7.04 \| \| 13.76 \| \| 17.63 \| \| 11.53 \| \| 15.68 \| \| 9.48 \| \| 53.24 \| \| 32 \| \| 50.07 \| \| 65.19 \| \| 364.67 \| \| 26.11 \| \| 176.97 \| \| 30.26 \| \| 27.39 \| \| 37.45 \| \| 1.01 \| \| 33.95 \| \| 51.59 \| \| 41.31 \| \| 108.19 \| \| 18.31 \| \| 55.14 \| \| 19.6 \| \| 0 \| \| 54.26 \| \| 152.55 \| \| 50.43 \| \| 93.24 \| \| 12.1 \| \| 21.99 \| \| 134.63 \| \| 316.18 \| \| 57.05 \| \| 99.2 \| \| 234.18 \| \| 893.93 \| \| 430.38 \| \| 529.24 \| \| 34.58 \| \| 140.04 \| \| 1,180.37 \| \| 43.47 \| \| 67.5 \| \| 32.67 \| \| 7.19 \| \| 28.75 \| \| 118.8 \| \| 78.67 \| \| 176.28 \| \| 107.8 \| \| 20.18 \| \| 49.39 \| \| 108.43 \| \| 234.81 \| \| 183.13 \| \| 175.81 \| \| 91.76 \| \| 199.74 \| \| 240.12 \| \| 2,150.74 \| \| 2,285.83 \| \| 318.18 \| \| 0 \| \| 3.34 \| \| 0.98 \| \| 2.75 \| \| 2.24 \| \| 1.75 \| \| 5.33 \| \| 3.55 \| \| 0.84 \| \| 3.73 \| \| 6.51 \| \| 3.72 \| \| 0 \| \| 0 \| \| 0 \| \| 0 \| \| 0 \| \| 0 \| \| 0 \| \| 0 \| \| 0 \| \| 0 \| \| 0 \| \| 0 \| \| 0 \| \| 0 \| \| 6.25 \| \| 0 \| \| 5.61 \| \| 7.39 \| \| 3.67 \| \| 12.65 \| \| 7.37 \| \| 4.45 \| \| 2.41 \| \| 0.92 \| \| 0 \| \| 44.15 \| \| 15.29 \| \| 0 \| \| 0 \| \| 0 \| \| 1.71 \| \| 3.66 \| \| 4.67 \| \| 4.9 \| \| 0 \| \| 0 \| \| 0 \| \| 0 \| \| 0 \| \| 0 \| \| 65.88 \| \| 2,015.84 \| \| 63.88 \| \| 273.93 \| \| 287.2 \| \| 121.35 \| \| 303.33 \| \| 43.6 \| \| 22.34 \| \| 71.42 \| \| 59.14 \| \| 50.94 \| \| 121.48 \| \| 129.26 \| \| 78.4 \| \| 80.45 \| \| 0 \| \| 0 \| \| 0 \| \| 28.46 \| \| 99.08 \| \| 161.24 \| \| 51.56 \| \| 91.95 \| \| 1,365.97 \| \| 1,240.98 \| \| 987.9 \| \| 373.51 \| \| 28.29 \| \| 379.29 \| \| 131.31 \| \| 202.98 \| \| 120.63 \| \| 92.75 \| \| 359.09 \| \| 87.38 \| \| 110.98 \| \| 1,354.30 \| \| 281.79 \| \| 158.93 \| \| 1,999.91 \| \| 136.66 \| \| 142.74 \| \| 85.77 \| \| 46.48 \| \| 116.52 \| \| 78.3 \| \| 180.95 \| \| 200.7 \| \| 99.56 \| \| 0 \| \| 0 \| \| 3.71 \| \| 14.87 \| \| 0 \| \| 0 \| \| 0 \| \| 0 \| \| 0 \| \| 37.39 \| \| 432.95 \| \| 114.39 \| \| 56.08 \| \| 88.98 \| \| 107.33 \| \| 29.2 \| \| 21.56 \| \| 86.89 \| \| 34.92 \| \| 53.9 \| \| 29.23 \| \| 39.17 \| \| 76.64 \| \| 34.73 \| \| 17.74 \| \| 14.59 \| \| 9.88 \| \| 52.99 \| \| 29.29 \| \| 49.54 \| \| 52.62 \| \| 46.13 \| \| 31.31 \| \| 65.97 \| \| 292.91 \| \| 488.89 \| \| 101.57 \| \| 684.31 \| \| 149.65 \| \| 50.24 \| \| 236.97 \| \| 776.51 \| \| 119.48 \| \| 47.11 \| \| 44.49 \| \| 25.72 \| \| 23.61 \| \| 83.44 \| \| 739.85 \| \| 282.64 \| \| 105 \| \| 62.4 \| \| 123.34 \| \| 27.83 \| \| 51.56 \| \| 116.78 \| \| 280.23 \| \| 17.82 \| \| 34.84 \| \| 44.83 \| \| 28.53 \| \| 171.14 \| \| 126.75 \| \| 6.2 \| \| 69.65 \| \| 1,375.05 \| \| 122.37 \| \| 511.51 \| \| 50.03 \| \| 78.86 \| \| 212.47 \| \| 260.89 \| \| 143.1 \| \| 112.57 \| \| 32.38 \| \| 56.81 \| \| 81.06 \| \| 112.56 \| \| 49.83 \| \| 17.53 \| \| 167.69 \| \| 101.46 \| \| 96.73 \| \| 38.5 \| \| 22.24 \| \| 21.93 \| \| 0.64 \| \| 20.8 \| \| 67.78 \| \| 16.79 \| \| 50.63 \| \| 111.51 \| \| 23.53 \| \| 100 \| \| 466.57 \| \| 156.02 \| \| 204.52 \| \| 102.99 \| \| 173.5 \| \| 335.65 \| \| 352.28 \| \| 280.72 \| \| 588.79 \| \| 161.1 \| \| 156.4 \| \| 158.41 \| \| 328.03 \| \| 837.34 \| \| 365.98 \| \| 590.9 \| \| 1,415.41 \| \| 1,843.80 \| \| 21.74 \| \| 24.98 \| \| 13.09 \| \| 16.43 \| \| 13.74 \| \| 17.66 \| \| 933.15 \| \| 162.19 \| \| 308.78 \| \| 251.83 \| \| 271.22 \| \| 47.97 \| \| 80.56 \| \| 75.57 \| \| 1,310.78 \| \| 463.33 \| \| 212.29 \| \| 107.8 \| \| 90.7 \| \| 105.61 \| \| 105.76 \| \| 9.27 \| \| 0 \| \| 28.03 \| \| 22.73 \| \| 0 \| \| 0 \| \| 343.44 \| \| 62.94 \| \| 148.66 \| \| 45.65 \| \| 37.97 \| \| 36.37 \| \| 212.05 \| \| 96.79 \| \| 80.02 \| \| 317.93 \| \| 9.29 \| \| 11.55 \| \| 19.94 \| \| 35.63 \| \| 37.46 \| \| 45.23 \| \| 21.34 \| \| 34.04 \| \| 127.99 \| \| 19.72 \| \| 38.16 \| \| 64.21 \| \| 51.16 \| \| 153.64 \| \| 1,868.05 \| \| 158.73 \| \| 242.1 \| \| 102.67 \| \| 106.57 \| \| 112.49 \| \| 20.58 \| \| 58.84 \| \| 49 \| \| 71.36 \| \| 224.43 \| \| 63.34 \| \| 62 \| \| 86.63 \| \| 627.42 \| \| 1,564.48 \| \| 81.33 \| \| 85.27 \| \| 92.89 \| \| 66.55 \| \| 180.37 \| \| 193.39 \| \| 463.83 \| \| 78.58 \| \| 160.13 \| \| 55.46 \| \| 52.56 \| \| 181.48 \| \| 94.84 \| \| 110.64 \| \| 324.5 \| \| 53.08 \| \| 28.14 \| \| 104.61 \| \| 263.51 \| \| 274.58 \| \| 132.13 \| \| 85.4 \| \| 82.81 \| \| 179.82 \| \| 32.81 \| \| 107.47 \| \| 106.39 \| \| 433.28 \| \| 214.83 \| \| 231.98 \| \| 480.01 \| \| 361.23 \| \| 102.71 \| \| 93.28 \| \| 217.5 \| \| 343.76 \| \| 578.14 \| \| 363.08 \| \| 210.87 \| \| 182.64 \| \| 287.12 \| \| 192.71 \| \| 1,660.88 \| \| 265.89 \| \| 329.66 \| \| 222.05 \| \| 339.98 \| \| 213.12 \| \| 357.71 \| \| 329.39 \| \| 0 \| \| 62.07 \| \| 249.45 \| \| 0 \| \| 0 \| \| 49.23 \| \| 255.31 \| \| 87.94 \| \| 68.77 \| \| 65.81 \| \| 57.27 \| \| 73.15 \| \| 64.73 \| \| 43.12 \| \| 255.11 \| \| 71.87 \| \| 162.16 \| \| 113.51 \| \| 114.9 \| \| 62.83 \| \| 175.32 \| \| 86.41 \| \| 203.22 \| \| 0 \| \| 17.82 \| \| 0 \| \| 31.37 \| \| 28.94 \| \| 21.74 \| \| 36.87 \| \| 33.06 \| \| 0 \| \| 0 \| \| 0 \| \| 0 \| \| 0 \| \| 18.35 \| \| 24.88 \| \| 15.81 \| \| 0 \| \| 0 \| \| 0 \| \| 0 \| \| 0 \| \| 0 \| \| 0 \| \| 0 \| \| 0 \| \| 0 \| \| 0 \| \| 0 \| \| 270.88 \| \| 110.2 \| \| 86.58 \| \| 164.16 \| \| 91.31 \| \| 38.56 \| \| 41.46 \| \| 136.49 \| \| 151.41 \| \| 191.81 \| \| 46.2 \| \| 193.77 \| \| 323.09 \| \| 400.88 \| \| 424.55 \| \| 1,990.15 \| \| 0 \| \| 938.97 \| \| 256.31 \| \| 249.89 \| \| 89.52 \| \| 64.78 \| \| 93.67 \| \| 264.2 \| \| 305.71 \| \| 106.18 \| \| 35.72 \| \| 85.26 \| \| 223.97 \| \| 390.45 \| \| 347.61 \| \| 28.08 \| \| 35.75 \| \| 217.14 \| \| 1,116.00 \| \| 546.18 \| \| 561.57 \| \| 159.84 \| \| 65.07 \| \| 668.3 \| \| 384.6 \| \| 179.51 \| \| 101.3 \| \| 128.48 \| \| 76.66 \| \| 594.14 \| \| 252.8 \| \| 238.21 \| \| 477.91 \| \| 160.38 \| \| 96.59 \| \| 319.17 \| \| 341.28 \| \| 916.04 \| \| 67.81 \| \| 26.33 \| \| 0 \| \| 169.81 \| \| 66.1 \| \| 455.8 \| \| 409.49 \| \| 160.08 \| \| 87.05 \| \| 208.86 \| \| 2,083.38 \| \| 453.54 \| \| 540.46 \| \| 137.92 \| \| 0 \| \| 78.57 \| \| 71.73 \| \| 184.13 \| \| 43.92 \| \| 76.14 \| \| 21.56 \| \| 21.5 \| \| 25.33 \| \| 29.79 \| \| 70.75 \| \| 100.32 \| \| 924.49 \| \| 923.37 \| \| 31.5 \| \| 41.7 \| \| 25.56 \| \| 21.41 \| \| 34.55 \| \| 70.15 \| \| 0 \| \| 0 \| \| 0 \| \| 98.03 \| \| 178.87  48.82 \| \| 174.14 \| \| 292 \| \| 395.49 \| \| 382.53 \| \| 413.11 \| \| 1,116.85 \| \| 275.49 \| \| 69.9 \| \| 0 \| \| 220.03 \| \| 118.45 \| \| 401.08 \| \| 274.12 \| \| 40.95 \| \| 284.35 \| \| 333.22 \| \| 468.47 \| \| 155.77 \| \| 410.5 \| \| 115.13 \| \| 290.46 \| \| 301.84 \| \| 145.92 \| \| 97.51 \| \| 153.36 \| \| 255.75 \| \| 131.69 \| \| 170.19 \| \| 923.29 \| \| 590.8 \| \| 1,473.25 \| \| 1,258.61 \| \| 97.41 \| \| 35.03 \| \| 303.87 \| \| 245.49 \| \| 155 \| \| 86.24 \| \| 105.79 \| \| 271.83 \| \| 104.56 \| \| 526.35 \| \| 41.33 \| \| 268.23 \| \| 2,602.95 \| \| 291.29 \| \| 323.77 \| \| 375.84 \| \| 53.9 \| \| 113.19 \| \| 173.22 \| \| 242.68 \| \| 36.61 \| \| 31.5 \| \| 24.35 \| \| 29.97 \| \| 22.75 \| \| 47.76 \| \| 736.94 \| \| 125.35 \| \| 11.35 \| \| 26.01 \| \| 31.9 \| \| 14.07 \| \| 15.4 \| \| 17.09 \| \| 48.77 \| \| 19.16 \| \| 123.3 \| \| 0 \| \| 0 \| \| 32.57 \| \| 73.72 \| \| 88.93 \| \| 98.7 \| \| 103.2 \| \| 0 \| \| 0 \| \| 0 \| \| 0 \| \| 0 \| \| 0 \| \| 0 \| \| 0 \| \| 0 \| \| 0 \| \| 0 \| \| 0 \| \| 0 \| \| 0 \| \| 0 \| \| 0 \| \| 0 \| \| 0 \| \| 0 \| \| 0 \| \| 0 \| \| 0 \| \| 0 \| \| 0 \| \| 0 \| \| 0 \| \| 0 \| \| 0 \| \| 0 \| \| 0 \| \| 0 \| \| 0 \| \| 0 \| \| 0 \| \| 0 \| \| 0 \| \| 0 \| \| 0 \| \| 0 \| \| 0 \| \| 0 \| \| 0 \| \| 0 \| \| 0 \| \| 0 \| \| 0 \| \| 0 \| \| 0 \| \| 0 \| \| 0 \| \| 0 \| \| 0 \| \| 0 \| \| 0 \| \| 0 \| \| 0 \| \| 0 \| \| 0 \| \| 0 \| \| 0 \| \| 0 \| \| 0 \| \| 0 \| \| 0 \| \| 0 \| \| 0 \| \| 0 \| \| 0 \| \| 0 \| \| 0 \| \| 0 \| \| 0 \| \| 0 \| \| 0 \| \| 0 \| \| 0 \| \| 0 \| \| 0 \| \| 0 \| \| 0 \| \| 0 \| \| 0 \| \| 0 \| \| 0 \| \| 0 \| \| 0 \| \| 0 \| \| 0 \| \| 0 \| \| 0 \| \| 0 \| \| 0 \| \| 0 \| \| 0 \| \| 0 \| \| 0 \| \| 0 \| \| 0 \| \| 0 \| \| 0 \| \| 0 \| \| 0 \| \| 0 \| \| 0 \| \| 0 \| \| 0 \| \| 0 \| \| 0 \| \| 0 \| \| 0 \| \| 0 \| \| 0 \| \| 0 \| \| 0 \| \| 0 \| \| 0 \| \| 0 \| \| 0 \| \| 0 \| \| 0 \| \| 0 \| \| 0 \| \| 0 \| \| 0 \| \| 0 \| \| 0 \| \| 0 \| \| 0 \| \| 0 \| \| 0 \| \| 0 \| \| 82.4 \| \| 378.58 \| \| 264.18 \| \| 114.07 \| \| 1,022.03 \| \| 490.64 \| \| 23.5 \| \| 84.9 \| \| 228.44 \| \| 86.24 \| \| 659.33 \| \| 1,797.60 \| \| 245.49 \| \| 16.17 \| \| 22.1 \| \| 147.29 \| \| 93.43 \| \| 49.75 \| \| 69.49 \| \| 152.76 \| \| 38.16 \| \| 65.33 \| \| 70.93 \| \| 195.45 \| \| 956.15 \| \| 92.66 \| \| 184.43 \| \| 791.13 \| \| 554.81 \| \| 531.41 \| \| 418.88 \| \| 159.67 \| \| 90.61 \| \| 214.42 \| \| 28.04 \| \| 87.3 \| \| 603.67 \| \| 60.44 \| \| 107.18 \| \| 155.96 \| \| 137.16 \| \| 51.5 \| \| 50.95 \| \| 277.05 \| \| 275.8 \| \| 297.69 \| \| 35.37 \| \| 24.52 \| \| 26.71 \| \| 185.7 \| \| 143.73 \| \| 458.15 \| \| 307.82 \| \| 279.3 \| \| 105.23 \| \| 279.51 \| \| 3,803.84 \| \| 262.1 \| \| 172.48 \| \| 285 \| \| 125.11 \| \| 198.53 \| \| 338.85 \| \| 567.08 \| \| 506.15 \| \| 166.64 \| \| 790.27 \| \| 404.11 \| \| 178.57 \| \| 437.12 \| \| 816.84 \| \| 1,017.15 \| \| 314.14 \| \| 29.32 \| \| 21.36 \| \| 27.35 \| \| 120.6 \| \| 0 \| \| 57.16 \| \| 3.8 \| \| 189.11 \| \| 864.17 \| \| 283.13 \| \| 0 \| \| 0 \| \| 0 \| \| 0 \| \| 43.5 \| \| 51.43 \| \| 25.79 \| \| 38.7 \| \| 42.45 \| \| 116.47 \| \| 27.48 \| \| 23 \| \| 94.48 \| \| 0 \| \| 205.86 \| \| 50.5 \| \| 141.84 \| \| 74.54 \| \| 341.67 \| \| 88 \| \| 18.72 \| \| 2.76 \| \| 41.63 \| \| 12.26 \| \| 16.57 \| \| 10.56 \| \| 7.46 \| \| 16.53 \| \| 13.72 \| \| 10.86 \| \| 4.1 \| \| 18.34 \| \| 14.55 \| \| 22.39 \| \| 0 \| \| 1.3 \| \| 39.34 \| \| 194.46 \| \| 66.48 \| \| 0 \| \| 0 \| \| 36.08 \| \| 0 \| \| 0 \| \| 0 \| \| 14.08 \| \| 28.85 \| \| 48.15 \| \| 34.37 \| \| 182.98 \| \| 73.26 \| \| 279.85 \| \| 277.77 \| \| 163.59 \| \| 193.74 \| \| 230.61 \| \| 1,021.03 \| \| 0 \| \| 54.62 \| \| 19.47 \| \| 52.46 \| \| 152.43 \| \| 970.97 \| \| 137.2 \| \| 121.27 \| \| 146.77 \| \| 88.41 \| \| 55.27 \| \| 35.93 \| \| 52.19 \| \| 0 \| \| 27.2 \| \| 25.84 \| \| 35.93 \| \| 67.87 \| \| 129.93 \| \| 126.77 \| \| 115.96 \| \| 69.97 \| \| 59.38 \| \| 74.38 \| \| 277.01 \| \| 329.89 \| \| 551.85 \| \| 366.3 \| \| 220.34 \| \| 142.94 \| \| 125.56 \| \| 57.39 \| \| 39.16 \| \| 65.76 \| \| 153.63 \| \| 217.77 \| \| 64.68 \| \| 155.85 \| \| 140.49 \| \| 70.25 \| \| 107.36 \| \| 65.27 \| \| 38.3 \| \| 306.81 \| \| 361.01 \| \| 287.86 \| \| 109.73 \| \| 122.57 \| \| 114.62 \| \| 138.64 \| \| 56.6 \| \| 280.18 \| \| 260.8 \| \| 211.06 \| \| 90.11 \| \| 108.44 \| \| 64.77 \| \| 168.71 \| \| 109.38 \| \| 92.64 \| \| 124.33 \| \| 108.88 \| \| 68.03 \| \| 137.23 \| \| 35.11 \| \| 16.37 \| \| 18.17 \| \| 13.99 \| \| 914.19 \| \| 412.37 \| \| 316.69 \| \| 185.67 \| \| 101.36 \| \| 293.48 \| \| 133.16 \| \| 89.95 \| \| 48.19 \| \| 59.5 \| \| 33.34 \| \| 22.05 \| \| 46.37 \| \| 305.43 \| \| 607.12 \| \| 466.1 \| \| 686.12 \| \| 223 \| \| 90.4 \| \| 182.41 \| \| 46.96 \| \| 0 \| \| 5.2 \| \| 704.23 \| \| 90.45 \| \| 82.13 \| \| 102.67 \| \| 720.37 \| \| 159.43 \| \| 61.34 \| \| 108.91 \| \| 700.4 \| \| 206.76 \| \| 183.98 \| \| 181.02 \| \| 27.94 \| \| 620.84 \| \| 116.16 \| \| 241.47 \| \| 229.27 \| \| 1,239.06 \| \| 23.96 \| \| 27.2 \| \| 43.66 \| \| 39 \| \| 27.88 \| \| 53.69 \| \| 0 \| \| 89.04 \| \| 112.85 \| \| 94.65 \| \| 77.3 \| \| 26.88 \| \| 148.22 \| \| 144.03 \| \| 222.92 \| \| 64.15 \| \| 68.97 \| \| 39.75 \| \| 96.02 \| \| 63.44 \| \| 74.71 \| \| 74.88 \| \| 65.64 \| \| 83.4 \| \| 54.2 \| \| 16.25 \| \| 17.73 \| \| 83.25 \| \| 29.37 \| \| 11.28 \| \| 13.18 \| \| 34.83 \| \| 25.41 \| \| 56.79 \| \| 56.31 \| \| 45.75 \| \| 36.12 \| \| 39.39 \| \| 29.17 \| \| 30.73 \| \| 47.12 \| \| 44.18 \| \| 41.53 \| \| 0 \| \| 0 \| \| 11.5 \| \| 8.08 \| \| 333.06 \| \| 213.89 \| \| 197.25 \| \| 180.35 \| \| 54.74 \| \| 19.8 \| \| 79.88 \| \| 128.77 \| \| 683.34 \| \| 154.08 \| \| 154.68 \| \| 48.93 \| \| 62.24 \| \| 32.9 \| \| 31.97 \| \| 60.67 \| \| 72.83 \| \| 52.17 \| \| 85.87 \| \| 61.94 \| \| 43.6 \| \| 47.37 \| \| 61.26 \| \| 77.28 \| \| 777.84 \| \| 218.46 \| \| 203.12 \| \| 112.36 \| \| 67.66 \| \| 36.56 \| \| 33.17 \| \| 0.71 \| \| 61.75 \| \| 124.91 \| \| 226.64 \| \| 194.09 \| \| 245.7 \| \| 481.32 \| \| 226.48 \| \| 83.14 \| \| 444.92 \| \| 33.69 \| \| 248.09 \| \| 72.01 \| \| 46.38 \| \| 89.69 \| \| 464.13 \| \| 416.4 \| \| 350.68 \| \| 301.13 \| \| 344.77 \| \| 44.99 \| \| 53.9 \| \| 0 \| \| 0 \| \| 22.63 \| \| 18.32 \| \| 21.44 \| \| 11.46 \| \| 10.61 \| \| 183.26 \| \| 7.19 \| \| 104.15 \| \| 177.69 \| \| 515.89 \| \| 0 \| \| 0 \| \| 13.93 \| \| 13.72 \| \| 14.81 \| \| 10.07 \| \| 75.2 \| \| 23.16 \| \| 18.98 \| \| 18.7 \| \| 8.42 \| \| 75.67 \| \| 36.41 \| \| 49 \| \| 101.39 \| \| 168.83 \| \| 135.25  63.8 \| \| 44.39 \| \| 42.12 \| \| 199.43 \| \| 0 \| \| 0 \| \| 0 \| \| 0 \| \| 0 \| \| 0 \| \| 0 \| \| 0 \| \| 0 \| \| 0 \| \| 0 \| \| 0 \| \| 0 \| \| 0 \| \| 0 \| \| 0 \| \| 0 \| \| 0 \| \| 0 \| \| 0 \| \| 0 \| \| 1.59 \| \| 1.84 \| \| 0 \| \| 10.11 \| \| 0 \| \| 0 \| \| 0 \| \| 0 \| \| 0 \| \| 0 \| \| 0 \| \| 0 \| \| 0 \| \| 0 \| \| 0 \| \| 0 \| \| 0 \| \| 0 \| \| 0 \| \| 0 \| \| 0 \| \| 0 \| \| 0 \| \| 0 \| \| 0 \| \| 0 \| \| 0 \| \| 0 \| \| 0 \| \| 0 \| \| 0 \| \| 0 \| \| 0 \| \| 0 \| \| 0 \| \| 0 \| \| 0 \| \| 0 \| \| 0 \| \| 0 \| \| 0 \| \| 0 \| \| 0 \| \| 0 \| \| 0 \| \| 0 \| \| 0 \| \| 0 \| \| 0 \| \| 0 \| \| 0 \| \| 0 \| \| 0 \| \| 0 \| \| 0 \| \| 0 \| \| 0 \| \| 0 \| \| 0 \| \| 0 \| \| 0 \| \| 0 \| \| 0 \| \| 0 \| \| 0 \| \| 0 \| \| 0 \| \| 0 \| \| 0 \| \| 0 \| \| 0 \| \| 0 \| \| 0 \| \| 0 \| \| 0 \| \| 0 \| \| 0 \| \| 0 \| \| 0 \| \| 0 \| \| 0 \| \| 9.61 \| \| 0 \| \| 22.24 \| \| 39.42 \| \| 17.58 \| \| 6.18 \| \| 14.96 \| \| 26.95 \| \| 78.32 \| \| 62.23 \| \| 117.47 \| \| 0 \| \| 12.53 \| \| 17.26 \| \| 7.83 \| \| 1.05 \| \| 8.08 \| \| 7.99 \| \| 29.33 \| \| 16.15 \| \| 29.83 \| \| 0 \| \| 0 \| \| 0 \| \| 0 \| \| 0 \| \| 0 \| \| 30.44 \| \| 17.58 \| \| 16.83 \| \| 87.68 \| \| 19.6 \| \| 17.76 \| \| 1.83 \| \| 11.04 \| \| 17.16 \| \| 53.48 \| \| 14.51 \| \| 37.93 \| \| 17.08 \| \| 22.3 \| \| 12.34 \| \| 6.48 \| \| 11.3 \| \| 9.35 \| \| 12.36 \| \| 15.11 \| \| 17.32 \| \| 38.06 \| \| 6.84 \| \| 8.59 \| \| 10.03 \| \| 14.7 \| \| 12.44 \| \| 19.39 \| \| 92.02 \| \| 87.1 \| \| 33.12 \| \| 26.57 \| \| 41 \| \| 63.17 \| \| 62.45 \| \| 41.5 \| \| 40.33 \| \| 172.43 \| \| 58.22 \| \| 165.41 \| \| 535 \| \| 62.04 \| \| 13.7 \| \| 27.05 \| \| 23.27 \| \| 30.44 \| \| 19.89 \| \| 69.84 \| \| 52.37 \| \| 40.5 \| \| 154.88 \| \| 19.72 \| \| 14.12 \| \| 34.2 \| \| 111.69 \| \| 28.05 \| \| 39.68 \| \| 51.87 \| \| 85.16 \| \| 66.83 \| \| 26.46 \| \| 22.85 \| \| 21.77 \| \| 90.87 \| \| 61.02 \| \| 39.14 \| \| 101.09 \| \| 48.3 \| \| 11.86 \| \| 183.94 \| \| 304.71 \| \| 28.21 \| \| 39.72 \| \| 27.42 \| \| 20.57 \| \| 27.49 \| \| 22.32 \| \| 19.66 \| \| 74.76 \| \| 33.74 \| \| 38.6 \| \| 0 \| \| 22.72 \| \| 190.97 \| \| 35 \| \| 51.62 \| \| 63 \| \| 98.65 \| \| 23.61 \| \| 25.3 \| \| 24.69 \| \| 15.7 \| \| 79.66 \| \| 4.49 \| \| 7.85 \| \| 14.49 \| \| 22.48 \| \| 18.82 \| \| 74.03 \| \| 524.24 \| \| 223.72 \| \| 67.69 \| \| 0 \| \| 23.58 \| \| 20.96 \| \| 6.53 \| \| 27.47 \| \| 14.29 \| \| 17.32 \| \| 17.69 \| \| 15.16 \| \| 9.33 \| \| 25.04 \| \| 24.45 \| \| 29.9 \| \| 16.02 \| \| 32 \| \| 26.72 \| \| 74.31 \| \| 320.72 \| \| 94.13 \| \| 37.75 \| \| 105.86 \| \| 132.47 \| \| 6.47 \| \| 14.18 \| \| 28.05 \| \| 62.78 \| \| 48.36 \| \| 26.95 \| \| 0 \| \| 0 \| \| 45.21 \| \| 17.35 \| \| 24.02 \| \| 23.15 \| \| 37.66 \| \| 51.51 \| \| 25.29 \| \| 52.01 \| \| 113.9 \| \| 73.24 \| \| 109.72 \| \| 29.51 \| \| 54.63 \| \| 272.4 \| \| 13.71 \| \| 10.87 \| \| 9.37 \| \| 16.54 \| \| 16.9 \| \| 17.19 \| \| 11.51 \| \| 4.94 \| \| 65.9 \| \| 18.1 \| \| 18.76 \| \| 20.32 \| \| 10.98 \| \| 0 \| \| 0 \| \| 0 \| \| 0 \| \| 74.84 \| \| 12.61 \| \| 19.86 \| \| 9.65 \| \| 13.47 \| \| 93.43 \| \| 83.71 \| \| 346.08 \| \| 31.94 \| \| 97.72 \| \| 118.79 \| \| 113.58 \| \| 102.64 \| \| 335.69 \| \| 46.59 \| \| 95.82 \| \| 16.87 \| \| 19.45 \| \| 24.65 \| \| 28.52 \| \| 24.43 \| \| 115.81 \| \| 27.23 \| \| 47.96 \| \| 101.75 \| \| 92.06 \| \| 23.52 \| \| 13.06 \| \| 19.35 \| \| 32.03 \| \| 12.32 \| \| 11.93 \| \| 10.07 \| \| 24.5 \| \| 9.41 \| \| 8.91 \| \| 18.93 \| \| 1.41 \| \| 5.12 \| \| 6.32 \| \| 24.79 \| \| 13.76 \| \| 15.23 \| \| 26.34 \| \| 18.34 \| \| 13.92 \| \| 28.8 \| \| 32.62 \| \| 8.79 \| \| 22.42 \| \| 9.14 \| \| 9.5 \| \| 72.33 \| \| 33.58 \| \| 13.35 \| \| 25.92 \| \| 14.95 \| \| 19.81 \| \| 18.47 \| \| 24.57 \| \| 18.43 \| \| 76.89 \| \| 40.16 \| \| 51.72 \| \| 88.41 \| \| 179.09 \| \| 76.17 \| \| 0 \| \| 0 \| \| 0 \| \| 0 \| \| 0 \| \| 0 \| \| 0 \| \| 0 \| \| 0.88 \| \| 4.17 \| \| 72.94 \| \| 51.45 \| \| 190.07 \| \| 145.29 \| \| 155.27 \| \| 0 \| \| 0 \| \| 19.42 \| \| 33 \| \| 0 \| \| 0 \| \| 0 \| \| 0 \| \| 0 \| \| 9.23 \| \| 0 \| \| 75.93 \| \| 102.51 \| \| 14.27 \| \| 21.19 \| \| 33.98 \| \| 118.02 \| \| 83.44 \| \| 117.86 \| \| 32.26 \| \| 15.88 \| \| 38.55 \| \| 148.18 \| \| 71.22 \| \| 71.96 \| \| 45.04 \| \| 14.02 \| \| 11.75 \| \| 20.03 \| \| 18.53 \| \| 9.91 \| \| 22.48 \| \| 36.39 \| \| 33.59 \| \| 52.95 \| \| 23.84 \| \| 127.84 \| \| 33.06 \| \| 76.82 \| \| 91.89 \| \| 64.01 \| \| 165.55 \| \| 1,198.45 \| \| 857.44 \| \| 157.51 \| \| 0 \| \| 55.04 \| \| 41.23 \| \| 169.59 \| \| 138.63 \| \| 108.79 \| \| 297.14 \| \| 461.95 \| \| 616.24 \| \| 105.15 \| \| 24.29 \| \| 74.7 \| \| 243.36 \| \| 243.79 \| \| 0 \| \| 0 \| \| 0 \| \| 168.69 \| \| 580.1 \| \| 81.23 \| \| 227.54 \| \| 32.49 \| \| 45.6 \| \| 63.15 \| \| 100.22 \| \| 98.43 \| \| 19.21 \| \| 20.06 \| \| 42.51 \| \| 31.64 \| \| 9.37 \| \| 18.85 \| \| 13.56 \| \| 21.5 \| \| 31 \| \| 25.47 \| \| 25.87 \| \| 48.06 \| \| 14.17 \| \| 23.59 \| \| 15.81 \| \| 19.08 \| \| 609.2 \| \| 22.34 \| \| 13.3 \| \| 15.36 \| \| 13.47 \| \| 7.59 \| \| 20.18 \| \| 9.47 \| \| 2.91 \| \| 24.44 \| \| 13.35 \| \| 38.29 \| \| 38.23 \| \| 114.44 \| \| 46.99 \| \| 99.73 \| \| 49.47 \| \| 30.64 \| \| 216.61 \| \| 0 \| \| 26.8 \| \| 13.6 \| \| 64.1 \| \| 254.84 \| \| 469.45 \| \| 164.44 \| \| 64.64 \| \| 120 \| \| 71.87 \| \| 57.9 \| \| 14.99 \| \| 68.6 \| \| 76.61 \| \| 53.9 \| \| 16.71 \| \| 16.18 \| \| 29.2 \| \| 132.95 \| \| 26.76 \| \| 47.49 \| \| 34.83 \| \| 38.19 \| \| 19.37 \| \| 42.99 \| \| 20.51 \| \| 24.54 \| \| 20.89 \| \| 169.67 \| \| 65.33 \| \| 392.34 \| \| 241.86 \| \| 122.17 \| \| 57.96 \| \| 22.19 \| \| 19.89 \| \| 41.85 \| \| 57.73 \| \| 266.05 \| \| 576.91 \| \| 3,053.11 \| \| 10,843.75 \| \| 1,792.57 \| \| 1,554.97 \| \| 486.32 \| \| 181.41 \| \| 45.95 \| \| 44.85 \| \| 0 \| \| 0 \| \| 0 \| \| 0 \| \| 0 \| \| 0 \| \| 476.18 \| \| 61.5 \| \| 101.86 \| \| 0 \| \| 44.95 \| \| 36.46 \| \| 22.35 \| \| 68.83 \| \| 0 \| \| 9.61 \| \| 174.84 \| \| 364.84 \| \| 218.59 \| \| 31.14 \| \| 52.58 \| \| 92.05 \| \| 140.9 \| \| 102.54 \| \| 119.72 \| \| 40.99 \| \| 167.34 \| \| 158.45 \| \| 123.02 \| \| 180.19 \| \| 976.53 \| \| 64.61 \| \| 34.77 \| \| 114.44 \| \| 152.95 \| \| 62.22 \| \| 27.85  36.21 \| \| 314.54 \| \| 81.24 \| \| 97.59 \| \| 146.69 \| \| 270.49 \| \| 235.06 \| \| 85.77 \| \| 136.12 \| \| 280.39 \| \| 205.05 \| \| 36.5 \| \| 0 \| \| 250.23 \| \| 479.2 \| \| 334.89 \| \| 535.85 \| \| 875.48 \| \| 257.45 \| \| 343.53 \| \| 409.79 \| \| 417.93 \| \| 420.69 \| \| 406.6 \| \| 226.57 \| \| 83.98 \| \| 101.64 \| \| 65.11 \| \| 93.65 \| \| 25.32 \| \| 132.81 \| \| 0 \| \| 22.16 \| \| 4.07 \| \| 23.38 \| \| 39.29 \| \| 46.05 \| \| 66.18 \| \| 36.54 \| \| 362.6 \| \| 122.47 \| \| 101.02 \| \| 52.97 \| \| 87.87 \| \| 127.95 \| \| 0 \| \| 65.15 \| \| 0 \| \| 0 \| \| 0 \| \| 0 \| \| 0 \| \| 0 \| \| 0 \| \| 0 \| \| 0 \| \| 0 \| \| 0 \| \| 0 \| \| 0 \| \| 0 \| \| 0 \| \| 0 \| \| 0 \| \| 0 \| \| 0 \| \| 0 \| \| 0 \| \| 0 \| \| 0 \| \| 0 \| \| 0 \| \| 0 \| \| 0 \| \| 0 \| \| 0 \| \| 0 \| \| 0 \| \| 0 \| \| 0 \| \| 0 \| \| 0 \| \| 0 \| \| 0 \| \| 0 \| \| 0 \| \| 0 \| \| 0 \| \| 0 \| \| 0 \| \| 0 \| \| 0 \| \| 0 \| \| 0 \| \| 0 \| \| 0 \| \| 0 \| \| 0 \| \| 0 \| \| 0 \| \| 0 \| \| 0 \| \| 0 \| \| 94.21 \| \| 282.36 \| \| 94.05 \| \| 70.02 \| \| 380.96 \| \| 165.71 \| \| 182.86 \| \| 0 \| \| 115.46 \| \| 166.08 \| \| 219.47 \| \| 222.41 \| \| 71.48 \| \| 201.14 \| \| 79.33 \| \| 0 \| \| 76.75 \| \| 80.44 \| \| 100.98 \| \| 163.8 \| \| 88.89 \| \| 221.8 \| \| 0 \| \| 0 \| \| 60.99 \| \| 89.88 \| \| 339.6 \| \| 0 \| \| 0 \| \| 0 \| \| 13.47 \| \| 47.72 \| \| 33.17 \| \| 138.02 \| \| 33.27 \| \| 100.55 \| \| 87.65 \| \| 101.5 \| \| 101.14 \| \| 142.76 \| \| 55.96 \| \| 0 \| \| 0 \| \| 0 \| \| 0 \| \| 36.83 \| \| 841.78 \| \| 355.89 \| \| 221.36 \| \| 86.17 \| \| 86.11 \| \| 135.6 \| \| 84.23 \| \| 456.51 \| \| 240.4 \| \| 0 \| \| 0 \| \| 1,571.71 \| \| 709.79 \| \| 1,473.78 \| \| 144.13 \| \| 405.44 \| \| 565.89 \| \| 150.92 \| \| 137.57 \| \| 84.01 \| \| 238.52 \| \| 109.04 \| \| 231.58 \| \| 119.38 \| \| 142.16 \| \| 172.29 \| \| 118.93 \| \| 132.1 \| \| 92.34 \| \| 1,621.95 \| \| 123.53 \| \| 309.29 \| \| 297.52 \| \| 36.45 \| \| 82.66 \| \| 550.82 \| \| 674.61 \| \| 310.07 \| \| 137.99 \| \| 30.49 \| \| 229.84 \| \| 58.56 \| \| 572.77 \| \| 986.33 \| \| 121.55 \| \| 98.26 \| \| 95.22 \| \| 165.02 \| \| 126.03 \| \| 128.56 \| \| 133.69 \| \| 170.51 \| \| 281.05 \| \| 346.56 \| \| 415.21 \| \| 138.44 \| \| 1,309.35 \| \| 1,829.36 \| \| 1,671.00 \| \| 0 \| \| 10.63 \| \| 15.25 \| \| 26.13 \| \| 252.43 \| \| 102.03 \| \| 148.39 \| \| 155.46 \| \| 241.19 \| \| 284.68 \| \| 1,415.58 \| \| 101.89 \| \| 116.57 \| \| 295.56 \| \| 77.32 \| \| 472.36 \| \| 1,821.80 \| \| 44.58 \| \| 183.68 \| \| 69.97 \| \| 157.12 \| \| 42.91 \| \| 580.11 \| \| 18.39 \| \| 24.99 \| \| 33 \| \| 454.83 \| \| 125.32 \| \| 132.75 \| \| 0 \| \| 141.7 \| \| 381.27 \| \| 290.31 \| \| 94.32 \| \| 216.63 \| \| 163.76 \| \| 220.43 \| \| 5,143.17 \| \| 115.65 \| \| 103.11 \| \| 147.51 \| \| 594.51 \| \| 304.05 \| \| 269.88 \| \| 48.31 \| \| 95.53 \| \| 643.95 \| \| 54.08 \| \| 94.73 \| \| 74.96 \| \| 46.2 \| \| 65.35 \| \| 82.62 \| \| 198.88 \| \| 142.07 \| \| 74.99 \| \| 231.77 \| \| 216.37 \| \| 234.06 \| \| 469.51 \| \| 38.53 \| \| 28.77 \| \| 44.34 \| \| 196.27 \| \| 98.98 \| \| 101.33 \| \| 156.05 \| \| 101.29 \| \| 151.84 \| \| 39.36 \| \| 121.8 \| \| 76.47 \| \| 293.05 \| \| 135.11 \| \| 95.71 \| \| 169.4 \| \| 44.06 \| \| 358.94 \| \| 307.65 \| \| 6,253.15 \| \| 5,633.90 \| \| 190.72 \| \| 0 \| \| 0 \| \| 102.97 \| \| 35.4 \| \| 20.57 \| \| 14.06 \| \| 21.29 \| \| 93.56 \| \| 40.35 \| \| 31.85 \| \| 78.85 \| \| 82.04 \| \| 35.4 \| \| 70.81 \| \| 73.35 \| \| 188.85 \| \| 111 \| \| 13.47 \| \| 747.96 \| \| 272.67 \| \| 124.22 \| \| 58.05 \| \| 955.78 \| \| 57.07 \| \| 341.88 \| \| 212.17 \| \| 141.29 \| \| 71.09 \| \| 170.04 \| \| 184.8 \| \| 1,145.86 \| \| 133.89 \| \| 97.46 \| \| 208.67 \| \| 134.64 \| \| 317.54 \| \| 368.53 \| \| 62.14 \| \| 147.08 \| \| 373.62 \| \| 143.62 \| \| 89.72 \| \| 347.67 \| \| 461.2 \| \| 168.06 \| \| 156.8 \| \| 94.21 \| \| 88.38 \| \| 22.6 \| \| 75.71 \| \| 144.19 \| \| 132.35 \| \| 26.36 \| \| 147.06 \| \| 0 \| \| 147.37 \| \| 81.18 \| \| 50.08 \| \| 57.16 \| \| 38.77 \| \| 0 \| \| 0 \| \| 35.34 \| \| 103.73 \| \| 153.84 \| \| 487.04 \| \| 469.67 \| \| 94.13 \| \| 130.97 \| \| 23.83 \| \| 116.88 \| \| 26.26 \| \| 325.09 \| \| 61.18 \| \| 33.45 \| \| 97.72 \| \| 164.73 \| \| 133.41 \| \| 32.34 \| \| 58.47 \| \| 40.33 \| \| 0 \| \| 405.83 \| \| 728.72 \| \| 207.04 \| \| 182.15 \| \| 2,605.14 \| \| 3,180.07 \| \| 1,926.68 \| \| 395.66 \| \| 27.22 \| \| 20.82 \| \| 63.64 \| \| 49.37 \| \| 57.02 \| \| 0 \| \| 1,699.33 \| \| 859.91 \| \| 89.78 \| \| 304.03 \| \| 1,059.04 \| \| 823.25 \| \| 657.19 \| \| 0 \| \| 0 \| \| 531.47 \| \| 462.7 \| \| 573.28 \| \| 323.4 \| \| 465.65 \| \| 441.71 \| \| 573.84 \| \| 805.67 \| \| 741.81 \| \| 935.28 \| \| 721.46 \| \| 807.63 \| \| 1,019.72 \| \| 715.47 \| \| 0 \| \| 428.24 \| \| 429.48 \| \| 686.8 \| \| 80.63 \| \| 135.52 \| \| 261.96 \| \| 153.47 \| \| 249.79 \| \| 433.02 \| \| 74.26 \| \| 11.4 \| \| 78.59 \| \| 55.36 \| \| 0 \| \| 0 \| \| 151.61 \| \| 97.31 \| \| 117.6 \| \| 189.13 \| \| 542.14 \| \| 174.16 \| \| 231.12 \| \| 259.41 \| \| 108.3 \| \| 170.71 \| \| 214.67 \| \| 237.24 \| \| 238.15 \| \| 115.45 \| \| 18.16 \| \| 138.67 \| \| 83.26 \| \| 242.36 \| \| 50.05 \| \| 437.4 \| \| 386.28 \| \| 675.57 \| \| 675.89 \| \| 585.05 \| \| 0 \| \| 264.67 \| \| 87.55 \| \| 245.5 \| \| 213.2 \| \| 60.84 \| \| 81.81 \| \| 104.47 \| \| 36.19 \| \| 60 \| \| 350.18 \| \| 77.08 \| \| 243.58 \| \| 140.26 \| \| 157.25 \| \| 75.81 \| \| 55.24 \| \| 81.78 \| \| 161.51 \| \| 523.36 \| \| 95.98 \| \| 193.05 \| \| 234.61 \| \| 68.96 \| \| 247.11 \| \| 144.44 \| \| 206.4 \| \| 351.71 \| \| 127.08 \| \| 41.85 \| \| 0 \| \| 48.75 \| \| 310.02 \| \| 79.76 \| \| 224.01 \| \| 312.89 \| \| 322.17 \| \| 60.07 \| \| 243.11 \| \| 131.82 \| \| 93.88 \| \| 52.11 \| \| 125.48 \| \| 79.46 \| \| 31.68 \| \| 129.79 \| \| 36.54 \| \| 32.74 \| \| 249.1 \| \| 240.8 \| \| 117.65 \| \| 83.58 \| \| 425.62 \| \| 36.94 \| \| 81.87 \| \| 66.98 \| \| 34.22 \| \| 166.79 \| \| 141.77 \| \| 690.11 \| \| 41.08 \| \| 122 \| \| 150.21 \| \| 143.37 \| \| 49.05 \| \| 62.56 \| \| 107.25 \| \| 102.95 \| \| 267.18 \| \| 47.12 \| \| 94.38 \| \| 49.53 \| \| 24.02 \| \| 187.91 \| \| 160.73 \| \| 559.89 \| \| 8,764.81 \| \| 174.68 \| \| 54.1 \| \| 18.78 \| \| 25.74 \| \| 22.22 \| \| 111.33 \| \| 275.39 \| \| 81.55 \| \| 726.47 \| \| 135.6 \| \| 243.57 \| \| 150.15 \| \| 156.12 \| \| 137.74 \| \| 151.16 \| \| 378.85 \| \| 0 \| \| 0 \| \| 11.55 \| \| 79.5 \| \| 345.75 \| \| 222.25 \| \| 282.97 \| \| 165.97 \| \| 371.84 \| \| 1,144.31 \| \| 482.53 \| \| 739.49 \| \| 182.85 \| \| 92.83 \| \| 2,609.18 \| \| 531.95 \| \| 245.2 \| \| 185.93 \| \| 57.49 \| \| 88.15 \| \| 43.95 \| \| 72.12 \| \| 179.36 \| \| 68.75 \| \| 41.66 \| \| 26.85 \| \| 0 \| \| 149.44 \| \| 86.4 \| \| 46 \| \| 80.65 \| \| 803.19 \| \| 58.23 \| \| 93.85 \| \| 196.36  16.08 \| \| 38.12 \| \| 36.66 \| \| 51.25 \| \| 18.16 \| \| 45.87 \| \| 24.52 \| \| 17.65 \| \| 59.7 \| \| 568.42 \| \| 149.02 \| \| 12.17 \| \| 501.56 \| \| 571.79 \| \| 78.58 \| \| 286.88 \| \| 361.38 \| \| 482.75 \| \| 522.71 \| \| 30.63 \| \| 142.06 \| \| 124.18 \| \| 236.72 \| \| 0 \| \| 0 \| \| 272.65 \| \| 1,021.86 \| \| 298.8 \| \| 113.72 \| \| 0 \| \| 52.1 \| \| 38.7 \| \| 72.8 \| \| 301.3 \| \| 124.17 \| \| 26.95 \| \| 187.01 \| \| 227.22 \| \| 22.91 \| \| 201.86 \| \| 52.44 \| \| 63.07 \| \| 109.51 \| \| 56.57 \| \| 55.87 \| \| 310.42 \| \| 65.8 \| \| 433.53 \| \| 95.79 \| \| 63.29 \| \| 107.8 \| \| 209.96 \| \| 36.67 \| \| 232.93 \| \| 187.76 \| \| 69.3 \| \| 53.45 \| \| 135.38 \| \| 103.06 \| \| 215.6 \| \| 457.35 \| \| 1,994.55 \| \| 312.62 \| \| 113.08 \| \| 186.63 \| \| 27.64 \| \| 75.55 \| \| 38.68 \| \| 565.77 \| \| 36.22 \| \| 32.56 \| \| 2,465.36 \| \| 10.38 \| \| 1,632.50 \| \| 636.33 \| \| 208.26 \| \| 450.06 \| \| 64.68 \| \| 55.7 \| \| 137.83 \| \| 143.2 \| \| 258.03 \| \| 100.76 \| \| 173.53 \| \| 283.75 \| \| 456.56 \| \| 407.61 \| \| 457.39 \| \| 334.97 \| \| 440.1 \| \| 1,385.30 \| \| 148.74 \| \| 78.57 \| \| 182.08 \| \| 177.24 \| \| 200.73 \| \| 223.67 \| \| 133.9 \| \| 98.79 \| \| 65.42 \| \| 311.4 \| \| 207.77 \| \| 147.61 \| \| 1,963.94 \| \| 151.49 \| \| 76.87 \| \| 35.93 \| \| 27.42 \| \| 46.65 \| \| 15.4 \| \| 56.58 \| \| 93.08 \| \| 31.17 \| \| 135.46 \| \| 0 \| \| 31.3 \| \| 129.16 \| \| 80.16 \| \| 1,119.06 \| \| 5,705.24 \| \| 665.66 \| \| 310.23 \| \| 296.26 \| \| 511.22 \| \| 209.61 \| \| 277.73 \| \| 65.69 \| \| 82.21 \| \| 96.65 \| \| 414.85 \| \| 87.87 \| \| 204.45 \| \| 329.67 \| \| 47.81 \| \| 238.27 \| \| 81.98 \| \| 59.22 \| \| 47.74 \| \| 326.1 \| \| 79.49 \| \| 344.92 \| \| 51.76 \| \| 79.37 \| \| 46.38 \| \| 84.79 \| \| 924.92 \| \| 98.12 \| \| 18.65 \| \| 34.86 \| \| 66.63 \| \| 143.61 \| \| 105.12 \| \| 107.8 \| \| 132.51 \| \| 134.11 \| \| 80.32 \| \| 40.61 \| \| 37.08 \| \| 73.82 \| \| 156.15 \| \| 300.09 \| \| 73.47 \| \| 320.23 \| \| 1,999.08 \| \| 1,930.98 \| \| 139.66 \| \| 247.84 \| \| 328.25 \| \| 34.88 \| \| 1,165.91 \| \| 588.27 \| \| 440.18 \| \| 220.88 \| \| 63.13 \| \| 15.2 \| \| 129.55 \| \| 509.42 \| \| 219.79 \| \| 156.8 \| \| 80.57 \| \| 146.19 \| \| 304.3 \| \| 75.97 \| \| 0 \| \| 1,181.74 \| \| 70.79 \| \| 268.07 \| \| 213.4 \| \| 106.57 \| \| 6.44 \| \| 104.91 \| \| 282.11 \| \| 130.35 \| \| 376.9 \| \| 138.96 \| \| 174.11 \| \| 113.96 \| \| 31.51 \| \| 28.14 \| \| 37.69 \| \| 48.56 \| \| 112.29 \| \| 54.89 \| \| 88.93 \| \| 370.29 \| \| 323.96 \| \| 1,739.58 \| \| 207 \| \| 243.29 \| \| 416.66 \| \| 125.18 \| \| 73.97 \| \| 1,621.49 \| \| 19,185.63 \| \| 230.21 \| \| 415.45 \| \| 148.51 \| \| 128.6 \| \| 159.05 \| \| 31.88 \| \| 81.3 \| \| 545.95 \| \| 125.19 \| \| 54.65 \| \| 99.21 \| \| 55.67 \| \| 55.34 \| \| 81.02 \| \| 202.67 \| \| 873.73 \| \| 682.52 \| \| 575.91 \| \| 463.14 \| \| 1,080.97 \| \| 170.24 \| \| 246.18 \| \| 42.72 \| \| 2.59 \| \| 0 \| \| 0 \| \| 1.78 \| \| 0 \| \| 13.07 \| \| 1,543.35 \| \| 1,873.84 \| \| 298.26 \| \| 245.15 \| \| 986.9 \| \| 172.69 \| \| 609.06 \| \| 133.05 \| \| 233.51 \| \| 187.77 \| \| 144.73 \| \| 274.76 \| \| 135.54 \| \| 129.56 \| \| 0 \| \| 405.44 \| \| 0 \| \| 0 \| \| 210.34 \| \| 155.02 \| \| 68 \| \| 100.22 \| \| 71.95 \| \| 221.7 \| \| 118.15 \| \| 375.84 \| \| 61.48 \| \| 220.22 \| \| 304.55 \| \| 104.53 \| \| 91.47 \| \| 80.18 \| \| 294.45 \| \| 202.49 \| \| 265.32 \| \| 117.91 \| \| 115.02 \| \| 380.05 \| \| 768.97 \| \| 63.28 \| \| 425.92 \| \| 75.57 \| \| 113.75 \| \| 140.18 \| \| 193.85 \| \| 56.88 \| \| 133.65 \| \| 176.91 \| \| 100.54 \| \| 195.93 \| \| 1,532.69 \| \| 161.2 \| \| 99.51 \| \| 218.16 \| \| 113.87 \| \| 31.89 \| \| 19.74 \| \| 33.04 \| \| 13.82 \| \| 26.95 \| \| 404.87 \| \| 42.81 \| \| 43.12 \| \| 14.2 \| \| 13.8 \| \| 1,065.34 \| \| 172.36 \| \| 10.66 \| \| 1,711.99 \| \| 237.16 \| \| 369.87 \| \| 45.72 \| \| 87.82 \| \| 57.91 \| \| 0 \| \| 28.81 \| \| 31.18 \| \| 29.98 \| \| 17.97 \| \| 17.25 \| \| 34.85 \| \| 14.1 \| \| 64.04 \| \| 48.03 \| \| 99.58 \| \| 38.44 \| \| 108.16 \| \| 118.64 \| \| 204.45 \| \| 535.67 \| \| 696.7 \| \| 729.84 \| \| 38.5 \| \| 98.34 \| \| 235.33 \| \| 78.42 \| \| 163.15 \| \| 339.73 \| \| 70.54 \| \| 238.64 \| \| 7.53 \| \| 673.74 \| \| 49.81 \| \| 274.75 \| \| 38.42 \| \| 127.67 \| \| 24.19 \| \| 21.84 \| \| 49.68 \| \| 11 \| \| 15.82 \| \| 10.47 \| \| 25.23 \| \| 18.18 \| \| 75.05 \| \| 244.97 \| \| 276.47 \| \| 107.8 \| \| 62.88 \| \| 127.7 \| \| 791.57 \| \| 173.31 \| \| 166.48 \| \| 94.04 \| \| 76.49 \| \| 49.05 \| \| 230.36 \| \| 611.5 \| \| 46.92 \| \| 15.83 \| \| 19.23 \| \| 85.6 \| \| 18.81 \| \| 13.05 \| \| 21.68 \| \| 110.52 \| \| 62.88 \| \| 208.9 \| \| 50.38 \| \| 69.25 \| \| 28.71 \| \| 75.36 \| \| 25.59 \| \| 15.8 \| \| 97.91 \| \| 54.45 \| \| 60.16 \| \| 97.85 \| \| 132.57 \| \| 49.57 \| \| 39.93 \| \| 313.74 \| \| 404.14 \| \| 184.3 \| \| 411.88 \| \| 212.23 \| \| 35.69 \| \| 34.15 \| \| 20.51 \| \| 37.41 \| \| 0 \| \| 36.61 \| \| 0 \| \| 47.74 \| \| 228.79 \| \| 144.08 \| \| 224.14 \| \| 0 \| \| 0 \| \| 0 \| \| 28.87 \| \| 84.84 \| \| 17.97 \| \| 0 \| \| 30.63 \| \| 38.15 \| \| 68.68 \| \| 92.4 \| \| 63.89 \| \| 101.78 \| \| 72.61 \| \| 55.75 \| \| 83.94 \| \| 41.97 \| \| 127.72 \| \| 103.42 \| \| 93.16 \| \| 59.98 \| \| 19.36 \| \| 184.8 \| \| 278.66 \| \| 195.14 \| \| 132.22 \| \| 104.7 \| \| 251.65 \| \| 53.11 \| \| 89.74 \| \| 423.62 \| \| 488.13 \| \| 366.15 \| \| 653.75 \| \| 55.71 \| \| 617.51 \| \| 390.42 \| \| 731.45 \| \| 20.08 \| \| 41.26 \| \| 52.5 \| \| 35.93 \| \| 66.42 \| \| 421.93 \| \| 203.89 \| \| 22.8 \| \| 193.22 \| \| 329.14 \| \| 747.66 \| \| 698.57 \| \| 79.24 \| \| 37.17 \| \| 74.11 \| \| 140.67 \| \| 1,066.45 \| \| 1,759.37 \| \| 2,032.62 \| \| 458.64 \| \| 172.28 \| \| 357.05 \| \| 353.86 \| \| 99.87 \| \| 415.39 \| \| 529.51 \| \| 303.26 \| \| 60.73 \| \| 192.78 \| \| 94.63 \| \| 230.71 \| \| 108.62 \| \| 183.03 \| \| 73.12 \| \| 103.63 \| \| 190 \| \| 316.03 \| \| 144.87 \| \| 72.8 \| \| 157.74 \| \| 77.09 \| \| 227.22 \| \| 324.02 \| \| 385.97 \| \| 696.95 \| \| 384.24 \| \| 15.94 \| \| 15.72 \| \| 5.23 \| \| 8.23 \| \| 27.16 \| \| 186.55 \| \| 123.1 \| \| 115.07 \| \| 187.7 \| \| 55.46 \| \| 719.42 \| \| 1,826.83 \| \| 1,247.17 \| \| 1,909.25 \| \| 621.07 \| \| 1,269.55 \| \| 2,303.94 \| \| 1,132.90 \| \| 405.95 \| \| 123.14 \| \| 609.79 \| \| 866.53 \| \| 49.58 \| \| 1,664.27 \| \| 88.69 \| \| 96.8 \| \| 433.64 \| \| 206.36 \| \| 253.47 \| \| 293.61 \| \| 140.49 \| \| 115.85 \| \| 225.89  90.5 \| \| 120.73 \| \| 163.29 \| \| 80.16 \| \| 137.88 \| \| 37.26 \| \| 205.07 \| \| 5.28 \| \| 24.59 \| \| 34.39 \| \| 69.1 \| \| 23.1 \| \| 77.26 \| \| 131.16 \| \| 162.85 \| \| 206.77 \| \| 60.68 \| \| 22.95 \| \| 46.27 \| \| 22.63 \| \| 24.82 \| \| 38.59 \| \| 57.93 \| \| 160.11 \| \| 303.38 \| \| 57.6 \| \| 72.81 \| \| 86.14 \| \| 65.86 \| \| 11.98 \| \| 0 \| \| 15.4 \| \| 0 \| \| 805.35 \| \| 44.73 \| \| 134.23 \| \| 229.82 \| \| 47.71 \| \| 50.01 \| \| 138.64 \| \| 155.97 \| \| 146.71 \| \| 0 \| \| 43.95 \| \| 81.33 \| \| 142.45 \| \| 544.67 \| \| 470.27 \| \| 37.56 \| \| 639.29 \| \| 282.49 \| \| 235.01 \| \| 194.58 \| \| 139.5 \| \| 64.21 \| \| 100.44 \| \| 128.61 \| \| 60.73 \| \| 257.14 \| \| 117.28 \| \| 110.56 \| \| 141.91 \| \| 87.16 \| \| 219.23 \| \| 204.4 \| \| 104.01 \| \| 48.94 \| \| 61.2 \| \| 0 \| \| 25.78 \| \| 76.03 \| \| 185.49 \| \| 45.13 \| \| 39.28 \| \| 68.08 \| \| 50.73 \| \| 53.28 \| \| 65.78 \| \| 19.74 \| \| 78.9 \| \| 175.44 \| \| 5,973.43 \| \| 1,857.28 \| \| 39.54 \| \| 62.01 \| \| 151.95 \| \| 79.13 \| \| 16.21 \| \| 24.12 \| \| 45.1 \| \| 48.77 \| \| 31.88 \| \| 33.78 \| \| 9.94 \| \| 21.01 \| \| 41.04 \| \| 45.63 \| \| 45.06 \| \| 26.01 \| \| 66.71 \| \| 240.64 \| \| 36.84 \| \| 0 \| \| 48.43 \| \| 13.19 \| \| 80.12 \| \| 347.65 \| \| 405.43 \| \| 276.83 \| \| 145.49 \| \| 86.03 \| \| 70.74 \| \| 228.49 \| \| 62.99 \| \| 48.25 \| \| 28.06 \| \| 40.79 \| \| 82.7 \| \| 105.18 \| \| 186.81 \| \| 126.69 \| \| 178.69 \| \| 233.84 \| \| 876.05 \| \| 230.09 \| \| 136.57 \| \| 142.25 \| \| 310.69 \| \| 392.95 \| \| 339.08 \| \| 388.03 \| \| 68.58 \| \| 271.74 \| \| 55.3 \| \| 56.59 \| \| 6.98 \| \| 12.96 \| \| 12.76 \| \| 913.02 \| \| 368.16 \| \| 172.7 \| \| 202.17 \| \| 169.54 \| \| 97.93 \| \| 51.3 \| \| 51.78 \| \| 136.46 \| \| 85.02 \| \| 112.59 \| \| 104.43 \| \| 727.93 \| \| 105.52 \| \| 251.65 \| \| 444.76 \| \| 424.61 \| \| 157.87 \| \| 97.37 \| \| 127.16 \| \| 243.19 \| \| 136.96 \| \| 1,144.59 \| \| 83.16 \| \| 224.45 \| \| 0 \| \| 0 \| \| 1,760.17 \| \| 1,506.92 \| \| 340.44 \| \| 76.57 \| \| 715.13 \| \| 70.3 \| \| 98.89 \| \| 107.35 \| \| 172.89 \| \| 103.73 \| \| 103.4 \| \| 124.38 \| \| 186.24 \| \| 124.49 \| \| 145.29 \| \| 287.46 \| \| 71.57 \| \| 375.04 \| \| 100.48 \| \| 72.63 \| \| 60.89 \| \| 50.74 \| \| 53.58 \| \| 31.77 \| \| 70.48 \| \| 82.69 \| \| 103.86 \| \| 53 \| \| 864.88 \| \| 1,841.64 \| \| 96.55 \| \| 45.69 \| \| 59.86 \| \| 197.99 \| \| 304.86 \| \| 76.05 \| \| 1,617.28 \| \| 1,454.68 \| \| 594.72 \| \| 1,338.93 \| \| 15.88 \| \| 36.09 \| \| 69.15 \| \| 39.72 \| \| 112.33 \| \| 44.53 \| \| 63.39 \| \| 81.14 \| \| 266.4 \| \| 60.76 \| \| 15.51 \| \| 278.54 \| \| 67.65 \| \| 20 \| \| 49 \| \| 35.37 \| \| 3.83 \| \| 32.24 \| \| 179.66 \| \| 198.14 \| \| 174.68 \| \| 271.95 \| \| 915.78 \| \| 305.27 \| \| 145.02 \| \| 104.85 \| \| 71.62 \| \| 0 \| \| 0 \| \| 0 \| \| 25.21 \| \| 32.73 \| \| 29.79 \| \| 164.13 \| \| 73.18 \| \| 0 \| \| 46.57 \| \| 30.08 \| \| 20.08 \| \| 0 \| \| 61.71 \| \| 95.38 \| \| 63.4 \| \| 40.06 \| \| 38.68 \| \| 54.72 \| \| 38.18 \| \| 22.32 \| \| 25.25 \| \| 21.86 \| \| 43.02 \| \| 31.16 \| \| 96.15 \| \| 22.02 \| \| 14.01 \| \| 22.63 \| \| 48.68 \| \| 20.05 \| \| 0 \| \| 19.14 \| \| 21.26 \| \| 57.27 \| \| 44.59 \| \| 51.88 \| \| 76.28 \| \| 43.33 \| \| 57.64 \| \| 100.2 \| \| 0 \| \| 151.64 \| \| 66.89 \| \| 103.2 \| \| 53.9 \| \| 21.98 \| \| 52.19 \| \| 75.58 \| \| 25.75 \| \| 25.25 \| \| 31.36 \| \| 26.38 \| \| 38.27 \| \| 90.32 \| \| 71.39 \| \| 71.57 \| \| 71.41 \| \| 184.55 \| \| 92.93 \| \| 177.87 \| \| 88.98 \| \| 67.62 \| \| 86.24 \| \| 24.64 \| \| 190.25 \| \| 48.23 \| \| 72.62 \| \| 14.06 \| \| 25.98 \| \| 24.06 \| \| 28.19 \| \| 80.85 \| \| 44.36 \| \| 48.92 \| \| 57.74 \| \| 65.39 \| \| 161.53 \| \| 159.68 \| \| 12.81 \| \| 90.86 \| \| 24.44 \| \| 21.25 \| \| 19.18 \| \| 44.92 \| \| 30.46 \| \| 52.72 \| \| 133.38 \| \| 69.83 \| \| 53.09 \| \| 48.62 \| \| 46.94 \| \| 62.91 \| \| 49.29 \| \| 0.74 \| \| 99.31 \| \| 396.91 \| \| 761.93 \| \| 74.21 \| \| 144.09 \| \| 70.34 \| \| 323.01 \| \| 96.94 \| \| 129.88 \| \| 200.24 \| \| 159.23 \| \| 244.77 \| \| 231.8 \| \| 146.61 \| \| 129.73 \| \| 268.92 \| \| 148.4 \| \| 95.3 \| \| 54.06 \| \| 6.34 \| \| 13.37 \| \| 45.34 \| \| 68.5 \| \| 91.35 \| \| 79.84 \| \| 14.65 \| \| 22.66 \| \| 36.12 \| \| 38.16 \| \| 303.56 \| \| 206.59 \| \| 113.52 \| \| 66.95 \| \| 130.19 \| \| 13.61 \| \| 98.95 \| \| 127.81 \| \| 100.9 \| \| 103.61 \| \| 0 \| \| 111.19 \| \| 210.87 \| \| 0 \| \| 221.06 \| \| 149.65 \| \| 114.94 \| \| 63.59 \| \| 174.89 \| \| 51.93 \| \| 26.88 \| \| 126.82 \| \| 0 \| \| 0 \| \| 0 \| \| 106.42 \| \| 0 \| \| 0 \| \| 0 \| \| 0 \| \| 0 \| \| 115.74 \| \| 77.37 \| \| 77.99 \| \| 194.1 \| \| 262.68 \| \| 267.66 \| \| 110.67 \| \| 110.89 \| \| 40.42 \| \| 77.23 \| \| 32.46 \| \| 85.57 \| \| 55.26 \| \| 91.89 \| \| 52.4 \| \| 69.02 \| \| 29.87 \| \| 50.33 \| \| 128.93 \| \| 103.11 \| \| 162.95 \| \| 97.77 \| \| 59.98 \| \| 89.98 \| \| 60.28 \| \| 92.3 \| \| 0 \| \| 0 \| \| 23.96 \| \| 24.23 \| \| 27.44 \| \| 93.49 \| \| 23.1 \| \| 43.81 \| \| 31.13 \| \| 57.51 \| \| 50.83 \| \| 47.36 \| \| 92.3 \| \| 202.08 \| \| 468.64 \| \| 3,389.52 \| \| 252.89 \| \| 0 \| \| 67.25 \| \| 279.01 \| \| 186.81 \| \| 58.34 \| \| 88.2 \| \| 1,299.04 \| \| 77.72 \| \| 111.6 \| \| 138.55 \| \| 43.77 \| \| 455.47 \| \| 70.15 \| \| 69.99 \| \| 52.59 \| \| 71.14 \| \| 56.69 \| \| 133.74 \| \| 102.11 \| \| 129.8 \| \| 219.1 \| \| 272.33 \| \| 178.15 \| \| 43.66 \| \| 154.56 \| \| 165.91 \| \| 27.75 \| \| 43.78 \| \| 58.43 \| \| 560.45 \| \| 203.46 \| \| 59.51 \| \| 57.23 \| \| 152.66 \| \| 6.7 \| \| 0.22 \| \| 0 \| \| 143.68 \| \| 192.76 \| \| 210.77 \| \| 189.71 \| \| 79.79 \| \| 126.41 \| \| 90.18 \| \| 169.46 \| \| 207.9 \| \| 291.06 \| \| 505.71 \| \| 115.52 \| \| 68.46 \| \| 72.02 \| \| 0 \| \| 3,507.45 \| \| 0 \| \| 0 \| \| 184.59 \| \| 2.53 \| \| 3,516.53 \| \| 0 \| \| 0 \| \| 195 \| \| 82.92 \| \| 74.68 \| \| 141.74 \| \| 131.5 \| \| 31.84 \| \| 31.78 \| \| 85.87 \| \| 147.3 \| \| 62.71 \| \| 110.67 \| \| 68.64 \| \| 106.48 \| \| 311.77 \| \| 825.51 \| \| 77 \| \| 123.3 \| \| 62.7 \| \| 54.06 \| \| 38.9 \| \| 36.88 \| \| 80.63 \| \| 87.68 \| \| 98.91 \| \| 402.09 \| \| 167.74 \| \| 234.08 \| \| 527.29 \| \| 96.52 \| \| 357.42 \| \| 235.74 \| \| 79.43 \| \| 232.8 \| \| 37.89 \| \| 27.91 \| \| 36.83 \| \| 68.27 \| \| 26.76 \| \| 0 \| \| 251.15 \| \| 169.51 \| \| 514.67 \| \| 248.51 \| \| 190.43 \| \| 135.04 \| \| 62.52 \| \| 53.23 \| \| 251.59 \| \| 292.04 \| \| 245.41 \| |
